# Supplementary material for: Characterization of the first vaginal Lactobacillus crispatus genomes isolated in Brazil
Source: PeerJ. 2021 Mar 10;9:e11079. doi: 10.7717/peerj.11079 (PMC7955673; doi:10.7717/peerj.11079)
Supplement: Supplemental Information 2 [file peerj-09-11079-s002.pdf]

TYPE (STRAIN) GENOME SERVER

JOB SUMMARY

PRINT DATE:	2020-05-25 04:06:15 +0200
JOB ID:	41970917-4174-42a1-ae04-923aed79d664
RESULT PAGE:	https://tygs.dsmz.de/user_results/show?guid=41970917-4174-42a1-ae04-923aed79d664

Table 1: Phylogenies

Publication-ready versions of both the genome-scale GBDP tree and the 16S rRNA gene sequence tree can be customized and exported either in SVG (vector graphic) or PNG format from within the phylogeny viewers in your TYGS result page. For publications the **SVG format is recommended** because it is lossless, always keeps its high resolution and can also be easily converted to other popular formats such as PDF or EPS. Please follow the link provided above!

Table 2: Identification

The below list contains the result of the TYGS species identification routine.

Explanation of remarks that might occur in the below table:

remark [R1]: The TYGS type strain database is automatically updated on an almost daily basis. However, if a particular type strain genome is not available in the TYGS database, this can have several reasons which are detailed in the FAQ. You can request an extended 16S rRNA gene analysis via the 16S tree viewer found in your result page to detect **not yet genome-sequenced** type strains relevant for your study.

remark [R2]: > 70% dDDH value (formula d_4) and (almost) minimal dDDH values for gene-content formulae d_0 and d_6 indicate a potentially unreliable identification result and should thus be checked via the 16S rRNA gene sequence similarity. Such strong deviations can, in principle, be caused by sequence contamination.

remark [R3]: G+C content difference of > 1 % indicates a potentially unreliable identification result because within species G+C content varies no more than 1 %, if computed from genome sequences (PMID: 24505073).

Strain	Conclusion	Identification result	Remark
**'Lc1252CHN'**	belongs to known species	*Lactobacillus crispatus*	
**'Lc2029'**	belongs to known species	*Lactobacillus crispatus*	
**'LcAB70'**	belongs to known species	*Lactobacillus crispatus*	
**'LcCIP104459'**	belongs to known species	*Lactobacillus crispatus*	
**'LcCRI4'**	belongs to known species	*Lactobacillus crispatus*	
**'LcCRI8'**	belongs to known species	*Lactobacillus crispatus*	
**'LcCRI10'**	belongs to known species	*Lactobacillus crispatus*	
**'LcCRI17'**	belongs to known species	*Lactobacillus crispatus*	
**'LcCTV05'**	belongs to known species	*Lactobacillus crispatus*	
**'LcJVV01'**	belongs to known species	*Lactobacillus crispatus*	
**'LcMV1AUS'**	belongs to known species	*Lactobacillus crispatus*	
**'LcMV3AUS'**	belongs to known species	*Lactobacillus crispatus*	
**'LcRL02'**	belongs to known species	*Lactobacillus crispatus*	
**'LcRL03'**	belongs to known species	*Lactobacillus crispatus*	
**'LcRL05'**	belongs to known species	*Lactobacillus crispatus*	

Strain	Conclusion	Identification result	Remark
**'LcRL06'**	belongs to known species	*Lactobacillus crispatus*	
**'LcRL07'**	belongs to known species	*Lactobacillus crispatus*	
**'LcRL08'**	belongs to known species	*Lactobacillus crispatus*	
**'LcRL09'**	belongs to known species	*Lactobacillus crispatus*	
**'LcRL10'**	belongs to known species	*Lactobacillus crispatus*	

Table 3: Pairwise comparisons of user genomes vs. type-strain genomes

The following table contains the pairwise dDDH values between your user genomes and the selected type-strain genomes. The dDDH values are provided along with their confidence intervals (C.I.) for the three different GBDP formulas:

• formula d_0 (a.k.a. GGDC formula 1): length of all HSPs divided by total genome length
• formula d_4 (a.k.a. GGDC formula 2): sum of all identities found in HSPs divided by overall HSP length
• formula d_6 (a.k.a. GGDC formula 3): sum of all identities found in HSPs divided by total genome length

Note: Formula d_4 is independent of genome length and is thus robust against the use of incomplete draft genomes. For other reasons for preferring formula d_4, see the FAQ.

Query	Subject	d_0	C.I. d_0	d_4	C.I. d_4	d_6	C.I. d_6	Diff. G+C Percent
**'LcRL09.fna'**	**'LcRL10.fna'**	98.4	[97.2 - 99.1]	99.9	[99.8 - 99.9]	99.3	[98.7 - 99.6]	0.16
**'LcRL02.fna'**	**'LcRL10.fna'**	98.7	[97.7 - 99.2]	99.9	[99.8 - 99.9]	99.4	[99.0 - 99.7]	0.11
**'LcRL06.fna'**	**'LcRL07.fna'**	99.6	[99.1 - 99.8]	99.9	[99.9 - 100.0]	99.8	[99.7 - 99.9]	0.0
**'LcRL02.fna'**	**'LcRL09.fna'**	99.2	[98.5 - 99.6]	99.9	[99.9 - 100.0]	99.7	[99.4 - 99.8]	0.05
**'LcCRI17.fasta'**	**'LcRL07.fna'**	92.4	[89.5 - 94.5]	99.5	[99.2 - 99.7]	95.6	[93.8 - 96.9]	0.07
**'LcCRI17.fasta'**	**'LcRL06.fna'**	92.0	[89.1 - 94.2]	99.5	[99.1 - 99.7]	95.3	[93.5 - 96.7]	0.07
**'LcCRI8.fasta'**	**'LcRL07.fna'**	89.2	[85.9 - 91.9]	99.3	[98.9 - 99.6]	93.4	[91.1 - 95.1]	0.01
**'LcJVV01.fna'**	**'LcMV1AUS.fna'**	81.7	[77.8 - 85.1]	99.2	[98.7 - 99.5]	87.6	[84.6 - 90.1]	0.02
**'LcCRI8.fasta'**	**'LcRL06.fna'**	88.8	[85.4 - 91.5]	99.2	[98.8 - 99.5]	93.0	[90.7 - 94.8]	0.01
**'LcCRI10.fasta'**	**'LcRL06.fna'**	92.3	[89.5 - 94.5]	99.1	[98.6 - 99.4]	95.5	[93.6 - 96.8]	0.06
**'LcCRI10.fasta'**	**'LcRL07.fna'**	92.8	[90.0 - 94.8]	99.1	[98.6 - 99.4]	95.7	[94.0 - 97.0]	0.06
**'LcRL07.fna'**	**'LcRL08.fna'**	93.8	[91.3 - 95.7]	99.0	[98.5 - 99.4]	96.4	[94.9 - 97.5]	0.12
**'LcRL06.fna'**	**'LcRL08.fna'**	93.5	[90.8 - 95.4]	99.0	[98.5 - 99.4]	96.2	[94.6 - 97.4]	0.12
**'LcCRI10.fasta'**	**'LcCRI17.fasta'**	93.1	[90.4 - 95.1]	98.8	[98.2 - 99.2]	95.9	[94.2 - 97.2]	0.13
**'LcCRI17.fasta'**	**'LcRL08.fna'**	92.9	[90.2 - 95.0]	98.7	[98.0 - 99.1]	95.8	[94.1 - 97.1]	0.2
**'LcRL05.fna'**	**'LcRL07.fna'**	81.7	[77.9 - 85.1]	98.6	[98.0 - 99.1]	87.5	[84.5 - 90.0]	0.46
**'LcRL05.fna'**	**'LcRL06.fna'**	81.2	[77.3 - 84.6]	98.6	[98.0 - 99.1]	87.1	[84.0 - 89.6]	0.46
**'LcCRI17.fasta'**	**'LcRL05.fna'**	87.0	[83.4 - 89.9]	98.4	[97.7 - 98.9]	91.6	[89.0 - 93.6]	0.39
**'LcCRI4.fasta'**	**'LcRL02.fna'**	98.3	[97.1 - 99.0]	98.1	[97.2 - 98.7]	99.0	[98.4 - 99.4]	0.04
**'LcCRI8.fasta'**	**'LcCRI17.fasta'**	91.2	[88.1 - 93.5]	98.1	[97.2 - 98.7]	94.5	[92.5 - 96.0]	0.06
**'LcCRI8.fasta'**	**'LcRL05.fna'**	79.9	[75.9 - 83.3]	98.1	[97.3 - 98.7]	85.8	[82.7 - 88.5]	0.45
**'LcCRI4.fasta'**	**'LcRL09.fna'**	98.0	[96.7 - 98.8]	98.0	[97.2 - 98.6]	98.9	[98.2 - 99.3]	0.09

Query	Subject	d_0	C.I. d_0	d_4	C.I. d_4	d_6	C.I. d_6	Diff. G+C Percent
**'LcCRI4.fasta'**	**'LcRL10.fna'**	97.2	[95.6 - 98.3]	97.8	[96.8 - 98.4]	98.4	[97.5 - 99.0]	0.07
**'LcCRI10.fasta'**	**'LcRL08.fna'**	96.0	[94.0 - 97.4]	97.8	[96.8 - 98.4]	97.7	[96.5 - 98.5]	0.06
**'LcCRI8.fasta'**	**'LcRL08.fna'**	94.2	[91.7 - 96.0]	97.7	[96.7 - 98.4]	96.5	[94.9 - 97.6]	0.14
**'LcCRI17.fasta'**	**'LcRL03.fna'**	82.4	[78.6 - 85.7]	97.6	[96.6 - 98.3]	87.8	[84.8 - 90.3]	0.02
**'LcCRI8.fasta'**	**'LcRL03.fna'**	88.9	[85.5 - 91.6]	97.5	[96.5 - 98.2]	92.8	[90.5 - 94.6]	0.04
**'LcRL03.fna'**	**'LcRL06.fna'**	84.3	[80.5 - 87.5]	97.5	[96.4 - 98.2]	89.3	[86.5 - 91.6]	0.05
**'LcRL05.fna'**	**'LcRL08.fna'**	83.6	[79.8 - 86.8]	97.4	[96.4 - 98.2]	88.7	[85.8 - 91.1]	0.58
**'LcRL03.fna'**	**'LcRL07.fna'**	84.9	[81.1 - 88.0]	97.4	[96.4 - 98.2]	89.7	[87.0 - 92.0]	0.06
**'LcCRI10.fasta'**	**'LcRL05.fna'**	81.7	[77.8 - 85.0]	97.2	[96.1 - 98.0]	87.2	[84.1 - 89.7]	0.52
**'Lc2029.fna'**	**'LcCRI8.fasta'**	95.3	[93.1 - 96.8]	97.0	[95.9 - 97.8]	97.2	[95.8 - 98.1]	0.13
**'LcJVV01.fna'**	**'LcRL02.fna'**	87.3	[83.7 - 90.1]	97.0	[95.8 - 97.8]	91.5	[89.0 - 93.5]	0.08
**'LcJVV01.fna'**	**'LcRL09.fna'**	86.6	[83.0 - 89.6]	96.9	[95.7 - 97.8]	91.0	[88.4 - 93.1]	0.03
**'LcCRI4.fasta'**	**'LcJVV01.fna'**	89.5	[86.2 - 92.1]	96.9	[95.7 - 97.8]	93.2	[90.9 - 94.9]	0.12
**'Lc2029.fna'**	**'LcRL06.fna'**	89.8	[86.6 - 92.4]	96.8	[95.6 - 97.7]	93.4	[91.1 - 95.1]	0.14
**'LcAB70.fna'**	**'LcRL06.fna'**	88.1	[84.6 - 90.8]	96.7	[95.4 - 97.6]	92.0	[89.6 - 94.0]	0.28
**'LcAB70.fna'**	**'LcRL07.fna'**	88.5	[85.1 - 91.2]	96.7	[95.5 - 97.6]	92.4	[90.0 - 94.3]	0.28
**'Lc2029.fna'**	**'LcRL07.fna'**	90.2	[87.0 - 92.7]	96.7	[95.5 - 97.6]	93.6	[91.4 - 95.3]	0.14
**'LcJVV01.fna'**	**'LcRL10.fna'**	85.5	[81.8 - 88.5]	96.6	[95.3 - 97.5]	90.1	[87.3 - 92.3]	0.19
**'LcCRI8.fasta'**	**'LcCRI10.fasta'**	94.7	[92.3 - 96.3]	96.5	[95.3 - 97.5]	96.7	[95.2 - 97.7]	0.07
**'LcRL03.fna'**	**'LcRL05.fna'**	74.1	[70.1 - 77.7]	96.5	[95.3 - 97.5]	80.5	[77.1 - 83.5]	0.4
**'Lc2029.fna'**	**'LcCRI17.fasta'**	91.0	[87.9 - 93.4]	96.4	[95.1 - 97.4]	94.2	[92.1 - 95.7]	0.07
**'LcRL03.fna'**	**'LcRL08.fna'**	87.2	[83.7 - 90.1]	96.3	[95.0 - 97.3]	91.4	[88.8 - 93.4]	0.18
**'LcAB70.fna'**	**'LcCRI17.fasta'**	86.3	[82.7 - 89.3]	96.2	[94.8 - 97.2]	90.6	[88.0 - 92.8]	0.35
**'LcMV1AUS.fna'**	**'LcRL02.fna'**	84.8	[81.1 - 87.9]	96.0	[94.7 - 97.1]	89.5	[86.6 - 91.7]	0.1
**'Lc2029.fna'**	**'LcRL08.fna'**	96.1	[94.0 - 97.4]	96.0	[94.6 - 97.0]	97.5	[96.3 - 98.4]	0.26
**'LcJVV01.fna'**	**'LcMV3AUS.fna'**	78.3	[74.4 - 81.8]	95.9	[94.5 - 97.0]	84.1	[80.9 - 86.9]	0.16
**'LcMV1AUS.fna'**	**'LcRL09.fna'**	85.4	[81.7 - 88.4]	95.8	[94.4 - 96.9]	89.9	[87.1 - 92.1]	0.05

Query	Subject	d_0	C.I. d_0	d_4	C.I. d_4	d_6	C.I. d_6	Diff. G+C Percent
**'Lc2029.fna'**	**'LcRL03.fna'**	89.6	[86.3 - 92.2]	95.7	[94.3 - 96.8]	93.1	[90.8 - 94.8]	0.09
**'LcAB70.fna'**	**'LcRL08.fna'**	91.2	[88.1 - 93.5]	95.6	[94.1 - 96.7]	94.2	[92.1 - 95.7]	0.16
**'LcCRI10.fasta'**	**'LcRL03.fna'**	89.2	[85.9 - 91.8]	95.6	[94.1 - 96.7]	92.7	[90.4 - 94.6]	0.11
**'Lc2029.fna'**	**'LcRL05.fna'**	79.9	[75.9 - 83.3]	95.6	[94.1 - 96.7]	85.4	[82.2 - 88.1]	0.32
**'LcAB70.fna'**	**'LcCRI10.fasta'**	92.0	[89.1 - 94.2]	95.5	[94.0 - 96.6]	94.7	[92.8 - 96.2]	0.22
**'LcMV1AUS.fna'**	**'LcRL10.fna'**	87.2	[83.6 - 90.1]	95.1	[93.5 - 96.3]	91.1	[88.5 - 93.2]	0.21
**'LcAB70.fna'**	**'LcRL05.fna'**	76.5	[72.5 - 80.1]	95.1	[93.5 - 96.3]	82.4	[79.1 - 85.3]	0.74
**'Lc2029.fna'**	**'LcCRI10.fasta'**	97.6	[96.1 - 98.5]	94.8	[93.1 - 96.0]	98.4	[97.4 - 99.0]	0.2
**'LcCRI4.fasta'**	**'LcMV1AUS.fna'**	86.3	[82.7 - 89.3]	94.8	[93.2 - 96.1]	90.4	[87.7 - 92.6]	0.14
**'LcAB70.fna'**	**'LcCRI8.fasta'**	92.7	[89.9 - 94.8]	94.7	[93.1 - 96.0]	95.1	[93.2 - 96.5]	0.29
**'Lc2029.fna'**	**'LcAB70.fna'**	91.1	[88.0 - 93.4]	94.7	[93.1 - 96.0]	94.0	[91.8 - 95.6]	0.42
**'LcCRI8.fasta'**	**'LcCTV05.fna'**	78.8	[74.8 - 82.2]	94.6	[92.9 - 95.9]	84.3	[81.0 - 87.1]	0.09
**'LcAB70.fna'**	**'LcRL03.fna'**	85.5	[81.8 - 88.6]	94.5	[92.8 - 95.8]	89.8	[87.0 - 92.0]	0.33
**'LcCTV05.fna'**	**'LcRL06.fna'**	80.4	[76.4 - 83.8]	94.4	[92.6 - 95.7]	85.6	[82.4 - 88.3]	0.08
**'LcMV1AUS.fna'**	**'LcMV3AUS.fna'**	80.1	[76.2 - 83.5]	94.4	[92.7 - 95.7]	85.4	[82.2 - 88.1]	0.14
**'LcCTV05.fna'**	**'LcRL07.fna'**	80.9	[77.0 - 84.3]	94.2	[92.5 - 95.6]	86.0	[82.9 - 88.6]	0.08
**'LcAB70.fna'**	**'LcCTV05.fna'**	78.2	[74.2 - 81.7]	94.1	[92.3 - 95.5]	83.7	[80.4 - 86.5]	0.2
**'LcCRI17.fasta'**	**'LcCTV05.fna'**	76.0	[72.0 - 79.6]	93.8	[92.0 - 95.2]	81.8	[78.4 - 84.7]	0.15
**'LcMV3AUS.fna'**	**'LcRL10.fna'**	90.1	[86.8 - 92.6]	93.4	[91.6 - 94.9]	93.1	[90.8 - 94.8]	0.35
**'LcMV3AUS.fna'**	**'LcRL09.fna'**	88.1	[84.6 - 90.9]	93.3	[91.4 - 94.8]	91.6	[89.0 - 93.6]	0.2
**'LcMV3AUS.fna'**	**'LcRL02.fna'**	87.5	[84.0 - 90.4]	93.3	[91.5 - 94.8]	91.2	[88.6 - 93.2]	0.25
**'LcCTV05.fna'**	**'LcRL05.fna'**	67.6	[63.7 - 71.2]	93.2	[91.3 - 94.7]	74.0	[70.5 - 77.2]	0.54
**'Lc1252CHN.fna'**	**'LcJVV01.fna'**	71.0	[67.0 - 74.6]	93.1	[91.2 - 94.7]	77.2	[73.7 - 80.3]	0.21
**'Lc2029.fna'**	**'LcCTV05.fna'**	82.0	[78.1 - 85.3]	92.7	[90.7 - 94.3]	86.7	[83.6 - 89.3]	0.22
**'LcCTV05.fna'**	**'LcRL03.fna'**	77.0	[73.0 - 80.5]	92.4	[90.4 - 94.0]	82.4	[79.1 - 85.3]	0.13
**'LcCRI4.fasta'**	**'LcMV3AUS.fna'**	88.7	[85.3 - 91.4]	92.3	[90.3 - 93.9]	91.9	[89.4 - 93.9]	0.28
**'LcCTV05.fna'**	**'LcRL08.fna'**	79.5	[75.6 - 83.0]	92.2	[90.1 - 93.8]	84.5	[81.3 - 87.3]	0.04

Query	Subject	d_0	C.I. d_0	d_4	C.I. d_4	d_6	C.I. d_6	Diff. G+C Percent
**'Lc1252CHN.fna'**	**'LcMV1AUS.fna'**	72.2	[68.3 - 75.9]	91.5	[89.3 - 93.2]	78.0	[74.6 - 81.1]	0.19
**'LcAB70.fna'**	**'LcRL02.fna'**	86.7	[83.1 - 89.6]	91.4	[89.2 - 93.2]	90.2	[87.5 - 92.4]	0.29
**'LcAB70.fna'**	**'LcRL09.fna'**	86.5	[82.9 - 89.5]	91.4	[89.3 - 93.2]	90.1	[87.4 - 92.3]	0.34
**'LcCRI10.fasta'**	**'LcCTV05.fna'**	84.7	[81.0 - 87.9]	91.2	[89.0 - 93.0]	88.7	[85.8 - 91.0]	0.02
**'LcAB70.fna'**	**'LcRL10.fna'**	87.9	[84.4 - 90.7]	91.1	[88.9 - 92.9]	91.1	[88.5 - 93.2]	0.18
**'Lc1252CHN.fna'**	**'LcMV3AUS.fna'**	70.8	[66.9 - 74.5]	91.0	[88.8 - 92.8]	76.7	[73.2 - 79.8]	0.05
**'LcMV3AUS.fna'**	**'LcRL06.fna'**	78.6	[74.6 - 82.1]	90.8	[88.5 - 92.6]	83.5	[80.3 - 86.4]	0.26
**'Lc2029.fna'**	**'LcRL10.fna'**	88.8	[85.4 - 91.5]	90.7	[88.5 - 92.6]	91.8	[89.2 - 93.7]	0.24
**'LcMV3AUS.fna'**	**'LcRL07.fna'**	78.9	[75.0 - 82.4]	90.7	[88.4 - 92.5]	83.8	[80.6 - 86.6]	0.26
**'Lc2029.fna'**	**'LcRL02.fna'**	86.5	[82.9 - 89.4]	90.6	[88.3 - 92.4]	89.9	[87.2 - 92.2]	0.13
**'Lc1252CHN.fna'**	**'LcCRI4.fasta'**	77.5	[73.5 - 81.0]	90.5	[88.2 - 92.4]	82.5	[79.2 - 85.4]	0.33
**'Lc2029.fna'**	**'LcRL09.fna'**	87.1	[83.6 - 90.0]	90.5	[88.2 - 92.3]	90.4	[87.7 - 92.6]	0.08
**'LcCRI8.fasta'**	**'LcRL02.fna'**	84.4	[80.7 - 87.6]	90.4	[88.1 - 92.3]	88.3	[85.4 - 90.7]	0.0
**'Lc1252CHN.fna'**	**'LcRL02.fna'**	76.9	[72.9 - 80.5]	90.3	[88.0 - 92.2]	82.0	[78.7 - 84.9]	0.29
**'Lc1252CHN.fna'**	**'LcRL09.fna'**	77.3	[73.3 - 80.8]	90.2	[87.9 - 92.1]	82.3	[79.0 - 85.2]	0.24
**'LcCRI8.fasta'**	**'LcRL09.fna'**	84.7	[81.0 - 87.8]	90.1	[87.8 - 92.0]	88.5	[85.6 - 90.9]	0.05
**'Lc1252CHN.fna'**	**'LcRL10.fna'**	75.5	[71.5 - 79.1]	90.1	[87.8 - 92.0]	80.7	[77.4 - 83.7]	0.4
**'LcJVV01.fna'**	**'LcRL05.fna'**	62.4	[58.7 - 66.0]	90.0	[87.7 - 91.9]	68.6	[65.1 - 71.8]	0.36
**'LcMV3AUS.fna'**	**'LcRL08.fna'**	81.4	[77.5 - 84.8]	89.8	[87.5 - 91.7]	85.8	[82.6 - 88.4]	0.38
**'LcMV3AUS.fna'**	**'LcRL03.fna'**	77.0	[73.0 - 80.6]	89.8	[87.4 - 91.7]	82.0	[78.7 - 84.9]	0.2
**'LcRL02.fna'**	**'LcRL06.fna'**	87.7	[84.2 - 90.5]	89.6	[87.2 - 91.5]	90.7	[88.1 - 92.8]	0.01
**'LcJVV01.fna'**	**'LcRL08.fna'**	73.5	[69.6 - 77.2]	89.6	[87.2 - 91.6]	78.9	[75.5 - 82.0]	0.22
**'LcJVV01.fna'**	**'LcRL07.fna'**	77.8	[73.8 - 81.3]	89.6	[87.3 - 91.6]	82.6	[79.3 - 85.5]	0.1
**'LcCRI8.fasta'**	**'LcRL10.fna'**	85.4	[81.7 - 88.4]	89.6	[87.2 - 91.5]	88.9	[86.0 - 91.2]	0.11
**'LcRL06.fna'**	**'LcRL10.fna'**	85.7	[82.1 - 88.8]	89.5	[87.1 - 91.5]	89.2	[86.3 - 91.5]	0.1
**'LcMV1AUS.fna'**	**'LcRL07.fna'**	72.8	[68.8 - 76.4]	89.5	[87.2 - 91.5]	78.2	[74.8 - 81.3]	0.11
**'LcRL02.fna'**	**'LcRL07.fna'**	88.3	[84.8 - 91.0]	89.5	[87.2 - 91.5]	91.2	[88.6 - 93.2]	0.01

Query	Subject	d_0	C.I. d_0	d_4	C.I. d_4	d_6	C.I. d_6	Diff. G+C Percent
**'LcAB70.fna'**	**'LcCRI4.fasta'**	86.5	[82.9 - 89.5]	89.5	[87.1 - 91.5]	89.8	[87.1 - 92.1]	0.25
**'LcCRI10.fasta'**	**'LcRL02.fna'**	86.2	[82.6 - 89.2]	89.5	[87.2 - 91.5]	89.6	[86.8 - 91.8]	0.07
**'Lc2029.fna'**	**'LcMV3AUS.fna'**	80.6	[76.7 - 84.0]	89.5	[87.1 - 91.5]	85.0	[81.8 - 87.7]	0.12
**'LcRL07.fna'**	**'LcRL10.fna'**	86.3	[82.7 - 89.3]	89.5	[87.1 - 91.5]	89.6	[86.8 - 91.9]	0.1
**'Lc2029.fna'**	**'LcJVV01.fna'**	73.5	[69.5 - 77.1]	89.5	[87.2 - 91.5]	78.9	[75.5 - 82.0]	0.05
**'LcRL07.fna'**	**'LcRL09.fna'**	88.7	[85.3 - 91.4]	89.4	[87.1 - 91.4]	91.5	[88.9 - 93.5]	0.06
**'LcCRI10.fasta'**	**'LcRL09.fna'**	87.0	[83.4 - 89.9]	89.4	[87.0 - 91.4]	90.1	[87.4 - 92.3]	0.12
**'LcRL02.fna'**	**'LcRL03.fna'**	79.3	[75.3 - 82.7]	89.4	[87.0 - 91.4]	83.9	[80.6 - 86.7]	0.04
**'LcRL03.fna'**	**'LcRL10.fna'**	81.8	[77.9 - 85.1]	89.4	[87.1 - 91.4]	86.0	[82.9 - 88.6]	0.15
**'LcJVV01.fna'**	**'LcRL06.fna'**	77.1	[73.1 - 80.7]	89.4	[87.0 - 91.4]	82.0	[78.7 - 84.9]	0.09
**'LcRL06.fna'**	**'LcRL09.fna'**	88.1	[84.6 - 90.9]	89.4	[87.0 - 91.4]	91.0	[88.4 - 93.1]	0.06
**'LcCRI17.fasta'**	**'LcRL02.fna'**	81.2	[77.3 - 84.6]	89.3	[86.9 - 91.3]	85.5	[82.3 - 88.2]	0.06
**'LcMV3AUS.fna'**	**'LcRL05.fna'**	67.7	[63.9 - 71.4]	89.3	[86.9 - 91.3]	73.6	[70.1 - 76.8]	0.2
**'LcMV1AUS.fna'**	**'LcRL06.fna'**	72.0	[68.1 - 75.7]	89.3	[87.0 - 91.3]	77.5	[74.1 - 80.7]	0.11
**'LcCRI17.fasta'**	**'LcMV3AUS.fna'**	77.0	[73.0 - 80.5]	89.2	[86.9 - 91.2]	81.9	[78.5 - 84.8]	0.19
**'LcJVV01.fna'**	**'LcRL03.fna'**	67.4	[63.6 - 71.1]	89.2	[86.8 - 91.2]	73.3	[69.8 - 76.5]	0.04
**'LcAB70.fna'**	**'LcMV3AUS.fna'**	79.7	[75.7 - 83.1]	89.2	[86.8 - 91.2]	84.2	[80.9 - 87.0]	0.54
**'LcCRI10.fasta'**	**'LcRL10.fna'**	87.9	[84.4 - 90.7]	89.1	[86.7 - 91.1]	90.8	[88.1 - 92.9]	0.04
**'LcCRI8.fasta'**	**'LcJVV01.fna'**	75.6	[71.7 - 79.2]	89.1	[86.7 - 91.1]	80.7	[77.3 - 83.7]	0.08
**'LcRL03.fna'**	**'LcRL09.fna'**	80.8	[76.9 - 84.2]	89.1	[86.7 - 91.1]	85.1	[82.0 - 87.8]	0.01
**'LcCRI8.fasta'**	**'LcMV3AUS.fna'**	77.6	[73.7 - 81.2]	89.1	[86.7 - 91.1]	82.4	[79.1 - 85.3]	0.25
**'LcCRI17.fasta'**	**'LcRL09.fna'**	81.7	[77.8 - 85.0]	89.1	[86.7 - 91.1]	85.8	[82.7 - 88.5]	0.01
**'LcCRI17.fasta'**	**'LcRL10.fna'**	82.7	[78.9 - 86.0]	88.9	[86.5 - 91.0]	86.7	[83.6 - 89.2]	0.17
**'Lc2029.fna'**	**'LcCRI4.fasta'**	87.3	[83.7 - 90.1]	88.9	[86.5 - 90.9]	90.3	[87.6 - 92.5]	0.17
**'Lc1252CHN.fna'**	**'LcRL07.fna'**	69.8	[65.8 - 73.4]	88.9	[86.5 - 91.0]	75.4	[71.9 - 78.6]	0.31
**'LcCRI10.fasta'**	**'LcMV3AUS.fna'**	81.2	[77.3 - 84.6]	88.8	[86.4 - 90.9]	85.4	[82.2 - 88.1]	0.32
**'LcMV1AUS.fna'**	**'LcRL05.fna'**	64.8	[61.0 - 68.5]	88.8	[86.4 - 90.9]	70.7	[67.3 - 74.0]	0.35

Query	Subject	d_0	C.I. d_0	d_4	C.I. d_4	d_6	C.I. d_6	Diff. G+C Percent
**'LcRL08.fna'**	**'LcRL10.fna'**	89.0	[85.7 - 91.7]	88.8	[86.4 - 90.9]	91.7	[89.1 - 93.6]	0.03
**'LcCRI4.fasta'**	**'LcCRI8.fasta'**	84.4	[80.6 - 87.5]	88.8	[86.3 - 90.8]	88.0	[85.1 - 90.5]	0.04
**'LcRL02.fna'**	**'LcRL08.fna'**	86.5	[82.8 - 89.4]	88.8	[86.4 - 90.9]	89.7	[86.9 - 91.9]	0.13
**'Lc1252CHN.fna'**	**'LcRL06.fna'**	69.4	[65.5 - 73.0]	88.7	[86.3 - 90.8]	75.0	[71.5 - 78.2]	0.3
**'LcMV1AUS.fna'**	**'LcRL03.fna'**	72.5	[68.5 - 76.1]	88.6	[86.1 - 90.6]	77.8	[74.3 - 80.9]	0.06
**'LcRL08.fna'**	**'LcRL09.fna'**	87.4	[83.8 - 90.2]	88.6	[86.1 - 90.6]	90.3	[87.6 - 92.5]	0.19
**'LcCRI17.fasta'**	**'LcJVV01.fna'**	74.2	[70.2 - 77.8]	88.4	[85.9 - 90.5]	79.3	[75.9 - 82.4]	0.02
**'LcRL02.fna'**	**'LcRL05.fna'**	74.0	[70.1 - 77.7]	88.4	[86.0 - 90.5]	79.2	[75.8 - 82.2]	0.45
**'LcRL05.fna'**	**'LcRL10.fna'**	72.7	[68.7 - 76.3]	88.4	[86.0 - 90.5]	77.9	[74.5 - 81.0]	0.56
**'LcCTV05.fna'**	**'LcRL02.fna'**	75.0	[71.0 - 78.6]	88.4	[86.0 - 90.5]	80.0	[76.6 - 83.0]	0.09
**'LcRL05.fna'**	**'LcRL09.fna'**	74.8	[70.8 - 78.4]	88.3	[85.9 - 90.4]	79.9	[76.5 - 82.9]	0.4
**'LcCTV05.fna'**	**'LcRL09.fna'**	75.5	[71.5 - 79.0]	88.3	[85.8 - 90.4]	80.4	[77.0 - 83.4]	0.14
**'LcCRI4.fasta'**	**'LcRL07.fna'**	86.8	[83.2 - 89.7]	88.2	[85.7 - 90.3]	89.8	[87.0 - 92.0]	0.02
**'LcCRI10.fasta'**	**'LcJVV01.fna'**	73.9	[69.9 - 77.5]	88.2	[85.7 - 90.3]	79.0	[75.6 - 82.1]	0.15
**'LcAB70.fna'**	**'LcJVV01.fna'**	76.4	[72.4 - 79.9]	88.2	[85.7 - 90.3]	81.2	[77.8 - 84.1]	0.37
**'LcCTV05.fna'**	**'LcJVV01.fna'**	64.5	[60.7 - 68.1]	88.2	[85.7 - 90.3]	70.3	[66.8 - 73.5]	0.17
**'LcCRI4.fasta'**	**'LcRL06.fna'**	86.2	[82.6 - 89.2]	88.1	[85.6 - 90.2]	89.4	[86.5 - 91.6]	0.03
**'LcCRI4.fasta'**	**'LcCRI10.fasta'**	87.1	[83.5 - 90.0]	88.0	[85.4 - 90.1]	90.0	[87.3 - 92.2]	0.03
**'LcCTV05.fna'**	**'LcRL10.fna'**	74.2	[70.2 - 77.8]	88.0	[85.5 - 90.1]	79.3	[75.9 - 82.3]	0.02
**'Lc2029.fna'**	**'LcMV1AUS.fna'**	78.6	[74.6 - 82.1]	87.9	[85.4 - 90.1]	83.1	[79.8 - 85.9]	0.03
**'Lc1252CHN.fna'**	**'LcRL08.fna'**	71.9	[68.0 - 75.6]	87.8	[85.2 - 89.9]	77.2	[73.7 - 80.3]	0.43
**'LcMV1AUS.fna'**	**'LcRL08.fna'**	79.4	[75.5 - 82.9]	87.7	[85.1 - 89.8]	83.7	[80.5 - 86.6]	0.24
**'LcCRI4.fasta'**	**'LcRL08.fna'**	86.7	[83.1 - 89.6]	87.7	[85.2 - 89.9]	89.7	[86.9 - 91.9]	0.1
**'LcCRI4.fasta'**	**'LcRL05.fna'**	73.7	[69.7 - 77.3]	87.7	[85.2 - 89.8]	78.7	[75.3 - 81.8]	0.48
**'LcCRI4.fasta'**	**'LcCRI17.fasta'**	80.8	[76.8 - 84.2]	87.7	[85.2 - 89.9]	84.9	[81.7 - 87.6]	0.1
**'Lc1252CHN.fna'**	**'Lc2029.fna'**	71.8	[67.8 - 75.4]	87.6	[85.1 - 89.8]	77.0	[73.6 - 80.2]	0.16
**'Lc1252CHN.fna'**	**'LcRL03.fna'**	63.5	[59.7 - 67.1]	87.6	[85.1 - 89.8]	69.3	[65.8 - 72.5]	0.25

Query	Subject	d_0	C.I. d_0	d_4	C.I. d_4	d_6	C.I. d_6	Diff. G+C Percent
**'LcCRI8.fasta'**	**'LcMV1AUS.fna'**	79.8	[75.8 - 83.2]	87.5	[85.0 - 89.7]	84.0	[80.7 - 86.8]	0.1
**'LcCRI17.fasta'**	**'LcMV1AUS.fna'**	74.7	[70.7 - 78.3]	87.5	[85.0 - 89.7]	79.6	[76.2 - 82.6]	0.04
**'LcCRI4.fasta'**	**'LcRL03.fna'**	79.9	[75.9 - 83.3]	87.3	[84.7 - 89.4]	84.0	[80.8 - 86.8]	0.08
**'Lc1252CHN.fna'**	**'LcRL05.fna'**	61.9	[58.2 - 65.4]	87.1	[84.5 - 89.3]	67.6	[64.2 - 70.8]	0.15
**'LcCTV05.fna'**	**'LcMV1AUS.fna'**	64.2	[60.5 - 67.9]	86.7	[84.1 - 88.9]	69.8	[66.4 - 73.1]	0.19
**'Lc1252CHN.fna'**	**'LcCRI17.fasta'**	67.8	[63.9 - 71.4]	86.6	[84.0 - 88.9]	73.2	[69.7 - 76.4]	0.23
**'LcAB70.fna'**	**'LcMV1AUS.fna'**	78.1	[74.1 - 81.6]	86.6	[84.0 - 88.8]	82.4	[79.1 - 85.3]	0.39
**'Lc1252CHN.fna'**	**'LcCRI8.fasta'**	73.8	[69.8 - 77.4]	86.5	[83.8 - 88.7]	78.6	[75.2 - 81.7]	0.29
**'LcCRI4.fasta'**	**'LcCTV05.fna'**	75.8	[71.8 - 79.4]	86.5	[83.9 - 88.8]	80.4	[77.0 - 83.4]	0.05
**'LcCTV05.fna'**	**'LcMV3AUS.fna'**	69.5	[65.6 - 73.1]	86.4	[83.7 - 88.6]	74.7	[71.3 - 77.9]	0.34
**'LcCRI10.fasta'**	**'LcMV1AUS.fna'**	78.3	[74.3 - 81.8]	86.4	[83.8 - 88.7]	82.5	[79.2 - 85.4]	0.17
**'Lc1252CHN.fna'**	**'LcCRI10.fasta'**	71.5	[67.6 - 75.2]	86.4	[83.8 - 88.7]	76.6	[73.1 - 79.7]	0.36
**'Lc1252CHN.fna'**	**'LcAB70.fna'**	76.5	[72.5 - 80.0]	84.6	[81.8 - 87.0]	80.7	[77.3 - 83.7]	0.58
**'Lc1252CHN.fna'**	**'LcCTV05.fna'**	62.2	[58.4 - 65.8]	84.3	[81.5 - 86.7]	67.5	[64.1 - 70.7]	0.38
**'LcCIP104459.fna'**	*Lactobacillus crispatus* JCM 1185	80.3	[76.4 - 83.7]	81.6	[78.7 - 84.2]	83.5	[80.2 - 86.3]	0.21
**'LcCIP104459.fna'**	**'LcJVV01.fna'**	64.8	[61.0 - 68.4]	79.3	[76.4 - 82.0]	69.2	[65.8 - 72.5]	0.1
**'LcCIP104459.fna'**	**'LcRL02.fna'**	71.8	[67.8 - 75.4]	79.2	[76.2 - 81.8]	75.6	[72.2 - 78.8]	0.18
**'LcCIP104459.fna'**	**'LcMV1AUS.fna'**	65.5	[61.7 - 69.1]	79.1	[76.2 - 81.8]	69.9	[66.4 - 73.1]	0.08
**'LcCIP104459.fna'**	**'LcRL09.fna'**	71.3	[67.3 - 74.9]	79.1	[76.1 - 81.8]	75.2	[71.7 - 78.4]	0.13
**'LcCIP104459.fna'**	**'LcRL10.fna'**	69.9	[65.9 - 73.5]	78.8	[75.8 - 81.4]	73.8	[70.3 - 77.0]	0.29
**'LcCIP104459.fna'**	**'LcMV3AUS.fna'**	63.0	[59.3 - 66.6]	78.7	[75.7 - 81.4]	67.4	[64.0 - 70.7]	0.07
**'LcAB70.fna'**	**'LcCIP104459.fna'**	70.1	[66.2 - 73.7]	78.6	[75.7 - 81.3]	74.0	[70.5 - 77.2]	0.47
**'LcCIP104459.fna'**	**'LcCRI4.fasta'**	69.1	[65.2 - 72.7]	78.5	[75.6 - 81.2]	73.1	[69.6 - 76.3]	0.21
**'LcCIP104459.fna'**	**'LcRL06.fna'**	67.5	[63.6 - 71.1]	78.4	[75.5 - 81.1]	71.6	[68.1 - 74.8]	0.19
**'LcCIP104459.fna'**	**'LcRL07.fna'**	68.2	[64.3 - 71.8]	78.4	[75.4 - 81.1]	72.2	[68.7 - 75.4]	0.19
**'LcCIP104459.fna'**	**'LcRL03.fna'**	62.9	[59.2 - 66.5]	78.3	[75.3 - 81.0]	67.3	[63.9 - 70.5]	0.14
**'Lc1252CHN.fna'**	**'LcCIP104459.fna'**	63.5	[59.7 - 67.1]	78.2	[75.2 - 80.9]	67.8	[64.4 - 71.0]	0.11

Query	Subject	d_0	C.I. d_0	d_4	C.I. d_4	d_6	C.I. d_6	Diff. G+C Percent
**'LcCIP104459.fna'**	**'LcCRI17.fasta'**	65.1	[61.3 - 68.7]	78.1	[75.1 - 80.8]	69.3	[65.9 - 72.6]	0.12
**'LcCIP104459.fna'**	**'LcRL05.fna'**	59.6	[55.9 - 63.1]	78.1	[75.1 - 80.8]	64.1	[60.7 - 67.3]	0.27
**'Lc2029.fna'**	**'LcCIP104459.fna'**	67.4	[63.6 - 71.1]	78.0	[75.0 - 80.7]	71.5	[68.0 - 74.7]	0.05
**'LcCIP104459.fna'**	**'LcCRI10.fasta'**	67.1	[63.3 - 70.8]	78.0	[75.1 - 80.8]	71.2	[67.7 - 74.4]	0.25
**'LcCIP104459.fna'**	**'LcRL08.fna'**	69.3	[65.4 - 73.0]	77.8	[74.9 - 80.6]	73.2	[69.7 - 76.4]	0.31
**'LcCIP104459.fna'**	**'LcCRI8.fasta'**	70.5	[66.5 - 74.1]	77.7	[74.8 - 80.5]	74.2	[70.7 - 77.4]	0.18
**'LcRL07.fna'**	*Lactobacillus crispatus* JCM 1185	66.3	[62.4 - 69.9]	77.5	[74.6 - 80.3]	70.3	[66.9 - 73.5]	0.4
**'LcRL06.fna'**	*Lactobacillus crispatus* JCM 1185	65.8	[61.9 - 69.4]	77.2	[74.2 - 79.9]	69.8	[66.3 - 73.0]	0.4
**'LcCIP104459.fna'**	**'LcCTV05.fna'**	58.3	[54.7 - 61.8]	76.7	[73.7 - 79.5]	62.6	[59.3 - 65.8]	0.27
**'LcMV3AUS.fna'**	*Lactobacillus crispatus* JCM 1185	62.4	[58.7 - 66.0]	76.6	[73.6 - 79.4]	66.6	[63.2 - 69.8]	0.15
**'Lc2029.fna'**	*Lactobacillus crispatus* JCM 1185	65.7	[61.9 - 69.4]	76.5	[73.5 - 79.3]	69.6	[66.2 - 72.9]	0.26
**'LcRL08.fna'**	*Lactobacillus crispatus* JCM 1185	68.0	[64.1 - 71.6]	76.4	[73.4 - 79.1]	71.7	[68.2 - 74.9]	0.53
**'LcJVV01.fna'**	*Lactobacillus crispatus* JCM 1185	64.0	[60.2 - 67.6]	76.2	[73.2 - 79.0]	68.0	[64.6 - 71.2]	0.31
**'Lc1252CHN.fna'**	*Lactobacillus crispatus* JCM 1185	67.2	[63.3 - 70.8]	76.2	[73.2 - 78.9]	70.9	[67.4 - 74.1]	0.1
**'LcCRI17.fasta'**	*Lactobacillus crispatus* JCM 1185	64.3	[60.5 - 67.9]	76.1	[73.1 - 78.8]	68.2	[64.8 - 71.4]	0.33
**'LcAB70.fna'**	*Lactobacillus crispatus* JCM 1185	69.9	[66.0 - 73.5]	76.1	[73.1 - 78.9]	73.4	[69.9 - 76.6]	0.68
**'LcRL03.fna'**	*Lactobacillus crispatus* JCM 1185	59.9	[56.2 - 63.5]	76.0	[73.0 - 78.8]	64.0	[60.7 - 67.3]	0.35
**'LcRL05.fna'**	*Lactobacillus crispatus* JCM 1185	58.7	[55.1 - 62.2]	76.0	[73.0 - 78.7]	62.9	[59.6 - 66.1]	0.06
**'LcCRI8.fasta'**	*Lactobacillus crispatus* JCM 1185	69.4	[65.5 - 73.1]	75.7	[72.7 - 78.5]	72.9	[69.4 - 76.1]	0.39
**'LcCRI10.fasta'**	*Lactobacillus crispatus* JCM 1185	67.3	[63.4 - 71.0]	75.5	[72.5 - 78.3]	70.9	[67.5 - 74.2]	0.46
**'LcMV1AUS.fna'**	*Lactobacillus crispatus* JCM 1185	66.7	[62.9 - 70.4]	75.4	[72.3 - 78.1]	70.4	[66.9 - 73.6]	0.29
**'LcRL10.fna'**	*Lactobacillus crispatus* JCM 1185	68.5	[64.7 - 72.2]	75.2	[72.2 - 78.0]	72.0	[68.5 - 75.2]	0.5
**'LcRL02.fna'**	*Lactobacillus crispatus* JCM 1185	70.3	[66.4 - 73.9]	75.1	[72.1 - 77.9]	73.6	[70.1 - 76.8]	0.39
**'LcRL09.fna'**	*Lactobacillus crispatus* JCM 1185	70.8	[66.8 - 74.4]	74.9	[71.9 - 77.7]	74.0	[70.5 - 77.2]	0.34
**'LcCRI4.fasta'**	*Lactobacillus crispatus* JCM 1185	69.7	[65.8 - 73.3]	74.7	[71.7 - 77.5]	73.0	[69.5 - 76.2]	0.43
**'LcCTV05.fna'**	*Lactobacillus crispatus* JCM 1185	57.8	[54.2 - 61.3]	74.6	[71.6 - 77.4]	61.8	[58.5 - 65.0]	0.48
**'Lc2029.fna'**	*Lactobacillus gallinarum* JCM 2011	27.7	[24.3 - 31.3]	25.5	[23.2 - 28.0]	26.1	[23.2 - 29.2]	0.38

Query	Subject	d_0	C.I. d_0	d_4	C.I. d_4	d_6	C.I. d_6	Diff. G+C Percent
**'LcCTV05.fna'**	*Lactobacillus gallinarum* JCM 2011	28.3	[25.0 - 32.0]	25.1	[22.7 - 27.5]	26.5	[23.6 - 29.6]	0.6
**'LcCRI8.fasta'**	*Lactobacillus gallinarum* JCM 2011	31.8	[28.5 - 35.4]	25.1	[22.7 - 27.5]	29.2	[26.3 - 32.3]	0.5
**'LcCRI17.fasta'**	*Lactobacillus gallinarum* JCM 2011	31.5	[28.1 - 35.1]	25.1	[22.8 - 27.6]	29.0	[26.0 - 32.1]	0.44
**'LcAB70.fna'**	*Lactobacillus gallinarum* JCM 2011	32.5	[29.1 - 36.0]	25.1	[22.8 - 27.6]	29.7	[26.8 - 32.8]	0.8
**'LcCRI10.fasta'**	*Lactobacillus gallinarum* JCM 2011	31.3	[27.9 - 34.9]	25.1	[22.8 - 27.6]	28.8	[25.9 - 31.9]	0.58
**'LcRL05.fna'**	*Lactobacillus kefiranofaciens* ATCC 43761	29.5	[26.2 - 33.2]	25.0	[22.7 - 27.5]	27.4	[24.5 - 30.5]	0.68
**'LcRL05.fna'**	*Lactobacillus gallinarum* JCM 2011	29.3	[25.9 - 32.9]	25.0	[22.6 - 27.4]	27.2	[24.3 - 30.3]	0.06
**'LcRL03.fna'**	*Lactobacillus gallinarum* JCM 2011	29.2	[25.9 - 32.8]	24.9	[22.5 - 27.3]	27.2	[24.3 - 30.3]	0.46
**'Lc2029.fna'**	*Lactobacillus amylovorus* DSM 20531	28.8	[25.4 - 32.4]	24.9	[22.6 - 27.4]	26.9	[24.0 - 30.0]	0.92
**'LcCRI4.fasta'**	*Lactobacillus gallinarum* JCM 2011	32.3	[28.9 - 35.9]	24.8	[22.5 - 27.3]	29.5	[26.5 - 32.6]	0.54
**'LcRL07.fna'**	*Lactobacillus gallinarum* JCM 2011	31.4	[28.0 - 35.0]	24.8	[22.4 - 27.2]	28.8	[25.9 - 31.9]	0.52
**'LcRL06.fna'**	*Lactobacillus gallinarum* JCM 2011	31.3	[27.9 - 34.8]	24.8	[22.5 - 27.3]	28.7	[25.8 - 31.8]	0.52
**'Lc2029.fna'**	*Lactobacillus suntoryeus* LMG 22464	27.6	[24.2 - 31.2]	24.8	[22.4 - 27.2]	25.8	[23.0 - 29.0]	0.33
**'Lc2029.fna'**	*Lactobacillus sobrius* DSM 16698	28.3	[24.9 - 31.9]	24.7	[22.4 - 27.2]	26.4	[23.5 - 29.5]	0.98
**'LcRL08.fna'**	*Lactobacillus gallinarum* JCM 2011	31.0	[27.7 - 34.6]	24.6	[22.3 - 27.1]	28.5	[25.6 - 31.6]	0.64
**'LcCRI4.fasta'**	*Lactobacillus amylovorus* DSM 20531	32.2	[28.8 - 35.7]	24.6	[22.3 - 27.1]	29.3	[26.4 - 32.4]	0.76
**'Lc2029.fna'**	*Lactobacillus kitasatonis* JCM 1039	29.0	[25.6 - 32.6]	24.6	[22.3 - 27.1]	26.9	[24.0 - 30.0]	0.66
**'LcCRI17.fasta'**	*Lactobacillus kefiranofaciens* ATCC 43761	30.6	[27.3 - 34.2]	24.6	[22.3 - 27.1]	28.2	[25.3 - 31.3]	0.3
**'LcRL02.fna'**	*Lactobacillus amylovorus* DSM 20531	31.3	[27.9 - 34.9]	24.5	[22.2 - 27.0]	28.7	[25.7 - 31.8]	0.79
**'LcCRI17.fasta'**	*Lactobacillus suntoryeus* LMG 22464	29.7	[26.3 - 33.3]	24.5	[22.2 - 27.0]	27.4	[24.5 - 30.5]	0.39
**'LcRL07.fna'**	*Lactobacillus suntoryeus* LMG 22464	29.4	[26.0 - 33.0]	24.5	[22.2 - 26.9]	27.2	[24.3 - 30.3]	0.47
**'LcRL09.fna'**	*Lactobacillus gallinarum* JCM 2011	32.3	[28.9 - 35.9]	24.5	[22.2 - 27.0]	29.4	[26.5 - 32.5]	0.46
**'LcCRI10.fasta'**	*Lactobacillus suntoryeus* LMG 22464	29.3	[25.9 - 32.9]	24.5	[22.2 - 27.0]	27.1	[24.2 - 30.2]	0.52
**'LcRL10.fna'**	*Lactobacillus gallinarum* JCM 2011	31.4	[28.1 - 35.0]	24.5	[22.2 - 27.0]	28.7	[25.8 - 31.9]	0.61
**'LcRL05.fna'**	*Lactobacillus suntoryeus* LMG 22464	27.5	[24.1 - 31.1]	24.5	[22.2 - 27.0]	25.7	[22.9 - 28.8]	0.01
**'LcCRI8.fasta'**	*Lactobacillus suntoryeus* LMG 22464	29.8	[26.4 - 33.4]	24.5	[22.2 - 27.0]	27.5	[24.6 - 30.6]	0.45
**'LcCRI10.fasta'**	*Lactobacillus amylovorus* DSM 20531	32.3	[29.0 - 35.9]	24.5	[22.2 - 27.0]	29.4	[26.5 - 32.5]	0.73

Query	Subject	d_0	C.I. d_0	d_4	C.I. d_4	d_6	C.I. d_6	Diff. G+C Percent
**'LcRL02.fna'**	*Lactobacillus gallinarum* JCM 2011	32.3	[28.9 - 35.9]	24.5	[22.2 - 27.0]	29.4	[26.5 - 32.5]	0.51
**'LcRL09.fna'**	*Lactobacillus amylovorus* DSM 20531	31.6	[28.2 - 35.2]	24.5	[22.2 - 27.0]	28.9	[25.9 - 32.0]	0.85
**'LcRL10.fna'**	*Lactobacillus amylovorus* DSM 20531	31.0	[27.7 - 34.6]	24.5	[22.2 - 27.0]	28.4	[25.5 - 31.6]	0.69
**'LcAB70.fna'**	*Lactobacillus suntoryeus* LMG 22464	30.1	[26.7 - 33.7]	24.5	[22.2 - 27.0]	27.7	[24.8 - 30.9]	0.75
**'LcCRI17.fasta'**	*Lactobacillus amylovorus* DSM 20531	32.5	[29.1 - 36.0]	24.4	[22.1 - 26.8]	29.5	[26.5 - 32.6]	0.86
**'LcCTV05.fna'**	*Lactobacillus suntoryeus* LMG 22464	26.3	[23.0 - 29.9]	24.4	[22.0 - 26.8]	24.8	[21.9 - 27.9]	0.55
**'LcRL06.fna'**	*Lactobacillus amylovorus* DSM 20531	31.8	[28.4 - 35.4]	24.4	[22.1 - 26.8]	29.0	[26.0 - 32.1]	0.78
**'LcRL06.fna'**	*Lactobacillus suntoryeus* LMG 22464	29.8	[26.4 - 33.4]	24.4	[22.1 - 26.9]	27.5	[24.6 - 30.6]	0.47
**'LcRL07.fna'**	*Lactobacillus amylovorus* DSM 20531	31.2	[27.9 - 34.8]	24.4	[22.1 - 26.9]	28.6	[25.7 - 31.7]	0.78
**'LcRL05.fna'**	*Lactobacillus sobrius* DSM 16698	28.9	[25.5 - 32.5]	24.3	[22.0 - 26.8]	26.8	[23.9 - 29.9]	1.3
**'LcRL05.fna'**	*Lactobacillus amylovorus* DSM 20531	29.1	[25.8 - 32.8]	24.3	[22.0 - 26.8]	27.0	[24.1 - 30.1]	1.24
**'Lc2029.fna'**	*Lactobacillus helveticus* DSM 20075	25.5	[22.2 - 29.2]	24.3	[22.0 - 26.8]	24.2	[21.3 - 27.3]	0.09
**'LcRL10.fna'**	*Lactobacillus helveticus* DSM 20075	26.2	[22.9 - 29.9]	24.3	[22.0 - 26.8]	24.7	[21.9 - 27.8]	0.33
**'LcCRI4.fasta'**	*Lactobacillus kitasatonis* JCM 1039	32.6	[29.2 - 36.1]	24.3	[22.0 - 26.8]	29.5	[26.6 - 32.6]	0.49
**'LcAB70.fna'**	*Lactobacillus amylovorus* DSM 20531	33.0	[29.6 - 36.6]	24.3	[22.0 - 26.8]	29.9	[26.9 - 33.0]	0.5
**'LcRL05.fna'**	*Lactobacillus kitasatonis* JCM 1039	28.6	[25.2 - 32.2]	24.3	[22.0 - 26.8]	26.6	[23.7 - 29.7]	0.97
**'LcRL08.fna'**	*Lactobacillus suntoryeus* LMG 22464	29.7	[26.4 - 33.4]	24.3	[21.9 - 26.7]	27.4	[24.5 - 30.5]	0.59
**'LcCRI8.fasta'**	*Lactobacillus amylovorus* DSM 20531	33.0	[29.6 - 36.5]	24.3	[22.0 - 26.8]	29.8	[26.9 - 32.9]	0.8
**'LcRL03.fna'**	*Lactobacillus suntoryeus* LMG 22464	27.7	[24.3 - 31.3]	24.3	[22.0 - 26.8]	25.9	[23.0 - 29.0]	0.41
**'LcCRI4.fasta'**	*Lactobacillus suntoryeus* LMG 22464	30.2	[26.8 - 33.8]	24.3	[22.0 - 26.8]	27.7	[24.8 - 30.9]	0.49
**'LcCRI10.fasta'**	*Lactobacillus sobrius* DSM 16698	31.6	[28.2 - 35.2]	24.3	[22.0 - 26.7]	28.8	[25.9 - 31.9]	0.78
**'LcCRI10.fasta'**	*Lactobacillus kitasatonis* JCM 1039	30.9	[27.5 - 34.4]	24.3	[22.0 - 26.7]	28.3	[25.3 - 31.4]	0.46
**'LcRL07.fna'**	*Lactobacillus sobrius* DSM 16698	30.7	[27.4 - 34.3]	24.2	[21.9 - 26.6]	28.1	[25.2 - 31.2]	0.84
**'LcRL08.fna'**	*Lactobacillus amylovorus* DSM 20531	32.0	[28.6 - 35.6]	24.2	[21.9 - 26.6]	29.1	[26.1 - 32.2]	0.66
**'LcRL06.fna'**	*Lactobacillus kitasatonis* JCM 1039	31.6	[28.2 - 35.2]	24.2	[21.9 - 26.7]	28.8	[25.9 - 31.9]	0.51
**'LcRL09.fna'**	*Lactobacillus kitasatonis* JCM 1039	32.1	[28.8 - 35.7]	24.2	[21.9 - 26.7]	29.2	[26.3 - 32.3]	0.58
**'LcRL10.fna'**	*Lactobacillus sobrius* DSM 16698	31.1	[27.7 - 34.7]	24.2	[21.9 - 26.6]	28.4	[25.5 - 31.5]	0.74

Query	Subject	d_0	C.I. d_0	d_4	C.I. d_4	d_6	C.I. d_6	Diff. G+C Percent
**'LcRL02.fna'**	*Lactobacillus sobrius* DSM 16698	31.6	[28.2 - 35.2]	24.2	[21.9 - 26.7]	28.8	[25.8 - 31.9]	0.85
**'LcCRI4.fasta'**	*Lactobacillus sobrius* DSM 16698	32.0	[28.6 - 35.6]	24.2	[21.9 - 26.7]	29.1	[26.2 - 32.2]	0.81
**'LcRL02.fna'**	*Lactobacillus kitasatonis* JCM 1039	32.3	[28.9 - 35.8]	24.2	[21.9 - 26.6]	29.3	[26.3 - 32.4]	0.53
**'LcJVV01.fna'**	*Lactobacillus gallinarum* JCM 2011	31.3	[27.9 - 34.9]	24.2	[21.9 - 26.7]	28.6	[25.7 - 31.7]	0.42
**'LcRL09.fna'**	*Lactobacillus sobrius* DSM 16698	31.7	[28.4 - 35.3]	24.2	[21.9 - 26.6]	28.9	[26.0 - 32.0]	0.9
**'LcMV1AUS.fna'**	*Lactobacillus gallinarum* JCM 2011	30.2	[26.9 - 33.8]	24.2	[21.9 - 26.7]	27.8	[24.9 - 30.9]	0.4
**'LcRL06.fna'**	*Lactobacillus sobrius* DSM 16698	31.2	[27.8 - 34.8]	24.2	[21.9 - 26.7]	28.5	[25.6 - 31.6]	0.84
**'LcRL09.fna'**	*Lactobacillus suntoryeus* LMG 22464	30.0	[26.7 - 33.6]	24.2	[21.9 - 26.6]	27.6	[24.7 - 30.7]	0.4
**'LcRL03.fna'**	*Lactobacillus amylovorus* DSM 20531	29.7	[26.4 - 33.3]	24.2	[21.9 - 26.7]	27.4	[24.5 - 30.5]	0.84
**'LcAB70.fna'**	*Lactobacillus sobrius* DSM 16698	32.7	[29.3 - 36.2]	24.2	[21.9 - 26.7]	29.6	[26.6 - 32.7]	0.56
**'LcRL10.fna'**	*Lactobacillus suntoryeus* LMG 22464	29.2	[25.8 - 32.8]	24.2	[21.9 - 26.7]	27.0	[24.1 - 30.1]	0.56
**'LcRL02.fna'**	*Lactobacillus suntoryeus* LMG 22464	30.0	[26.7 - 33.6]	24.2	[21.8 - 26.6]	27.6	[24.7 - 30.7]	0.46
**'LcRL07.fna'**	*Lactobacillus helveticus* DSM 20075	27.1	[23.7 - 30.7]	24.2	[21.9 - 26.6]	25.4	[22.5 - 28.5]	0.24
**'LcJVV01.fna'**	*Lactobacillus amylovorus* DSM 20531	31.3	[27.9 - 34.8]	24.2	[21.9 - 26.7]	28.5	[25.6 - 31.6]	0.88
**'LcRL07.fna'**	*Lactobacillus kitasatonis* JCM 1039	31.3	[27.9 - 34.9]	24.2	[21.9 - 26.7]	28.6	[25.7 - 31.7]	0.51
**'LcCRI17.fasta'**	*Lactobacillus sobrius* DSM 16698	31.7	[28.3 - 35.3]	24.2	[21.9 - 26.7]	28.9	[25.9 - 32.0]	0.91
**'LcRL05.fna'**	*Lactobacillus helveticus* DSM 20075	25.4	[22.0 - 29.0]	24.1	[21.8 - 26.5]	24.0	[21.1 - 27.1]	0.22
**'LcAB70.fna'**	*Lactobacillus kitasatonis* JCM 1039	31.4	[28.1 - 35.0]	24.1	[21.8 - 26.5]	28.6	[25.7 - 31.7]	0.23
**'LcRL03.fna'**	*Lactobacillus helveticus* DSM 20075	25.2	[21.9 - 28.9]	24.1	[21.8 - 26.6]	23.9	[21.0 - 27.0]	0.18
**'LcMV3AUS.fna'**	*Lactobacillus kitasatonis* JCM 1039	30.7	[27.3 - 34.3]	24.1	[21.8 - 26.5]	28.1	[25.2 - 31.2]	0.77
**'Lc1252CHN.fna'**	*Lactobacillus amylovorus* DSM 20531	31.6	[28.2 - 35.2]	24.1	[21.8 - 26.6]	28.7	[25.8 - 31.8]	1.09
**'LcCRI17.fasta'**	*Lactobacillus kitasatonis* JCM 1039	30.8	[27.4 - 34.4]	24.1	[21.8 - 26.6]	28.2	[25.3 - 31.3]	0.59
**'LcRL03.fna'**	*Lactobacillus kitasatonis* JCM 1039	29.5	[26.1 - 33.1]	24.1	[21.8 - 26.6]	27.2	[24.3 - 30.3]	0.57
**'LcRL03.fna'**	*Lactobacillus sobrius* DSM 16698	29.1	[25.7 - 32.7]	24.1	[21.8 - 26.5]	26.9	[24.0 - 30.0]	0.89
**'LcRL09.fna'**	*Lactobacillus helveticus* DSM 20075	27.0	[23.7 - 30.6]	24.1	[21.8 - 26.5]	25.3	[22.4 - 28.4]	0.17
**'LcCRI8.fasta'**	*Lactobacillus helveticus* DSM 20075	27.1	[23.7 - 30.7]	24.1	[21.8 - 26.5]	25.3	[22.5 - 28.4]	0.22
**'LcCRI8.fasta'**	*Lactobacillus kitasatonis* JCM 1039	31.5	[28.1 - 35.0]	24.1	[21.8 - 26.6]	28.7	[25.7 - 31.8]	0.53

Query	Subject	d_0	C.I. d_0	d_4	C.I. d_4	d_6	C.I. d_6	Diff. G+C Percent
**'LcJVV01.fna'**	*Lactobacillus kitasatonis* JCM 1039	32.4	[29.0 - 35.9]	24.1	[21.8 - 26.6]	29.3	[26.4 - 32.4]	0.61
**'Lc1252CHN.fna'**	*Lactobacillus gallinarum* JCM 2011	30.9	[27.5 - 34.5]	24.1	[21.8 - 26.6]	28.2	[25.3 - 31.4]	0.21
**'Lc1252CHN.fna'**	*Lactobacillus kitasatonis* JCM 1039	32.2	[28.9 - 35.8]	24.1	[21.8 - 26.6]	29.2	[26.3 - 32.3]	0.82
**'LcRL10.fna'**	*Lactobacillus kitasatonis* JCM 1039	31.9	[28.5 - 35.4]	24.1	[21.8 - 26.6]	29.0	[26.0 - 32.1]	0.42
**'LcCIP104459.fna'**	*Lactobacillus gallinarum* JCM 2011	35.3	[31.9 - 38.8]	24.1	[21.8 - 26.6]	31.4	[28.5 - 34.5]	0.33
**'LcCRI8.fasta'**	*Lactobacillus sobrius* DSM 16698	32.3	[28.9 - 35.9]	24.1	[21.8 - 26.6]	29.3	[26.4 - 32.4]	0.85
**'LcMV1AUS.fna'**	*Lactobacillus amylovorus* DSM 20531	30.3	[26.9 - 33.9]	24.1	[21.8 - 26.6]	27.8	[24.9 - 30.9]	0.9
**'LcCTV05.fna'**	*Lactobacillus amylovorus* DSM 20531	29.7	[26.3 - 33.3]	24.0	[21.7 - 26.5]	27.3	[24.4 - 30.4]	0.7
**'LcRL02.fna'**	*Lactobacillus helveticus* DSM 20075	26.8	[23.4 - 30.4]	24.0	[21.7 - 26.5]	25.1	[22.2 - 28.2]	0.22
**'LcRL06.fna'**	*Lactobacillus helveticus* DSM 20075	27.6	[24.3 - 31.3]	24.0	[21.7 - 26.5]	25.8	[22.9 - 28.9]	0.23
**'LcMV3AUS.fna'**	*Lactobacillus gallinarum* JCM 2011	30.6	[27.2 - 34.2]	24.0	[21.7 - 26.5]	28.0	[25.1 - 31.1]	0.26
**'LcMV1AUS.fna'**	*Lactobacillus kitasatonis* JCM 1039	31.7	[28.3 - 35.3]	24.0	[21.7 - 26.5]	28.8	[25.9 - 31.9]	0.63
**'LcMV3AUS.fna'**	*Lactobacillus suntoryeus* LMG 22464	28.3	[25.0 - 31.9]	24.0	[21.7 - 26.4]	26.3	[23.4 - 29.4]	0.21
**'LcAB70.fna'**	*Lactobacillus helveticus* DSM 20075	26.8	[23.4 - 30.4]	24.0	[21.7 - 26.5]	25.1	[22.2 - 28.2]	0.51
**'LcMV3AUS.fna'**	*Lactobacillus amylovorus* DSM 20531	30.3	[27.0 - 33.9]	24.0	[21.7 - 26.5]	27.8	[24.9 - 30.9]	1.04
**'LcCIP104459.fna'**	*Lactobacillus suntoryeus* LMG 22464	31.4	[28.0 - 35.0]	24.0	[21.6 - 26.4]	28.6	[25.7 - 31.7]	0.28
**'LcMV1AUS.fna'**	*Lactobacillus suntoryeus* LMG 22464	28.8	[25.4 - 32.4]	24.0	[21.7 - 26.5]	26.6	[23.7 - 29.7]	0.35
**'LcCRI17.fasta'**	*Lactobacillus helveticus* DSM 20075	27.0	[23.6 - 30.6]	24.0	[21.7 - 26.4]	25.2	[22.4 - 28.3]	0.16
**'LcRL08.fna'**	*Lactobacillus kitasatonis* JCM 1039	31.8	[28.4 - 35.4]	24.0	[21.7 - 26.5]	28.9	[26.0 - 32.0]	0.39
**'LcJVV01.fna'**	*Lactobacillus suntoryeus* LMG 22464	29.5	[26.1 - 33.1]	24.0	[21.7 - 26.4]	27.1	[24.2 - 30.2]	0.37
**'LcCRI4.fasta'**	*Lactobacillus helveticus* DSM 20075	27.0	[23.6 - 30.6]	24.0	[21.7 - 26.5]	25.3	[22.4 - 28.4]	0.26
**'LcRL08.fna'**	*Lactobacillus helveticus* DSM 20075	26.9	[23.5 - 30.5]	24.0	[21.7 - 26.5]	25.2	[22.3 - 28.3]	0.36
**'LcCRI10.fasta'**	*Lactobacillus helveticus* DSM 20075	26.7	[23.4 - 30.4]	24.0	[21.7 - 26.5]	25.0	[22.2 - 28.2]	0.29
**'LcRL08.fna'**	*Lactobacillus sobrius* DSM 16698	31.3	[27.9 - 34.9]	24.0	[21.7 - 26.5]	28.5	[25.6 - 31.6]	0.71
**'Lc1252CHN.fna'**	*Lactobacillus suntoryeus* LMG 22464	29.1	[25.7 - 32.7]	23.9	[21.6 - 26.4]	26.8	[23.9 - 29.9]	0.16
**'LcMV1AUS.fna'**	*Lactobacillus helveticus* DSM 20075	26.0	[22.7 - 29.6]	23.9	[21.6 - 26.4]	24.5	[21.6 - 27.6]	0.12
**'LcJVV01.fna'**	*Lactobacillus helveticus* DSM 20075	26.5	[23.2 - 30.2]	23.9	[21.6 - 26.4]	24.9	[22.0 - 28.0]	0.14

Query	Subject	d_0	C.I. d_0	d_4	C.I. d_4	d_6	C.I. d_6	Diff. G+C Percent
**'LcMV3AUS.fna'**	*Lactobacillus helveticus* DSM 20075	25.9	[22.6 - 29.6]	23.9	[21.6 - 26.3]	24.4	[21.6 - 27.5]	0.02
**'LcJVV01.fna'**	*Lactobacillus sobrius* DSM 16698	30.9	[27.5 - 34.5]	23.9	[21.6 - 26.3]	28.2	[25.2 - 31.3]	0.93
**'Lc1252CHN.fna'**	*Lactobacillus helveticus* DSM 20075	26.3	[23.0 - 30.0]	23.9	[21.6 - 26.4]	24.7	[21.9 - 27.8]	0.07
**'LcCIP104459.fna'**	*Lactobacillus helveticus* DSM 20075	28.1	[24.7 - 31.7]	23.9	[21.6 - 26.4]	26.1	[23.2 - 29.2]	0.05
**'Lc2029.fna'**	*Lactobacillus kefiranofaciens* ATCC 43761	26.0	[22.7 - 29.7]	23.8	[21.5 - 26.2]	24.5	[21.6 - 27.6]	0.36
**'LcCTV05.fna'**	*Lactobacillus kitasatonis* JCM 1039	27.0	[23.7 - 30.7]	23.8	[21.5 - 26.2]	25.3	[22.4 - 28.4]	0.43
**'LcCTV05.fna'**	*Lactobacillus sobrius* DSM 16698	28.7	[25.3 - 32.3]	23.8	[21.5 - 26.3]	26.5	[23.7 - 29.7]	0.76
**'LcRL07.fna'**	*Lactobacillus kefiranofaciens* ATCC 43761	27.5	[24.1 - 31.1]	23.7	[21.4 - 26.2]	25.6	[22.7 - 28.7]	0.22
**'LcMV1AUS.fna'**	*Lactobacillus sobrius* DSM 16698	30.3	[26.9 - 33.9]	23.7	[21.4 - 26.2]	27.7	[24.8 - 30.8]	0.95
**'Lc1252CHN.fna'**	*Lactobacillus sobrius* DSM 16698	31.0	[27.6 - 34.6]	23.7	[21.4 - 26.2]	28.2	[25.3 - 31.3]	1.14
**'LcCTV05.fna'**	*Lactobacillus helveticus* DSM 20075	24.5	[21.2 - 28.1]	23.7	[21.4 - 26.2]	23.2	[20.4 - 26.3]	0.31
**'LcMV3AUS.fna'**	*Lactobacillus sobrius* DSM 16698	30.2	[26.8 - 33.8]	23.7	[21.4 - 26.2]	27.6	[24.7 - 30.7]	1.1
**'LcCRI10.fasta'**	*Lactobacillus kefiranofaciens* ATCC 43761	27.9	[24.6 - 31.6]	23.6	[21.3 - 26.1]	25.9	[23.0 - 29.0]	0.17
**'Lc2029.fna'**	*Lactobacillus ultunensis* DSM 16047	25.9	[22.6 - 29.6]	23.6	[21.3 - 26.1]	24.4	[21.5 - 27.5]	0.92
**'Lc2029.fna'**	*Lactobacillus acidophilus* NBRC 13951	25.8	[22.5 - 29.5]	23.6	[21.3 - 26.1]	24.3	[21.4 - 27.4]	2.25
**'Lc2029.fna'**	*Lactobacillus kefiranofaciens* subsp. *kefirgranum* JCM 8572	26.3	[22.9 - 29.9]	23.6	[21.3 - 26.1]	24.6	[21.8 - 27.7]	0.57
**'LcRL07.fna'**	*Lactobacillus kefiranofaciens* subsp. *kefirgranum* JCM 8572	27.8	[24.4 - 31.4]	23.6	[21.3 - 26.1]	25.8	[22.9 - 28.9]	0.43
**'LcCIP104459.fna'**	*Lactobacillus sobrius* DSM 16698	32.8	[29.4 - 36.4]	23.6	[21.3 - 26.1]	29.5	[26.6 - 32.6]	1.03
**'LcCRI17.fasta'**	*Lactobacillus kefiranofaciens* subsp. *kefirgranum* JCM 8572	28.1	[24.7 - 31.7]	23.6	[21.3 - 26.0]	26.0	[23.1 - 29.1]	0.5
**'LcCIP104459.fna'**	*Lactobacillus amylovorus* DSM 20531	33.5	[30.1 - 37.1]	23.6	[21.3 - 26.1]	30.0	[27.1 - 33.1]	0.97
**'LcCRI8.fasta'**	*Lactobacillus kefiranofaciens* ATCC 43761	28.6	[25.3 - 32.3]	23.6	[21.3 - 26.0]	26.4	[23.5 - 29.5]	0.24
**'LcCTV05.fna'**	*Lactobacillus kefiranofaciens* subsp. *kefirgranum* JCM 8572	24.4	[21.1 - 28.1]	23.5	[21.2 - 25.9]	23.2	[20.3 - 26.3]	0.35
**'LcCIP104459.fna'**	*Lactobacillus kitasatonis* JCM 1039	35.6	[32.2 - 39.1]	23.5	[21.2 - 26.0]	31.5	[28.5 - 34.5]	0.7
**'LcRL05.fna'**	*Lactobacillus ultunensis* DSM 16047	26.4	[23.1 - 30.1]	23.5	[21.2 - 25.9]	24.7	[21.9 - 27.8]	0.6

Query	Subject	d_0	C.I. d_0	d_4	C.I. d_4	d_6	C.I. d_6	Diff. G+C Percent
**'LcRL03.fna'**	*Lactobacillus kefiranofaciens* ATCC 43761	26.1	[22.8 - 29.8]	23.5	[21.2 - 26.0]	24.5	[21.6 - 27.6]	0.28
**'LcRL05.fna'**	*Lactobacillus kefiranofaciens* subsp. *kefirgranum* JCM 8572	26.9	[23.6 - 30.6]	23.5	[21.2 - 25.9]	25.1	[22.2 - 28.2]	0.89
**'LcCRI10.fasta'**	*Lactobacillus kefiranofaciens* subsp. *kefirgranum* JCM 8572	27.6	[24.3 - 31.3]	23.5	[21.2 - 26.0]	25.7	[22.8 - 28.8]	0.37
**'LcAB70.fna'**	*Lactobacillus kefiranofaciens* ATCC 43761	28.8	[25.5 - 32.4]	23.5	[21.2 - 26.0]	26.6	[23.7 - 29.7]	0.06
**'LcRL07.fna'**	*Lactobacillus ultunensis* DSM 16047	28.1	[24.7 - 31.7]	23.5	[21.2 - 26.0]	26.0	[23.1 - 29.1]	1.06
**'LcCTV05.fna'**	*Lactobacillus kefiranofaciens* ATCC 43761	25.1	[21.8 - 28.8]	23.5	[21.2 - 26.0]	23.7	[20.9 - 26.8]	0.14
**'LcRL06.fna'**	*Lactobacillus kefiranofaciens* ATCC 43761	27.8	[24.4 - 31.4]	23.5	[21.2 - 26.0]	25.8	[22.9 - 28.9]	0.22
**'LcRL08.fna'**	*Lactobacillus kefiranofaciens* ATCC 43761	27.8	[24.4 - 31.4]	23.4	[21.1 - 25.8]	25.7	[22.8 - 28.8]	0.1
**'LcRL02.fna'**	*Lactobacillus ultunensis* DSM 16047	28.4	[25.1 - 32.1]	23.4	[21.1 - 25.8]	26.2	[23.3 - 29.3]	1.05
**'LcAB70.fna'**	*Lactobacillus acidophilus* NBRC 13951	28.8	[25.5 - 32.4]	23.4	[21.1 - 25.9]	26.5	[23.6 - 29.6]	2.68
**'LcRL06.fna'**	*Lactobacillus kefiranofaciens* subsp. *kefirgranum* JCM 8572	28.1	[24.7 - 31.7]	23.4	[21.1 - 25.9]	26.0	[23.1 - 29.1]	0.43
**'LcRL07.fna'**	*Lactobacillus acidophilus* NBRC 13951	27.9	[24.5 - 31.5]	23.4	[21.2 - 25.9]	25.8	[22.9 - 28.9]	2.4
**'LcCRI4.fasta'**	*Lactobacillus acidophilus* NBRC 13951	29.4	[26.1 - 33.0]	23.4	[21.1 - 25.8]	27.0	[24.1 - 30.1]	2.42
**'LcAB70.fna'**	*Lactobacillus kefiranofaciens* subsp. *kefirgranum* JCM 8572	28.5	[25.1 - 32.1]	23.4	[21.1 - 25.9]	26.3	[23.4 - 29.4]	0.15
**'LcCRI8.fasta'**	*Lactobacillus kefiranofaciens* subsp. *kefirgranum* JCM 8572	28.5	[25.1 - 32.1]	23.4	[21.1 - 25.9]	26.3	[23.4 - 29.4]	0.44
**'LcRL03.fna'**	*Lactobacillus kefiranofaciens* subsp. *kefirgranum* JCM 8572	26.3	[23.0 - 29.9]	23.4	[21.1 - 25.8]	24.6	[21.7 - 27.7]	0.48
**'LcCRI4.fasta'**	*Lactobacillus kefiranofaciens* ATCC 43761	28.5	[25.1 - 32.1]	23.4	[21.1 - 25.9]	26.3	[23.4 - 29.4]	0.2
**'LcRL10.fna'**	*Lactobacillus acidophilus* NBRC 13951	28.5	[25.2 - 32.1]	23.4	[21.1 - 25.8]	26.3	[23.4 - 29.4]	2.49
**'LcRL09.fna'**	*Lactobacillus ultunensis* DSM 16047	28.3	[24.9 - 31.9]	23.4	[21.1 - 25.9]	26.1	[23.3 - 29.2]	1.0
**'LcRL06.fna'**	*Lactobacillus ultunensis* DSM 16047	28.8	[25.4 - 32.4]	23.4	[21.1 - 25.9]	26.5	[23.6 - 29.6]	1.06
**'LcRL10.fna'**	*Lactobacillus ultunensis* DSM 16047	27.9	[24.6 - 31.5]	23.4	[21.1 - 25.8]	25.8	[23.0 - 28.9]	1.16
**'LcRL02.fna'**	*Lactobacillus acidophilus* NBRC 13951	29.3	[25.9 - 32.9]	23.3	[21.0 - 25.8]	26.9	[24.0 - 30.0]	2.38
**'LcRL06.fna'**	*Lactobacillus acidophilus* NBRC 13951	28.7	[25.3 - 32.3]	23.3	[21.0 - 25.7]	26.4	[23.5 - 29.5]	2.4

Query	Subject	d_0	C.I. d_0	d_4	C.I. d_4	d_6	C.I. d_6	Diff. G+C Percent
**'LcJVV01.fna'**	*Lactobacillus acidophilus* NBRC 13951	29.2	[25.8 - 32.8]	23.3	[21.0 - 25.8]	26.8	[23.9 - 29.9]	2.3
**'LcRL08.fna'**	*Lactobacillus kefiranofaciens* subsp. *kefirgranum* JCM 8572	28.0	[24.6 - 31.6]	23.3	[21.0 - 25.7]	25.9	[23.0 - 29.0]	0.31
**'LcRL08.fna'**	*Lactobacillus acidophilus* NBRC 13951	28.1	[24.8 - 31.8]	23.3	[21.0 - 25.7]	26.0	[23.1 - 29.1]	2.52
**'LcRL03.fna'**	*Lactobacillus ultunensis* DSM 16047	27.1	[23.7 - 30.7]	23.3	[21.0 - 25.8]	25.2	[22.3 - 28.3]	1.01
**'LcRL02.fna'**	*Lactobacillus kefiranofaciens* ATCC 43761	27.8	[24.5 - 31.4]	23.3	[21.0 - 25.8]	25.8	[22.9 - 28.9]	0.23
**'LcCRI17.fasta'**	*Lactobacillus ultunensis* DSM 16047	28.1	[24.7 - 31.7]	23.3	[21.0 - 25.8]	25.9	[23.0 - 29.0]	0.99
**'LcCRI17.fasta'**	*Lactobacillus acidophilus* NBRC 13951	27.9	[24.6 - 31.6]	23.3	[21.0 - 25.7]	25.8	[23.0 - 29.0]	2.32
**'LcRL05.fna'**	*Lactobacillus acidophilus* NBRC 13951	26.4	[23.1 - 30.1]	23.3	[21.0 - 25.7]	24.7	[21.8 - 27.8]	1.94
**'LcMV1AUS.fna'**	*Lactobacillus acidophilus* NBRC 13951	28.8	[25.4 - 32.4]	23.3	[21.0 - 25.7]	26.5	[23.6 - 29.6]	2.28
**'LcMV3AUS.fna'**	*Lactobacillus acidophilus* NBRC 13951	27.8	[24.5 - 31.5]	23.3	[21.0 - 25.7]	25.8	[22.9 - 28.9]	2.14
**'LcCRI4.fasta'**	*Lactobacillus kefiranofaciens* subsp. *kefirgranum* JCM 8572	28.6	[25.2 - 32.2]	23.3	[21.0 - 25.8]	26.3	[23.4 - 29.4]	0.4
**'LcCRI4.fasta'**	*Lactobacillus ultunensis* DSM 16047	28.8	[25.5 - 32.4]	23.3	[21.0 - 25.8]	26.5	[23.6 - 29.6]	1.08
**'LcCRI10.fasta'**	*Lactobacillus ultunensis* DSM 16047	28.2	[24.8 - 31.8]	23.3	[21.0 - 25.7]	26.0	[23.2 - 29.2]	1.12
**'LcCIP104459.fna'**	*Lactobacillus ultunensis* DSM 16047	29.8	[26.5 - 33.4]	23.3	[21.0 - 25.8]	27.2	[24.3 - 30.4]	0.87
**'LcRL10.fna'**	*Lactobacillus kefiranofaciens* ATCC 43761	27.7	[24.3 - 31.3]	23.3	[21.0 - 25.8]	25.6	[22.8 - 28.8]	0.13
**'LcRL09.fna'**	*Lactobacillus kefiranofaciens* ATCC 43761	28.3	[24.9 - 31.9]	23.3	[21.0 - 25.8]	26.1	[23.2 - 29.2]	0.29
**'LcCRI8.fasta'**	*Lactobacillus acidophilus* NBRC 13951	28.5	[25.2 - 32.2]	23.3	[21.0 - 25.8]	26.3	[23.4 - 29.4]	2.38
**'LcCRI8.fasta'**	*Lactobacillus ultunensis* DSM 16047	28.7	[25.3 - 32.3]	23.3	[21.0 - 25.7]	26.4	[23.5 - 29.5]	1.05
**'LcCRI10.fasta'**	*Lactobacillus acidophilus* NBRC 13951	28.1	[24.8 - 31.7]	23.3	[21.1 - 25.8]	26.0	[23.1 - 29.1]	2.45
**'LcRL02.fna'**	*Lactobacillus kefiranofaciens* subsp. *kefirgranum* JCM 8572	28.1	[24.7 - 31.7]	23.2	[20.9 - 25.7]	25.9	[23.1 - 29.0]	0.44
**'LcCIP104459.fna'**	*Lactobacillus acidophilus* NBRC 13951	32.0	[28.7 - 35.6]	23.2	[20.9 - 25.6]	28.8	[25.9 - 31.9]	2.21
**'LcAB70.fna'**	*Lactobacillus ultunensis* DSM 16047	28.3	[25.0 - 31.9]	23.2	[20.9 - 25.7]	26.1	[23.2 - 29.2]	1.34
**'LcJVV01.fna'**	*Lactobacillus ultunensis* DSM 16047	28.5	[25.1 - 32.1]	23.2	[20.9 - 25.7]	26.2	[23.4 - 29.4]	0.97
**'LcRL10.fna'**	*Lactobacillus kefiranofaciens* subsp. *kefirgranum* JCM 8572	27.6	[24.3 - 31.2]	23.2	[20.9 - 25.7]	25.6	[22.7 - 28.7]	0.33
**'LcRL03.fna'**	*Lactobacillus acidophilus* NBRC 13951	26.9	[23.5 - 30.5]	23.2	[20.9 - 25.7]	25.0	[22.2 - 28.1]	2.34

Query	Subject	d_0	C.I. d_0	d_4	C.I. d_4	d_6	C.I. d_6	Diff. G+C Percent
**'LcRL09.fna'**	*Lactobacillus kefiranofaciens* subsp. *kefirgranum* JCM 8572	28.4	[25.0 - 32.0]	23.2	[20.9 - 25.7]	26.1	[23.2 - 29.2]	0.49
**'LcRL08.fna'**	*Lactobacillus ultunensis* DSM 16047	28.5	[25.2 - 32.2]	23.2	[20.9 - 25.7]	26.3	[23.4 - 29.4]	1.18
**'LcRL09.fna'**	*Lactobacillus acidophilus* NBRC 13951	29.5	[26.1 - 33.1]	23.2	[20.9 - 25.7]	27.0	[24.1 - 30.1]	2.33
**'LcCTV05.fna'**	*Lactobacillus ultunensis* DSM 16047	25.1	[21.7 - 28.7]	23.2	[20.9 - 25.7]	23.6	[20.8 - 26.7]	1.14
**'Lc1252CHN.fna'**	*Lactobacillus ultunensis* DSM 16047	29.1	[25.7 - 32.7]	23.1	[20.8 - 25.6]	26.6	[23.8 - 29.8]	0.76
**'LcMV3AUS.fna'**	*Lactobacillus kefiranofaciens* ATCC 43761	26.3	[23.0 - 30.0]	23.1	[20.8 - 25.5]	24.6	[21.7 - 27.7]	0.48
**'LcMV1AUS.fna'**	*Lactobacillus kefiranofaciens* ATCC 43761	26.6	[23.2 - 30.2]	23.1	[20.8 - 25.6]	24.8	[21.9 - 27.9]	0.34
**'LcJVV01.fna'**	*Lactobacillus kefiranofaciens* subsp. *kefirgranum* JCM 8572	27.6	[24.3 - 31.2]	23.1	[20.8 - 25.6]	25.6	[22.7 - 28.7]	0.52
**'LcMV1AUS.fna'**	*Lactobacillus kefiranofaciens* subsp. *kefirgranum* JCM 8572	27.1	[23.8 - 30.8]	23.1	[20.8 - 25.5]	25.2	[22.3 - 28.3]	0.54
**'LcMV1AUS.fna'**	*Lactobacillus ultunensis* DSM 16047	28.0	[24.7 - 31.7]	23.1	[20.8 - 25.5]	25.9	[23.0 - 29.0]	0.95
**'LcJVV01.fna'**	*Lactobacillus kefiranofaciens* ATCC 43761	27.2	[23.9 - 30.9]	23.1	[20.8 - 25.6]	25.3	[22.4 - 28.4]	0.32
**'Lc1252CHN.fna'**	*Lactobacillus acidophilus* NBRC 13951	29.8	[26.4 - 33.4]	23.1	[20.8 - 25.6]	27.2	[24.3 - 30.3]	2.09
**'LcMV3AUS.fna'**	*Lactobacillus ultunensis* DSM 16047	27.7	[24.4 - 31.3]	23.0	[20.7 - 25.5]	25.6	[22.7 - 28.7]	0.8
**'LcMV3AUS.fna'**	*Lactobacillus kefiranofaciens* subsp. *kefirgranum* JCM 8572	26.9	[23.6 - 30.6]	23.0	[20.7 - 25.4]	25.0	[22.1 - 28.1]	0.69
**'Lc1252CHN.fna'**	*Lactobacillus kefiranofaciens* subsp. *kefirgranum* JCM 8572	27.6	[24.3 - 31.3]	23.0	[20.7 - 25.4]	25.6	[22.7 - 28.7]	0.73
**'Lc1252CHN.fna'**	*Lactobacillus kefiranofaciens* ATCC 43761	27.3	[23.9 - 30.9]	23.0	[20.7 - 25.4]	25.3	[22.4 - 28.4]	0.53
**'LcCTV05.fna'**	*Lactobacillus acidophilus* NBRC 13951	24.6	[21.3 - 28.3]	22.9	[20.7 - 25.4]	23.2	[20.4 - 26.3]	2.48
**'LcCIP104459.fna'**	*Lactobacillus kefiranofaciens* ATCC 43761	30.3	[27.0 - 33.9]	22.9	[20.6 - 25.3]	27.5	[24.6 - 30.6]	0.41
**'LcCIP104459.fna'**	*Lactobacillus kefiranofaciens* subsp. *kefirgranum* JCM 8572	31.1	[27.8 - 34.7]	22.8	[20.5 - 25.3]	28.1	[25.2 - 31.2]	0.62

Table 4: Strains in your dataset

Joint dataset of automatically determined closest type strains (if this mode was chosen), manually selected type strains (if selected accordingly) and the provided user strains, if provided (marked in yellow).

Strain	Authority	Other deposits	Synonyms	Base pairs	Percent G+C	No. proteins	Goldstamp	Bioproject accession	Biosample accession	Assembly accession	IMG OID
Lactobacillus suntoryeus LMG 22464	Cachat and Priest 2005	NCIMB 14005; SA	*Lactobacillus suntoryeus*	1760 061	36.5	1825	Gp0131240	PRJNA224116	SAMN02797793	GCF_001437535	
Lactobacillus kefiranofaciens ATCC 43761	Fujisawa et al. 1988 emend. Vancanneyt et al. 2004	LMG 19149; CCUG 32248; DSM 5016; JCM 6985; CIP 103307; strain WT-2B	*Lactobacillus kefiranofaciens*; *Lactobacillus kefiranofaciens* subsp. *kefiranofaciens*	2281 817	37.2	2367	Gp0099413	PRJNA257853	SAMN02983011	GCA_900103655	2597490363
Lactobacillus helveticus DSM 20075	(Orla-Jensen 1919) Bergey et al. 1925	LMG 13555; LMG 6413; NRRL B-4526; CCUG 30139; ATCC 15009; JCM 1120; IFO 15019; NBRC 15019; CIP 103146	*Lactobacillus helveticus*; *Thermobacterium helveticum*	1804 595	36.8	2078	Gp0003635	PRJNA34619	SAMN00139430	GCA_000160855	645951865
Lactobacillus ultunensis DSM 16047	Roos et al. 2005	LMG 22117; CCUG 48460; JCM 16177; Kx146C1	*Lactobacillus ultunensis*	2159 701	35.9	2210	Gp0003665	PRJNA31505	SAMN00001484	GCA_000159415	643886047

Strain	Authority	Other deposits	Synonyms	Base pairs	Percent G+C	No. proteins	Goldstamp	Bioproject accession	Biosample accession	Assembly accession	IMG OID
Lactobacillus acidophilus NBRC 13951	(Moro 1900) Hansen and Mocquot 1970	LMG 13550; LMG 9433; BCRC 10695; NRRL B-4495; CCUG 5917; DSM 20079; ATCC 4356; NCTC 12980; JCM 1132; IFO 13951; VKM B-1660; CIP 76.13	*Bacillus acidophilus*; *Lactobacillus acidophilus*	1955 274	34.6	1873	Gp0075770	PRJDB1353	SAMD00046914	GCA_001591845	
Lactobacillus kitasatonis JCM 1039	Mukai et al. 2003	KCTC 3155; DSM 16761	*Lactobacillus kitasatonis*	1902 496	37.5	2050	Gp0026726	PRJDB640	SAMD00016339	GCA_000615285	2565956592
Lactobacillus gallinarum JCM 2011	Fujisawa et al. 1992	LMG 9435; CCUG 30724; DSM 10532; ATCC 33199; CIP 103611; VPI 1294	*Lactobacillus gallinarum*	1918 774	36.5	2076	Gp0091989	PRJDB621	SAMD00003603	GCA_000614735	
Lactobacillus kefiranofaciens subsp. *kefirgranum* JCM 8572	(Takizawa et al. 1994) Vancanneyt et al. 2004	LMG 15132; CCUG 39467; CCUG 49353; DSM 10550; ATCC 51647; CIP 104241; GCL 1701	*Lactobacillus kefiranofaciens* subsp. *kefirgranum*; *Lactobacillus kefirgranum*	2065 566	37.4	2918	Gp0093297	PRJDB772	SAMD00000473	GCA_001311335	
Lactobacillus crispatus JCM 1185	(Brygoo and Aladame 1953) Moore and Holdeman 1970 emend. Cato et al. 1983	LMG 9479; CCUG 30722; DSM 20584; ATCC 33820; CIP 102990; CIPP II; VPI 3199	*Eubacterium crispatum*; *Lactobacillus crispatus*	2033 995	36.6	3305	Gp0093095	PRJDB800	SAMD00000420	GCA_001311685	

Strain	Authority	Other deposits	Synonyms	Base pairs	Percent G+C	No. proteins	Goldstamp	Bioproject accession	Biosample accession	Assembly accession	IMG OID
Lactobacillus amylovorus DSM 20531	Nakamura 1981	LMG 9496; NRRL B-4540; CCUG 27201; ATCC 33620; JCM 1126; CIP 102989; NCAIM B.01458	*Lactobacillus amylovorus*	2016 257	37.8	2045	Gp0130192	PRJNA224116	SAMN02369422	GCF_001433985	
Lactobacillus sobrius DSM 16698	Konstantinov et al. 2006	1; NCCB 100067; OTU171-001	*Lactobacillus sobrius*	1992 054	37.8	1950	Gp0131222	PRJNA224116	SAMN02797775	GCF_001437365	
Lc1252CHN.fna				2305 246	36.7	2221					
Lc2029.fna				2397 671	36.8	3004					
LcAB70.fna				2367 925	37.3	2391					
LcCIP104459.fna				1993 673	36.8	2038					
LcCRI4.fasta				2376 268	37.0	2465					
LcCRI8.fasta				2330 310	37.0	2389					
LcCRI10.fasta				2418 420	37.0	2532					
LcCRI17.fasta				2384 332	36.9	2461					
LcCTV05.fna				2364 583	37.1	2425					
LcJVV01.fna				2221 719	36.9	2216					
LcMV1AUS.fna				2311 882	36.9	2339					
LcMV3AUS.fna				2437 083	36.7	2514					

Strain	Authority	Other deposits	Synonyms	Base pairs	Percent G+C	No. proteins	Goldstamp	Bioproject accession	Biosample accession	Assembly accession	IMG OID
LcRL02.fna				2243 143	37.0	2447					
LcRL03.fna				2550 531	36.9	2886					
LcRL05.fna				2577 199	36.5	2857					
LcRL06.fna				2190 141	37.0	2483					
LcRL07.fna				2183 841	37.0	2454					
LcRL08.fna				2301 145	37.1	2622					
LcRL09.fna				2276 697	36.9	2526					
LcRL10.fna				2315 512	37.1	2579					

Methods, Results and References

The genome sequence data were uploaded to the Type (Strain) Genome Server (TYGS), a free bioinformatics platform available under https://tygs.dsmz.de, for a whole genome-based taxonomic analysis [1]. The results were provided by the TYGS on 2020-05-25. In brief, the TYGS analysis was subdivided into the following steps:

Determination of closely related type strains

Determination of closest type strain genomes was done in two complementary ways: First, all user genomes were compared against all type strain genomes available in the TYGS database via the MASH algorithm, a fast approximation of intergenomic relatedness [2], and, the ten type strains with the smallest MASH distances chosen per user genome. Second, an additional set of ten closely related type strains was determined via the 16S rDNA gene sequences. These were extracted from the user genomes using RNAmmer [3] and each sequence was subsequently BLASTed [4] against the 16S rDNA gene sequence of each of the currently 11767 type strains available in the TYGS database. This was used as a proxy to find the best 50 matching type strains (according to the bitscore) for each user genome and to subsequently calculate precise distances using the Genome BLAST Distance Phylogeny approach (GBDP) under the algorithm 'coverage' and distance formula d_5 [5]. These distances were finally used to determine the 10 closest type strain genomes for each of the user genomes.

Pairwise comparison of genome sequences

All pairwise comparisons among the set of genomes were conducted using GBDP and accurate intergenomic distances inferred under the algorithm 'trimming' and distance formula d_5 [5]. 100 distance replicates were calculated each. Digital DDH values and confidence intervals were calculated using the recommended settings of the GGDC 2.1 [5].

Phylogenetic inference

The resulting intergenomic distances were used to infer a balanced minimum evolution tree with branch support via FASTME 2.1.4 including SPR postprocessing [6]. Branch support was inferred from 100 pseudo-bootstrap replicates each. The trees were rooted at the midpoint [7] and visualized with PhyD3 [8].

Type-based species and subspecies clustering

The type-based species clustering using a 70% dDDH radius around each of the 11 type strains was done as previously described [1]. The resulting groups are shown in Table 1 and 4. Subspecies clustering was done using a 79% dDDH threshold as previously introduced [9].

Results

Type-based species and subspecies clustering

The resulting species and subspecies clusters are listed in Table 4, whereas the taxonomic identification of the query strains is found in Table 1. Briefly, the clustering yielded 8 species clusters and the provided query strains were assigned to 1 of these. Moreover, user strains were located in 2 of 10 subspecies clusters.

Figure caption genome tree

Figure 1. Tree inferred with FastME 2.1.6.1 [6] from GBDP distances calculated from genome sequences. The branch lengths are scaled in terms of GBDP distance formula d_5. The numbers above branches are GBDP pseudo-bootstrap support values > 60 % from 100 replications, with an average branch support of 56.8 %. The tree was rooted at the midpoint [7].

Figure caption SSU tree

Figure 2. Tree inferred with FastME 2.1.6.1 [6] from GBDP distances calculated from 16S rDNA gene sequences. The branch lengths are scaled in terms of GBDP distance formula d_5. The numbers above branches are GBDP pseudo-bootstrap support values > 60 % from 100 replications, with an average branch support of 51.0 %. The tree was rooted at the midpoint [7].

References

[1] Meier-Kolthoff JP, Göker M. TYGS is an automated high-throughput platform for state-of-the-art genome-based taxonomy. Nat. Commun. 2019;10: 2182. DOI: 10.1038/s41467-019-10210-3

[2] Ondov BD, Treangen TJ, Melsted P, et al. Mash: Fast genome and metagenome distance estimation using MinHash. Genome Biol 2016;17: 1–14. DOI: 10.1186/s13059-016-0997-x

[3] Lagesen K, Hallin P. RNAmmer: consistent and rapid annotation of ribosomal RNA genes. Nucleic Acids Res. Oxford Univ Press; 2007;35: 3100–3108. DOI: 10.1093/nar/gkm160

[4] Camacho C, Coulouris G, Avagyan V, Ma N, Papadopoulos J, Bealer K, et al. BLAST+: architecture and applications. BMC Bioinformatics. 2009;10: 421. DOI: 10.1186/1471-2105-10-421

[5] Meier-Kolthoff JP, Auch AF, Klenk H-P, Göker M. Genome sequence-based species delimitation with confidence intervals and improved distance functions. BMC Bioinformatics. 2013;14: 60. DOI: 10.1186/1471-2105-14-60

[6] Lefort V, Desper R, Gascuel O. FastME 2.0: A comprehensive, accurate, and fast distance-based phylogeny inference program. Mol Biol Evol. 2015;32: 2798–2800. DOI: 10.1093/molbev/msv150

[7] Farris JS. Estimating phylogenetic trees from distance matrices. Am Nat. 1972;106: 645–667.

[8] Kreft L, Botzki A, Coppens F, Vandepoele K, Van Bel M. PhyD3: A phylogenetic tree viewer with extended phyloXML support for functional genomics data visualization. Bioinformatics. 2017;33: 2946–2947. DOI: 10.1093/bioinformatics/btx324

[9] Meier-Kolthoff JP, Hahnke RL, Petersen J, Scheuner C, Michael V, Fiebig A, et al. Complete genome sequence of DSM 30083^{T}, the type strain (U5/41^{T}) of *Escherichia coli*, and a proposal for delineating subspecies in microbial taxonomy. Stand Genomic Sci. 2014;9: 2. DOI: 10.1186/1944-3277-9-2

TYPE (STRAIN) GENOME SERVER

JOB SUMMARY

PRINT DATE: 2020-05-25 04:09:36 +0200

JOB ID: eb8c6992-e23b-4401-bc2d-2f9af584f64e

RESULT PAGE: https://tygs.dsmz.de/user_results/show?guid=eb8c6992-e23b-4401-bc2d-2f9af584f64e

Table 1: Phylogenies

Publication-ready versions of both the genome-scale GBDP tree and the 16S rRNA gene sequence tree can be customized and exported either in SVG (vector graphic) or PNG format from within the phylogeny viewers in your TYGS result page. For publications the **SVG format is recommended** because it is lossless, always keeps its high resolution and can also be easily converted to other popular formats such as PDF or EPS. Please follow the link provided above!

Table 2: Identification

The below list contains the result of the TYGS species identification routine.

Explanation of remarks that might occur in the below table:

remark [R1]: The TYGS type strain database is automatically updated on an almost daily basis. However, if a particular type strain genome is not available in the TYGS database, this can have several reasons which are detailed in the FAQ. You can request an extended 16S rRNA gene analysis via the 16S tree viewer found in your result page to detect **not yet genome-sequenced** type strains relevant for your study.

remark [R2]: > 70% dDDH value (formula d_4) and (almost) minimal dDDH values for gene-content formulae d_0 and d_6 indicate a potentially unreliable identification result and should thus be checked via the 16S rRNA gene sequence similarity. Such strong deviations can, in principle, be caused by sequence contamination.

remark [R3]: G+C content difference of > 1 % indicates a potentially unreliable identification result because within species G+C content varies no more than 1 %, if computed from genome sequences (PMID: 24505073).

Strain	Conclusion	Identification result	Remark
**'LcRL11'**	belongs to known species	*Lactobacillus crispatus*	
**'LcRL13'**	belongs to known species	*Lactobacillus crispatus*	
**'LcRL14'**	belongs to known species	*Lactobacillus crispatus*	
**'LcRL15'**	belongs to known species	*Lactobacillus crispatus*	
**'LcRL16'**	belongs to known species	*Lactobacillus crispatus*	
**'LcRL17'**	belongs to known species	*Lactobacillus crispatus*	see [R3]
**'LcRL19'**	belongs to known species	*Lactobacillus crispatus*	see [R3]
**'LcRL20'**	belongs to known species	*Lactobacillus crispatus*	
**'LcRL21'**	belongs to known species	*Lactobacillus crispatus*	
**'LcRL23'**	belongs to known species	*Lactobacillus crispatus*	
**'LcRL24'**	belongs to known species	*Lactobacillus crispatus*	
**'LcRL25'**	belongs to known species	*Lactobacillus crispatus*	
**'LcRL26'**	belongs to known species	*Lactobacillus crispatus*	
**'LcRL27'**	belongs to known species	*Lactobacillus crispatus*	
**'LcRL28'**	belongs to known species	*Lactobacillus crispatus*	

Strain	Conclusion	Identification result	Remark
**'LcRL29'**	belongs to known species	*Lactobacillus crispatus*	
**'LcRL30'**	belongs to known species	*Lactobacillus crispatus*	
**'LcRL31'**	belongs to known species	*Lactobacillus crispatus*	
**'LcRL32'**	belongs to known species	*Lactobacillus crispatus*	
**'LcRL33'**	belongs to known species	*Lactobacillus crispatus*	

Table 3: Pairwise comparisons of user genomes vs. type-strain genomes

The following table contains the pairwise dDDH values between your user genomes and the selected type-strain genomes. The dDDH values are provided along with their confidence intervals (C.I.) for the three different GBDP formulas:

- formula d_0 (a.k.a. GGDC formula 1): length of all HSPs divided by total genome length
- formula d_4 (a.k.a. GGDC formula 2): sum of all identities found in HSPs divided by overall HSP length
- formula d_6 (a.k.a. GGDC formula 3): sum of all identities found in HSPs divided by total genome length

Note: Formula d_4 is independent of genome length and is thus robust against the use of incomplete draft genomes. For other reasons for preferring formula d_4, see the FAQ.

Query	Subject	d_0	C.I. d_0	d_4	C.I. d_4	d_6	C.I. d_6	Diff. G+C Percent
**'LcRL21.fna'**	**'LcRL27.fna'**	98.9	[98.1 - 99.4]	99.9	[99.7 - 99.9]	99.5	[99.2 - 99.7]	0.08
**'LcRL24.fna'**	**'LcRL26.fna'**	95.0	[92.6 - 96.6]	99.9	[99.8 - 100.0]	97.3	[96.0 - 98.2]	0.68
**'LcRL19.fna'**	**'LcRL33.fna'**	92.3	[89.4 - 94.4]	99.9	[99.7 - 99.9]	95.6	[93.8 - 96.9]	0.75
**'LcRL19.fna'**	**'LcRL24.fna'**	94.4	[92.0 - 96.2]	99.9	[99.8 - 99.9]	97.0	[95.5 - 97.9]	0.92
**'LcRL24.fna'**	**'LcRL33.fna'**	99.1	[98.3 - 99.5]	99.9	[99.8 - 99.9]	99.6	[99.3 - 99.8]	0.17
**'LcRL13.fna'**	**'LcRL28.fna'**	99.4	[98.9 - 99.7]	99.9	[99.8 - 99.9]	99.8	[99.5 - 99.9]	0.14
**'LcRL31.fna'**	**'LcRL32.fna'**	99.1	[98.4 - 99.5]	99.9	[99.9 - 100.0]	99.6	[99.3 - 99.8]	0.04
**'LcRL19.fna'**	**'LcRL26.fna'**	98.1	[96.9 - 98.9]	99.9	[99.8 - 100.0]	99.1	[98.5 - 99.5]	0.24
**'LcRL26.fna'**	**'LcRL33.fna'**	92.8	[90.0 - 94.8]	99.9	[99.8 - 99.9]	95.9	[94.2 - 97.1]	0.51
**'LcRL15.fna'**	**'LcRL30.fna'**	99.4	[98.8 - 99.7]	99.8	[99.6 - 99.9]	99.7	[99.5 - 99.9]	0.25
**'LcRL26.fna'**	**'LcRL29.fna'**	98.4	[97.2 - 99.1]	99.8	[99.7 - 99.9]	99.2	[98.7 - 99.6]	0.28
**'LcRL19.fna'**	**'LcRL29.fna'**	98.0	[96.7 - 98.8]	99.8	[99.7 - 99.9]	99.0	[98.4 - 99.4]	0.52
**'LcRL29.fna'**	**'LcRL33.fna'**	93.0	[90.3 - 95.1]	99.8	[99.7 - 99.9]	96.1	[94.4 - 97.3]	0.23
**'LcRL24.fna'**	**'LcRL29.fna'**	94.7	[92.3 - 96.4]	99.8	[99.7 - 99.9]	97.1	[95.7 - 98.1]	0.4
**'LcRL23.fna'**	**'LcRL25.fna'**	99.9	[99.8 - 100.0]	99.7	[99.6 - 99.9]	100.0	[99.9 - 100.0]	0.07
**'LcRL15.fna'**	**'LcRL17.fna'**	96.6	[94.7 - 97.8]	99.6	[99.3 - 99.8]	98.2	[97.2 - 98.9]	1.18
**'LcRL11.fna'**	**'LcRL25.fna'**	95.8	[93.8 - 97.2]	99.4	[99.0 - 99.6]	97.7	[96.6 - 98.5]	0.02
**'LcRL11.fna'**	**'LcRL33.fna'**	90.4	[87.2 - 92.9]	99.4	[99.0 - 99.6]	94.2	[92.1 - 95.8]	0.03
**'LcRL23.fna'**	**'LcRL28.fna'**	94.9	[92.5 - 96.5]	99.4	[99.0 - 99.6]	97.1	[95.8 - 98.1]	0.43
**'LcRL13.fna'**	**'LcRL33.fna'**	89.9	[86.7 - 92.5]	99.4	[99.0 - 99.6]	93.9	[91.7 - 95.5]	0.22
**'LcRL25.fna'**	**'LcRL28.fna'**	94.7	[92.3 - 96.4]	99.4	[99.1 - 99.7]	97.0	[95.6 - 98.0]	0.36
**'LcRL28.fna'**	**'LcRL33.fna'**	89.4	[86.0 - 92.0]	99.4	[99.0 - 99.6]	93.5	[91.2 - 95.2]	0.36

Query	Subject	d_0	C.I. d_0	d_4	C.I. d_4	d_6	C.I. d_6	Diff. G+C Percent
**'LcRL17.fna'**	**'LcRL30.fna'**	97.4	[95.8 - 98.4]	99.4	[99.1 - 99.6]	98.7	[97.9 - 99.2]	1.43
**'LcRL11.fna'**	**'LcRL23.fna'**	96.0	[94.0 - 97.4]	99.4	[99.0 - 99.6]	97.8	[96.7 - 98.6]	0.09
**'LcRL13.fna'**	**'LcRL25.fna'**	95.1	[92.9 - 96.7]	99.3	[98.9 - 99.6]	97.3	[96.0 - 98.2]	0.22
**'LcRL19.fna'**	**'LcRL28.fna'**	92.8	[90.0 - 94.9]	99.3	[98.9 - 99.5]	95.8	[94.1 - 97.1]	0.38
**'LcRL26.fna'**	**'LcRL28.fna'**	93.4	[90.7 - 95.3]	99.3	[98.8 - 99.5]	96.2	[94.5 - 97.3]	0.15
**'LcRL13.fna'**	**'LcRL19.fna'**	93.4	[90.7 - 95.3]	99.3	[98.8 - 99.5]	96.2	[94.5 - 97.3]	0.53
**'LcRL11.fna'**	**'LcRL19.fna'**	93.8	[91.3 - 95.7]	99.3	[98.8 - 99.5]	96.5	[94.9 - 97.6]	0.72
**'LcRL11.fna'**	**'LcRL29.fna'**	94.1	[91.6 - 95.9]	99.2	[98.7 - 99.5]	96.6	[95.1 - 97.7]	0.21
**'LcRL20.fna'**	**'LcRL28.fna'**	86.8	[83.2 - 89.7]	99.2	[98.8 - 99.5]	91.6	[89.0 - 93.6]	0.74
**'LcRL28.fna'**	**'LcRL30.fna'**	96.3	[94.4 - 97.6]	99.2	[98.8 - 99.5]	98.0	[97.0 - 98.7]	0.33
**'LcRL13.fna'**	**'LcRL24.fna'**	89.0	[85.7 - 91.7]	99.2	[98.7 - 99.5]	93.2	[90.9 - 94.9]	0.39
**'LcRL11.fna'**	**'LcRL26.fna'**	94.4	[92.0 - 96.2]	99.2	[98.8 - 99.5]	96.9	[95.4 - 97.9]	0.48
**'LcRL13.fna'**	**'LcRL29.fna'**	93.6	[91.0 - 95.5]	99.2	[98.7 - 99.5]	96.3	[94.7 - 97.5]	0.01
**'LcRL13.fna'**	**'LcRL23.fna'**	95.3	[93.1 - 96.9]	99.2	[98.8 - 99.5]	97.4	[96.2 - 98.3]	0.29
**'LcRL28.fna'**	**'LcRL29.fna'**	93.1	[90.4 - 95.1]	99.2	[98.7 - 99.5]	96.0	[94.3 - 97.2]	0.13
**'LcRL11.fna'**	**'LcRL24.fna'**	89.7	[86.5 - 92.3]	99.2	[98.7 - 99.5]	93.7	[91.5 - 95.3]	0.2
**'LcRL13.fna'**	**'LcRL26.fna'**	93.9	[91.3 - 95.7]	99.2	[98.7 - 99.5]	96.5	[94.9 - 97.6]	0.29
**'LcRL17.fna'**	**'LcRL28.fna'**	92.6	[89.7 - 94.7]	99.2	[98.8 - 99.5]	95.6	[93.9 - 96.9]	1.1
**'LcRL24.fna'**	**'LcRL28.fna'**	88.5	[85.1 - 91.2]	99.2	[98.8 - 99.5]	92.8	[90.4 - 94.6]	0.53
**'LcRL15.fna'**	**'LcRL28.fna'**	94.8	[92.5 - 96.5]	99.2	[98.8 - 99.5]	97.1	[95.7 - 98.1]	0.08
**'LcRL11.fna'**	**'LcRL17.fna'**	93.5	[90.8 - 95.4]	99.2	[98.7 - 99.5]	96.3	[94.6 - 97.4]	1.44
**'LcRL11.fna'**	**'LcRL20.fna'**	88.0	[84.6 - 90.8]	99.2	[98.7 - 99.5]	92.5	[90.0 - 94.3]	0.4
**'LcRL13.fna'**	**'LcRL20.fna'**	87.5	[84.0 - 90.3]	99.2	[98.7 - 99.5]	92.0	[89.6 - 94.0]	0.6
**'LcRL11.fna'**	**'LcRL30.fna'**	97.1	[95.4 - 98.2]	99.2	[98.8 - 99.5]	98.5	[97.6 - 99.0]	0.01
**'LcRL11.fna'**	**'LcRL15.fna'**	95.9	[93.8 - 97.3]	99.2	[98.7 - 99.5]	97.8	[96.6 - 98.5]	0.26
**'LcRL11.fna'**	**'LcRL31.fna'**	92.1	[89.2 - 94.3]	99.1	[98.5 - 99.4]	95.3	[93.5 - 96.7]	0.3
**'LcRL13.fna'**	**'LcRL17.fna'**	93.1	[90.4 - 95.1]	99.1	[98.6 - 99.4]	96.0	[94.3 - 97.2]	1.24

Query	Subject	d_0	C.I. d_0	d_4	C.I. d_4	d_6	C.I. d_6	Diff. G+C Percent
**'LcRL20.fna'**	**'LcRL25.fna'**	81.3	[77.4 - 84.7]	99.1	[98.6 - 99.4]	87.2	[84.2 - 89.7]	0.38
**'LcRL28.fna'**	**'LcRL31.fna'**	91.2	[88.2 - 93.6]	99.1	[98.6 - 99.4]	94.7	[92.7 - 96.2]	0.04
**'LcRL13.fna'**	**'LcRL15.fna'**	95.3	[93.1 - 96.8]	99.1	[98.6 - 99.4]	97.4	[96.1 - 98.3]	0.06
**'LcRL28.fna'**	**'LcRL32.fna'**	90.4	[87.2 - 92.9]	99.1	[98.5 - 99.4]	94.1	[92.0 - 95.7]	0.0
**'LcRL20.fna'**	**'LcRL23.fna'**	81.6	[77.7 - 84.9]	99.1	[98.6 - 99.4]	87.4	[84.4 - 89.9]	0.31
**'LcRL13.fna'**	**'LcRL30.fna'**	96.8	[95.0 - 97.9]	99.1	[98.7 - 99.4]	98.3	[97.3 - 98.9]	0.18
**'LcRL23.fna'**	**'LcRL26.fna'**	88.9	[85.5 - 91.6]	99.0	[98.5 - 99.4]	93.1	[90.7 - 94.8]	0.58
**'LcRL11.fna'**	**'LcRL32.fna'**	91.3	[88.3 - 93.7]	99.0	[98.5 - 99.4]	94.8	[92.8 - 96.2]	0.34
**'LcRL13.fna'**	**'LcRL32.fna'**	90.9	[87.8 - 93.3]	99.0	[98.5 - 99.3]	94.5	[92.4 - 96.0]	0.14
**'LcRL19.fna'**	**'LcRL25.fna'**	88.1	[84.6 - 90.9]	99.0	[98.5 - 99.4]	92.5	[90.1 - 94.3]	0.74
**'LcRL13.fna'**	**'LcRL31.fna'**	91.8	[88.8 - 94.0]	99.0	[98.5 - 99.4]	95.1	[93.1 - 96.5]	0.1
**'LcRL25.fna'**	**'LcRL26.fna'**	88.7	[85.3 - 91.4]	99.0	[98.4 - 99.3]	92.9	[90.6 - 94.7]	0.51
**'LcRL19.fna'**	**'LcRL23.fna'**	88.3	[84.8 - 91.0]	99.0	[98.5 - 99.4]	92.6	[90.2 - 94.4]	0.81
**'LcRL23.fna'**	**'LcRL29.fna'**	88.6	[85.1 - 91.3]	98.9	[98.4 - 99.3]	92.8	[90.4 - 94.6]	0.3
**'LcRL25.fna'**	**'LcRL29.fna'**	88.3	[84.9 - 91.1]	98.9	[98.4 - 99.3]	92.6	[90.2 - 94.5]	0.23
**'LcRL11.fna'**	**'LcRL28.fna'**	99.6	[99.2 - 99.8]	98.8	[98.3 - 99.2]	99.8	[99.6 - 99.9]	0.34
**'LcRL25.fna'**	**'LcRL32.fna'**	84.8	[81.1 - 88.0]	98.7	[98.1 - 99.1]	89.9	[87.2 - 92.2]	0.36
**'LcRL23.fna'**	**'LcRL33.fna'**	84.8	[81.0 - 87.9]	98.7	[98.1 - 99.2]	89.9	[87.1 - 92.1]	0.07
**'LcRL17.fna'**	**'LcRL25.fna'**	88.2	[84.8 - 91.0]	98.7	[98.0 - 99.1]	92.5	[90.1 - 94.4]	1.46
**'LcRL17.fna'**	**'LcRL33.fna'**	86.2	[82.6 - 89.2]	98.7	[98.0 - 99.1]	91.0	[88.4 - 93.1]	1.46
**'LcRL25.fna'**	**'LcRL30.fna'**	92.3	[89.4 - 94.5]	98.7	[98.1 - 99.1]	95.4	[93.6 - 96.7]	0.03
**'LcRL23.fna'**	**'LcRL31.fna'**	85.5	[81.8 - 88.6]	98.7	[98.1 - 99.2]	90.5	[87.8 - 92.6]	0.39
**'LcRL15.fna'**	**'LcRL25.fna'**	91.0	[87.9 - 93.4]	98.7	[98.1 - 99.1]	94.5	[92.5 - 96.0]	0.28
**'LcRL25.fna'**	**'LcRL33.fna'**	84.5	[80.8 - 87.7]	98.7	[98.1 - 99.2]	89.7	[86.9 - 92.0]	0.01
**'LcRL23.fna'**	**'LcRL32.fna'**	85.0	[81.3 - 88.1]	98.7	[98.1 - 99.1]	90.1	[87.3 - 92.3]	0.43
**'LcRL25.fna'**	**'LcRL31.fna'**	85.4	[81.6 - 88.4]	98.7	[98.1 - 99.1]	90.3	[87.6 - 92.5]	0.32
**'LcRL15.fna'**	**'LcRL23.fna'**	91.3	[88.2 - 93.6]	98.6	[97.9 - 99.0]	94.7	[92.7 - 96.1]	0.35

Query	Subject	d_0	C.I. d_0	d_4	C.I. d_4	d_6	C.I. d_6	Diff. G+C Percent
**'LcRL17.fna'**	**'LcRL23.fna'**	88.4	[85.0 - 91.2]	98.6	[97.9 - 99.0]	92.6	[90.2 - 94.5]	1.53
**'LcRL19.fna'**	**'LcRL31.fna'**	94.4	[92.0 - 96.2]	98.6	[97.9 - 99.1]	96.8	[95.3 - 97.8]	0.43
**'LcRL23.fna'**	**'LcRL30.fna'**	92.5	[89.7 - 94.6]	98.6	[97.9 - 99.1]	95.5	[93.7 - 96.8]	0.1
**'LcRL26.fna'**	**'LcRL31.fna'**	94.9	[92.6 - 96.6]	98.6	[97.9 - 99.0]	97.1	[95.7 - 98.0]	0.19
**'LcRL19.fna'**	**'LcRL32.fna'**	94.3	[91.8 - 96.0]	98.6	[97.9 - 99.0]	96.7	[95.2 - 97.7]	0.38
**'LcRL29.fna'**	**'LcRL31.fna'**	94.7	[92.3 - 96.4]	98.6	[97.9 - 99.0]	96.9	[95.5 - 97.9]	0.09
**'LcRL26.fna'**	**'LcRL32.fna'**	94.7	[92.3 - 96.3]	98.5	[97.8 - 99.0]	96.9	[95.5 - 97.9]	0.15
**'LcRL24.fna'**	**'LcRL31.fna'**	90.4	[87.2 - 92.9]	98.5	[97.8 - 99.0]	94.0	[91.9 - 95.6]	0.49
**'LcRL24.fna'**	**'LcRL32.fna'**	90.1	[86.8 - 92.6]	98.5	[97.8 - 99.0]	93.8	[91.6 - 95.4]	0.53
**'LcRL30.fna'**	**'LcRL33.fna'**	90.7	[87.5 - 93.1]	98.5	[97.8 - 99.0]	94.2	[92.1 - 95.8]	0.04
**'LcRL32.fna'**	**'LcRL33.fna'**	87.9	[84.4 - 90.7]	98.5	[97.7 - 99.0]	92.2	[89.8 - 94.1]	0.36
**'LcRL15.fna'**	**'LcRL33.fna'**	89.4	[86.1 - 92.0]	98.5	[97.8 - 99.0]	93.4	[91.1 - 95.1]	0.28
**'LcRL31.fna'**	**'LcRL33.fna'**	88.3	[84.8 - 91.0]	98.5	[97.8 - 99.0]	92.5	[90.1 - 94.4]	0.32
**'LcRL29.fna'**	**'LcRL32.fna'**	94.4	[91.9 - 96.1]	98.5	[97.7 - 99.0]	96.7	[95.2 - 97.8]	0.13
**'LcRL13.fna'**	**'LcRL27.fna'**	85.6	[82.0 - 88.7]	98.4	[97.6 - 98.9]	90.5	[87.8 - 92.7]	0.15
**'LcRL20.fna'**	**'LcRL30.fna'**	89.7	[86.4 - 92.2]	98.4	[97.6 - 98.9]	93.5	[91.3 - 95.2]	0.41
**'LcRL19.fna'**	**'LcRL20.fna'**	87.7	[84.2 - 90.5]	98.4	[97.6 - 98.9]	92.1	[89.6 - 94.0]	1.12
**'LcRL11.fna'**	**'LcRL27.fna'**	86.0	[82.4 - 89.0]	98.4	[97.6 - 98.9]	90.8	[88.1 - 92.9]	0.34
**'LcRL23.fna'**	**'LcRL24.fna'**	84.9	[81.2 - 88.0]	98.4	[97.6 - 98.9]	89.9	[87.2 - 92.2]	0.1
**'LcRL13.fna'**	**'LcRL21.fna'**	86.0	[82.4 - 89.0]	98.4	[97.6 - 98.9]	90.8	[88.2 - 92.9]	0.07
**'LcRL27.fna'**	**'LcRL28.fna'**	85.1	[81.4 - 88.2]	98.4	[97.6 - 98.9]	90.1	[87.4 - 92.3]	0.0
**'LcRL11.fna'**	**'LcRL21.fna'**	86.5	[82.9 - 89.5]	98.4	[97.6 - 98.9]	91.2	[88.6 - 93.2]	0.26
**'LcRL21.fna'**	**'LcRL28.fna'**	85.5	[81.8 - 88.6]	98.4	[97.7 - 98.9]	90.4	[87.7 - 92.6]	0.08
**'LcRL20.fna'**	**'LcRL26.fna'**	88.4	[84.9 - 91.1]	98.4	[97.6 - 98.9]	92.6	[90.2 - 94.4]	0.89
**'LcRL20.fna'**	**'LcRL29.fna'**	88.0	[84.5 - 90.8]	98.3	[97.6 - 98.9]	92.3	[89.9 - 94.2]	0.61
**'LcRL20.fna'**	**'LcRL33.fna'**	84.7	[80.9 - 87.8]	98.3	[97.6 - 98.9]	89.8	[87.0 - 92.0]	0.37
**'LcRL24.fna'**	**'LcRL25.fna'**	84.5	[80.8 - 87.7]	98.3	[97.6 - 98.9]	89.6	[86.8 - 91.9]	0.18

Query	Subject	d_0	C.I. d_0	d_4	C.I. d_4	d_6	C.I. d_6	Diff. G+C Percent
**'LcRL17.fna'**	**'LcRL19.fna'**	92.8	[90.0 - 94.9]	98.3	[97.6 - 98.9]	95.7	[93.9 - 97.0]	0.71
**'LcRL17.fna'**	**'LcRL20.fna'**	85.2	[81.4 - 88.2]	98.3	[97.6 - 98.9]	90.1	[87.4 - 92.3]	1.84
**'LcRL20.fna'**	**'LcRL24.fna'**	83.7	[79.9 - 86.9]	98.3	[97.5 - 98.8]	89.0	[86.1 - 91.3]	0.2
**'LcRL17.fna'**	**'LcRL26.fna'**	93.1	[90.4 - 95.1]	98.3	[97.5 - 98.8]	95.9	[94.1 - 97.1]	0.95
**'LcRL16.fna'**	**'LcRL28.fna'**	82.5	[78.6 - 85.8]	98.2	[97.4 - 98.8]	88.0	[85.0 - 90.4]	0.69
**'LcRL19.fna'**	**'LcRL30.fna'**	96.5	[94.6 - 97.7]	98.2	[97.3 - 98.7]	98.0	[96.9 - 98.7]	0.71
**'LcRL17.fna'**	**'LcRL29.fna'**	92.8	[90.0 - 94.9]	98.2	[97.4 - 98.8]	95.7	[93.9 - 96.9]	1.23
**'LcRL13.fna'**	**'LcRL16.fna'**	83.2	[79.3 - 86.4]	98.2	[97.4 - 98.7]	88.5	[85.6 - 90.9]	0.55
**'LcRL30.fna'**	**'LcRL31.fna'**	94.8	[92.4 - 96.4]	98.2	[97.4 - 98.8]	97.0	[95.5 - 97.9]	0.28
**'LcRL17.fna'**	**'LcRL24.fna'**	88.4	[85.0 - 91.2]	98.2	[97.4 - 98.8]	92.6	[90.2 - 94.4]	1.63
**'LcRL11.fna'**	**'LcRL16.fna'**	83.6	[79.8 - 86.8]	98.2	[97.4 - 98.8]	88.9	[86.0 - 91.2]	0.35
**'LcRL20.fna'**	**'LcRL27.fna'**	79.3	[75.4 - 82.8]	98.2	[97.4 - 98.7]	85.4	[82.2 - 88.1]	0.74
**'LcRL15.fna'**	**'LcRL19.fna'**	95.5	[93.3 - 97.0]	98.2	[97.3 - 98.7]	97.4	[96.1 - 98.3]	0.47
**'LcRL20.fna'**	**'LcRL21.fna'**	79.6	[75.6 - 83.0]	98.2	[97.4 - 98.8]	85.6	[82.4 - 88.2]	0.66
**'LcRL15.fna'**	**'LcRL26.fna'**	96.0	[94.0 - 97.4]	98.1	[97.3 - 98.7]	97.7	[96.5 - 98.5]	0.23
**'LcRL29.fna'**	**'LcRL30.fna'**	96.7	[94.9 - 97.9]	98.1	[97.2 - 98.7]	98.1	[97.1 - 98.8]	0.2
**'LcRL26.fna'**	**'LcRL30.fna'**	96.9	[95.2 - 98.0]	98.1	[97.3 - 98.7]	98.3	[97.3 - 98.9]	0.47
**'LcRL15.fna'**	**'LcRL24.fna'**	91.7	[88.8 - 94.0]	98.1	[97.2 - 98.7]	94.9	[93.0 - 96.3]	0.45
**'LcRL15.fna'**	**'LcRL29.fna'**	95.7	[93.6 - 97.2]	98.1	[97.2 - 98.7]	97.5	[96.3 - 98.4]	0.05
**'LcRL30.fna'**	**'LcRL32.fna'**	94.5	[92.0 - 96.2]	98.1	[97.3 - 98.7]	96.7	[95.2 - 97.8]	0.33
**'LcRL15.fna'**	**'LcRL20.fna'**	88.5	[85.1 - 91.2]	98.1	[97.3 - 98.7]	92.6	[90.2 - 94.4]	0.66
**'LcRL14.fna'**	**'LcRL33.fna'**	79.8	[75.9 - 83.3]	98.1	[97.2 - 98.7]	85.8	[82.6 - 88.4]	0.12
**'LcRL24.fna'**	**'LcRL30.fna'**	92.7	[89.9 - 94.8]	98.1	[97.2 - 98.7]	95.6	[93.8 - 96.9]	0.21
**'LcRL16.fna'**	**'LcRL20.fna'**	92.3	[89.4 - 94.5]	98.0	[97.1 - 98.6]	95.3	[93.4 - 96.6]	0.05
**'LcRL20.fna'**	**'LcRL32.fna'**	88.6	[85.2 - 91.3]	97.8	[96.9 - 98.5]	92.6	[90.2 - 94.5]	0.74
**'LcRL20.fna'**	**'LcRL31.fna'**	89.0	[85.6 - 91.6]	97.8	[96.9 - 98.5]	92.9	[90.6 - 94.7]	0.7
**'LcRL16.fna'**	**'LcRL19.fna'**	86.7	[83.1 - 89.6]	97.8	[96.8 - 98.4]	91.2	[88.6 - 93.2]	1.08

Query	Subject	d_0	C.I. d_0	d_4	C.I. d_4	d_6	C.I. d_6	Diff. G+C Percent
**'LcRL16.fna'**	**'LcRL29.fna'**	86.6	[83.0 - 89.6]	97.7	[96.7 - 98.4]	91.1	[88.5 - 93.2]	0.56
**'LcRL16.fna'**	**'LcRL26.fna'**	87.1	[83.5 - 89.9]	97.7	[96.8 - 98.4]	91.5	[88.9 - 93.5]	0.84
**'LcRL17.fna'**	**'LcRL31.fna'**	90.6	[87.5 - 93.1]	97.7	[96.7 - 98.4]	94.1	[92.0 - 95.7]	1.14
**'LcRL15.fna'**	**'LcRL31.fna'**	93.8	[91.2 - 95.7]	97.7	[96.8 - 98.4]	96.3	[94.6 - 97.4]	0.04
**'LcRL15.fna'**	**'LcRL32.fna'**	93.4	[90.8 - 95.4]	97.7	[96.7 - 98.4]	96.0	[94.3 - 97.2]	0.08
**'LcRL23.fna'**	**'LcRL27.fna'**	81.0	[77.0 - 84.3]	97.6	[96.6 - 98.3]	86.6	[83.5 - 89.2]	0.43
**'LcRL21.fna'**	**'LcRL23.fna'**	81.4	[77.5 - 84.7]	97.6	[96.6 - 98.3]	87.0	[83.9 - 89.5]	0.35
**'LcRL17.fna'**	**'LcRL32.fna'**	90.5	[87.3 - 92.9]	97.6	[96.7 - 98.3]	94.0	[91.8 - 95.6]	1.1
**'LcRL14.fna'**	**'LcRL20.fna'**	80.5	[76.5 - 83.9]	97.6	[96.6 - 98.3]	86.2	[83.1 - 88.8]	0.26
**'LcRL16.fna'**	**'LcRL23.fna'**	88.8	[85.4 - 91.5]	97.5	[96.5 - 98.3]	92.7	[90.4 - 94.6]	0.26
**'LcRL21.fna'**	**'LcRL25.fna'**	81.3	[77.4 - 84.6]	97.5	[96.5 - 98.3]	86.9	[83.8 - 89.4]	0.28
**'LcRL16.fna'**	**'LcRL25.fna'**	88.6	[85.1 - 91.3]	97.5	[96.5 - 98.3]	92.6	[90.2 - 94.4]	0.33
**'LcRL25.fna'**	**'LcRL27.fna'**	80.8	[76.9 - 84.2]	97.5	[96.5 - 98.3]	86.5	[83.4 - 89.1]	0.36
**'LcRL14.fna'**	**'LcRL28.fna'**	84.1	[80.3 - 87.2]	97.4	[96.4 - 98.2]	89.1	[86.2 - 91.4]	0.48
**'LcRL11.fna'**	**'LcRL14.fna'**	85.1	[81.4 - 88.2]	97.3	[96.3 - 98.1]	89.9	[87.1 - 92.1]	0.14
**'LcRL13.fna'**	**'LcRL14.fna'**	84.7	[80.9 - 87.8]	97.3	[96.3 - 98.1]	89.6	[86.8 - 91.8]	0.34
**'LcRL16.fna'**	**'LcRL31.fna'**	87.4	[83.9 - 90.3]	97.2	[96.1 - 98.0]	91.7	[89.1 - 93.7]	0.65
**'LcRL16.fna'**	**'LcRL33.fna'**	80.7	[76.8 - 84.1]	97.2	[96.1 - 98.0]	86.4	[83.3 - 89.0]	0.33
**'LcRL14.fna'**	**'LcRL19.fna'**	85.0	[81.3 - 88.1]	97.2	[96.0 - 98.0]	89.8	[87.0 - 92.0]	0.86
**'LcRL16.fna'**	**'LcRL32.fna'**	87.2	[83.7 - 90.1]	97.1	[96.0 - 98.0]	91.5	[89.0 - 93.5]	0.69
**'LcRL16.fna'**	**'LcRL30.fna'**	87.9	[84.4 - 90.7]	97.1	[96.0 - 97.9]	92.0	[89.5 - 93.9]	0.36
**'LcRL14.fna'**	**'LcRL26.fna'**	85.4	[81.7 - 88.5]	97.1	[95.9 - 97.9]	90.1	[87.4 - 92.3]	0.63
**'LcRL14.fna'**	**'LcRL29.fna'**	85.0	[81.2 - 88.1]	97.0	[95.9 - 97.8]	89.8	[87.0 - 92.0]	0.35
**'LcRL16.fna'**	**'LcRL24.fna'**	83.1	[79.3 - 86.4]	96.9	[95.8 - 97.8]	88.3	[85.3 - 90.7]	0.16
**'LcRL15.fna'**	**'LcRL16.fna'**	86.6	[83.0 - 89.6]	96.8	[95.6 - 97.7]	91.0	[88.4 - 93.1]	0.61
**'LcRL16.fna'**	**'LcRL17.fna'**	83.7	[79.9 - 86.9]	96.8	[95.6 - 97.7]	88.7	[85.8 - 91.1]	1.79
**'LcRL14.fna'**	**'LcRL24.fna'**	81.0	[77.1 - 84.4]	96.8	[95.6 - 97.7]	86.5	[83.5 - 89.1]	0.05

Query	Subject	d_0	C.I. d_0	d_4	C.I. d_4	d_6	C.I. d_6	Diff. G+C Percent
**'LcRL21.fna'**	**'LcRL33.fna'**	93.3	[90.7 - 95.3]	96.7	[95.5 - 97.6]	95.8	[94.1 - 97.1]	0.29
**'LcRL27.fna'**	**'LcRL31.fna'**	86.9	[83.3 - 89.8]	96.7	[95.5 - 97.6]	91.2	[88.6 - 93.2]	0.05
**'LcRL27.fna'**	**'LcRL32.fna'**	87.0	[83.4 - 89.9]	96.7	[95.4 - 97.6]	91.3	[88.7 - 93.3]	0.0
**'LcRL21.fna'**	**'LcRL32.fna'**	86.8	[83.2 - 89.7]	96.7	[95.4 - 97.6]	91.1	[88.5 - 93.2]	0.08
**'LcRL21.fna'**	**'LcRL31.fna'**	86.7	[83.1 - 89.6]	96.7	[95.4 - 97.6]	91.0	[88.4 - 93.1]	0.03
**'LcRL27.fna'**	**'LcRL33.fna'**	92.9	[90.2 - 95.0]	96.7	[95.5 - 97.6]	95.6	[93.8 - 96.9]	0.37
**'LcRL21.fna'**	**'LcRL26.fna'**	86.0	[82.3 - 89.0]	96.6	[95.3 - 97.5]	90.5	[87.8 - 92.6]	0.22
**'LcRL19.fna'**	**'LcRL21.fna'**	85.7	[82.0 - 88.7]	96.6	[95.4 - 97.6]	90.2	[87.5 - 92.4]	0.46
**'LcRL19.fna'**	**'LcRL27.fna'**	85.4	[81.7 - 88.4]	96.6	[95.4 - 97.5]	90.0	[87.2 - 92.2]	0.38
**'LcRL21.fna'**	**'LcRL30.fna'**	87.8	[84.3 - 90.6]	96.6	[95.3 - 97.5]	91.8	[89.3 - 93.8]	0.25
**'LcRL27.fna'**	**'LcRL29.fna'**	85.3	[81.6 - 88.3]	96.5	[95.2 - 97.4]	89.9	[87.1 - 92.1]	0.14
**'LcRL27.fna'**	**'LcRL30.fna'**	87.4	[83.9 - 90.3]	96.5	[95.3 - 97.5]	91.6	[89.0 - 93.6]	0.33
**'LcRL21.fna'**	**'LcRL29.fna'**	85.6	[81.9 - 88.6]	96.5	[95.2 - 97.5]	90.1	[87.4 - 92.3]	0.05
**'LcRL26.fna'**	**'LcRL27.fna'**	85.7	[82.0 - 88.7]	96.5	[95.3 - 97.5]	90.2	[87.5 - 92.4]	0.14
**'LcRL14.fna'**	**'LcRL23.fna'**	90.4	[87.2 - 92.8]	96.4	[95.1 - 97.3]	93.7	[91.5 - 95.4]	0.05
**'LcRL14.fna'**	**'LcRL32.fna'**	87.3	[83.7 - 90.1]	96.4	[95.1 - 97.3]	91.4	[88.8 - 93.4]	0.48
**'LcRL15.fna'**	**'LcRL21.fna'**	86.5	[82.9 - 89.5]	96.3	[95.0 - 97.3]	90.8	[88.2 - 92.9]	0.01
**'LcRL17.fna'**	**'LcRL27.fna'**	83.5	[79.7 - 86.7]	96.3	[95.0 - 97.3]	88.5	[85.5 - 90.9]	1.09
**'LcRL17.fna'**	**'LcRL21.fna'**	83.9	[80.1 - 87.1]	96.3	[95.0 - 97.3]	88.8	[85.9 - 91.1]	1.18
**'LcRL14.fna'**	**'LcRL31.fna'**	87.3	[83.7 - 90.1]	96.3	[95.0 - 97.3]	91.4	[88.8 - 93.4]	0.44
**'LcRL14.fna'**	**'LcRL25.fna'**	90.2	[87.0 - 92.7]	96.3	[95.0 - 97.3]	93.6	[91.4 - 95.3]	0.12
**'LcRL15.fna'**	**'LcRL27.fna'**	86.1	[82.4 - 89.1]	96.3	[94.9 - 97.3]	90.5	[87.8 - 92.6]	0.09
**'LcRL14.fna'**	**'LcRL27.fna'**	83.1	[79.3 - 86.3]	95.9	[94.4 - 96.9]	88.1	[85.1 - 90.5]	0.48
**'LcRL16.fna'**	**'LcRL27.fna'**	77.2	[73.2 - 80.7]	95.9	[94.5 - 97.0]	83.1	[79.9 - 86.0]	0.7
**'LcRL16.fna'**	**'LcRL21.fna'**	77.4	[73.4 - 80.9]	95.9	[94.5 - 97.0]	83.3	[80.1 - 86.2]	0.62
**'LcRL14.fna'**	**'LcRL21.fna'**	83.2	[79.4 - 86.4]	95.8	[94.4 - 96.9]	88.1	[85.2 - 90.6]	0.4
**'LcRL24.fna'**	**'LcRL27.fna'**	93.4	[90.7 - 95.3]	95.6	[94.1 - 96.7]	95.7	[93.9 - 97.0]	0.54

Query	Subject	d_0	C.I. d_0	d_4	C.I. d_4	d_6	C.I. d_6	Diff. G+C Percent
**'LcRL21.fna'**	**'LcRL24.fna'**	93.8	[91.2 - 95.7]	95.6	[94.1 - 96.7]	96.0	[94.3 - 97.2]	0.46
**'LcRL14.fna'**	**'LcRL30.fna'**	87.8	[84.3 - 90.6]	95.5	[94.0 - 96.6]	91.7	[89.2 - 93.7]	0.15
**'LcRL14.fna'**	**'LcRL15.fna'**	86.6	[83.0 - 89.6]	95.2	[93.6 - 96.4]	90.7	[88.1 - 92.9]	0.4
**'LcRL11.fna'**	**'LcRL13.fna'**	99.8	[99.5 - 99.9]	95.2	[93.6 - 96.4]	99.8	[99.6 - 99.9]	0.19
**'LcRL14.fna'**	**'LcRL17.fna'**	83.5	[79.6 - 86.7]	95.1	[93.5 - 96.3]	88.2	[85.3 - 90.7]	1.58
**'LcRL14.fna'**	**'LcRL16.fna'**	89.7	[86.4 - 92.3]	94.7	[93.1 - 96.0]	93.0	[90.6 - 94.8]	0.21
**'LcRL28.fna'**	*Lactobacillus crispatus* JCM 1185	66.1	[62.3 - 69.8]	77.3	[74.3 - 80.0]	70.1	[66.7 - 73.4]	0.71
**'LcRL23.fna'**	*Lactobacillus crispatus* JCM 1185	62.0	[58.3 - 65.6]	77.1	[74.1 - 79.8]	66.2	[62.9 - 69.5]	0.28
**'LcRL11.fna'**	*Lactobacillus crispatus* JCM 1185	67.5	[63.6 - 71.2]	76.9	[73.9 - 79.7]	71.4	[67.9 - 74.6]	0.38
**'LcRL25.fna'**	*Lactobacillus crispatus* JCM 1185	61.8	[58.1 - 65.4]	76.8	[73.8 - 79.6]	66.0	[62.7 - 69.3]	0.36
**'LcRL13.fna'**	*Lactobacillus crispatus* JCM 1185	66.6	[62.8 - 70.2]	76.8	[73.8 - 79.5]	70.5	[67.0 - 73.7]	0.57
**'LcRL16.fna'**	*Lactobacillus crispatus* JCM 1185	59.5	[55.8 - 63.0]	76.6	[73.7 - 79.4]	63.7	[60.4 - 66.9]	0.02
**'LcRL24.fna'**	*Lactobacillus crispatus* JCM 1185	63.7	[60.0 - 67.3]	76.6	[73.6 - 79.3]	67.8	[64.4 - 71.0]	0.18
**'LcRL32.fna'**	*Lactobacillus crispatus* JCM 1185	65.8	[62.0 - 69.4]	76.6	[73.6 - 79.3]	69.7	[66.3 - 73.0]	0.71
**'LcRL29.fna'**	*Lactobacillus crispatus* JCM 1185	67.5	[63.6 - 71.2]	76.5	[73.5 - 79.2]	71.3	[67.8 - 74.5]	0.58
**'LcRL26.fna'**	*Lactobacillus crispatus* JCM 1185	68.0	[64.1 - 71.6]	76.5	[73.5 - 79.3]	71.7	[68.2 - 74.9]	0.86
**'LcRL17.fna'**	*Lactobacillus crispatus* JCM 1185	63.0	[59.2 - 66.6]	76.5	[73.5 - 79.3]	67.1	[63.7 - 70.3]	1.81
**'LcRL19.fna'**	*Lactobacillus crispatus* JCM 1185	67.3	[63.4 - 70.9]	76.4	[73.4 - 79.2]	71.1	[67.6 - 74.3]	1.1
**'LcRL31.fna'**	*Lactobacillus crispatus* JCM 1185	65.6	[61.7 - 69.2]	76.4	[73.4 - 79.2]	69.5	[66.0 - 72.7]	0.67
**'LcRL20.fna'**	*Lactobacillus crispatus* JCM 1185	62.9	[59.2 - 66.5]	76.4	[73.4 - 79.2]	67.0	[63.6 - 70.2]	0.02
**'LcRL30.fna'**	*Lactobacillus crispatus* JCM 1185	68.5	[64.6 - 72.1]	76.3	[73.3 - 79.1]	72.1	[68.7 - 75.3]	0.39
**'LcRL14.fna'**	*Lactobacillus crispatus* JCM 1185	59.6	[56.0 - 63.2]	76.2	[73.2 - 79.0]	63.8	[60.5 - 67.0]	0.23
**'LcRL33.fna'**	*Lactobacillus crispatus* JCM 1185	64.3	[60.5 - 67.9]	76.2	[73.2 - 79.0]	68.3	[64.8 - 71.5]	0.35
**'LcRL15.fna'**	*Lactobacillus crispatus* JCM 1185	67.6	[63.8 - 71.3]	76.1	[73.1 - 78.8]	71.3	[67.9 - 74.6]	0.63
**'LcRL27.fna'**	*Lactobacillus crispatus* JCM 1185	59.7	[56.0 - 63.2]	76.0	[73.0 - 78.8]	63.8	[60.5 - 67.1]	0.72
**'LcRL21.fna'**	*Lactobacillus crispatus* JCM 1185	59.9	[56.3 - 63.5]	76.0	[73.0 - 78.8]	64.1	[60.7 - 67.3]	0.64
**'LcRL13.fna'**	*Lactobacillus gallinarum* JCM 2011	31.8	[28.4 - 35.4]	25.0	[22.7 - 27.5]	29.1	[26.2 - 32.2]	0.68

Query	Subject	d_0	C.I. d_0	d_4	C.I. d_4	d_6	C.I. d_6	Diff. G+C Percent
**'LcRL11.fna'**	*Lactobacillus gallinarum* JCM 2011	31.4	[28.1 - 35.0]	24.9	[22.6 - 27.4]	28.8	[25.9 - 31.9]	0.49
**'LcRL27.fna'**	*Lactobacillus gallinarum* JCM 2011	29.3	[25.9 - 32.9]	24.9	[22.6 - 27.4]	27.2	[24.3 - 30.3]	0.83
**'LcRL20.fna'**	*Lactobacillus gallinarum* JCM 2011	30.0	[26.6 - 33.6]	24.9	[22.6 - 27.4]	27.7	[24.8 - 30.8]	0.09
**'LcRL21.fna'**	*Lactobacillus gallinarum* JCM 2011	29.4	[26.0 - 33.0]	24.9	[22.5 - 27.3]	27.3	[24.4 - 30.4]	0.75
**'LcRL14.fna'**	*Lactobacillus gallinarum* JCM 2011	29.1	[25.7 - 32.7]	24.9	[22.5 - 27.3]	27.1	[24.2 - 30.2]	0.35
**'LcRL29.fna'**	*Lactobacillus gallinarum* JCM 2011	31.7	[28.3 - 35.3]	24.9	[22.6 - 27.4]	29.1	[26.1 - 32.2]	0.7
**'LcRL31.fna'**	*Lactobacillus gallinarum* JCM 2011	30.9	[27.5 - 34.5]	24.9	[22.6 - 27.4]	28.5	[25.5 - 31.6]	0.79
**'LcRL28.fna'**	*Lactobacillus gallinarum* JCM 2011	31.6	[28.2 - 35.2]	24.9	[22.6 - 27.4]	29.0	[26.0 - 32.1]	0.83
**'LcRL33.fna'**	*Lactobacillus gallinarum* JCM 2011	30.5	[27.1 - 34.1]	24.9	[22.6 - 27.4]	28.1	[25.2 - 31.2]	0.46
**'LcRL32.fna'**	*Lactobacillus gallinarum* JCM 2011	31.0	[27.7 - 34.6]	24.8	[22.5 - 27.3]	28.5	[25.6 - 31.6]	0.83
**'LcRL19.fna'**	*Lactobacillus gallinarum* JCM 2011	31.3	[27.9 - 34.9]	24.8	[22.5 - 27.3]	28.7	[25.8 - 31.8]	1.21
**'LcRL30.fna'**	*Lactobacillus gallinarum* JCM 2011	31.2	[27.8 - 34.8]	24.8	[22.4 - 27.2]	28.6	[25.7 - 31.7]	0.5
**'LcRL16.fna'**	*Lactobacillus gallinarum* JCM 2011	29.1	[25.8 - 32.7]	24.8	[22.5 - 27.3]	27.1	[24.2 - 30.2]	0.14
**'LcRL26.fna'**	*Lactobacillus gallinarum* JCM 2011	31.3	[28.0 - 34.9]	24.7	[22.4 - 27.2]	28.7	[25.8 - 31.8]	0.97
**'LcRL17.fna'**	*Lactobacillus gallinarum* JCM 2011	29.9	[26.5 - 33.5]	24.7	[22.3 - 27.1]	27.6	[24.7 - 30.7]	1.93
**'LcRL24.fna'**	*Lactobacillus gallinarum* JCM 2011	30.3	[26.9 - 33.9]	24.6	[22.3 - 27.1]	27.9	[25.0 - 31.0]	0.29
**'LcRL15.fna'**	*Lactobacillus gallinarum* JCM 2011	31.0	[27.7 - 34.6]	24.6	[22.3 - 27.1]	28.5	[25.6 - 31.6]	0.75
**'LcRL23.fna'**	*Lactobacillus gallinarum* JCM 2011	31.1	[27.7 - 34.7]	24.6	[22.3 - 27.1]	28.5	[25.6 - 31.6]	0.4
**'LcRL25.fna'**	*Lactobacillus gallinarum* JCM 2011	31.1	[27.8 - 34.7]	24.6	[22.3 - 27.1]	28.6	[25.6 - 31.7]	0.47
**'LcRL27.fna'**	*Lactobacillus suntoryeus* LMG 22464	27.5	[24.2 - 31.2]	24.4	[22.1 - 26.9]	25.8	[22.9 - 28.9]	0.78
**'LcRL11.fna'**	*Lactobacillus suntoryeus* LMG 22464	30.3	[26.9 - 33.9]	24.4	[22.0 - 26.8]	27.9	[25.0 - 31.0]	0.44
**'LcRL11.fna'**	*Lactobacillus amylovorus* DSM 20531	32.4	[29.0 - 35.9]	24.4	[22.0 - 26.8]	29.4	[26.5 - 32.5]	0.81
**'LcRL28.fna'**	*Lactobacillus suntoryeus* LMG 22464	30.1	[26.7 - 33.7]	24.4	[22.1 - 26.9]	27.7	[24.8 - 30.8]	0.78
**'LcRL16.fna'**	*Lactobacillus suntoryeus* LMG 22464	27.8	[24.5 - 31.5]	24.4	[22.0 - 26.8]	26.0	[23.1 - 29.1]	0.09
**'LcRL26.fna'**	*Lactobacillus suntoryeus* LMG 22464	29.6	[26.2 - 33.2]	24.4	[22.1 - 26.9]	27.3	[24.4 - 30.4]	0.92
**'LcRL33.fna'**	*Lactobacillus suntoryeus* LMG 22464	29.0	[25.7 - 32.7]	24.4	[22.1 - 26.9]	26.9	[24.0 - 30.0]	0.41
**'LcRL14.fna'**	*Lactobacillus suntoryeus* LMG 22464	27.4	[24.1 - 31.1]	24.4	[22.1 - 26.9]	25.7	[22.8 - 28.8]	0.3

Query	Subject	d_0	C.I. d_0	d_4	C.I. d_4	d_6	C.I. d_6	Diff. G+C Percent
**'LcRL32.fna'**	*Lactobacillus suntoryeus* LMG 22464	29.2	[25.9 - 32.8]	24.4	[22.0 - 26.8]	27.0	[24.1 - 30.2]	0.78
**'LcRL13.fna'**	*Lactobacillus suntoryeus* LMG 22464	30.2	[26.9 - 33.8]	24.4	[22.1 - 26.9]	27.8	[24.9 - 30.9]	0.63
**'LcRL31.fna'**	*Lactobacillus suntoryeus* LMG 22464	29.1	[25.7 - 32.7]	24.4	[22.1 - 26.9]	26.9	[24.0 - 30.0]	0.73
**'LcRL20.fna'**	*Lactobacillus amylovorus* DSM 20531	31.3	[27.9 - 34.9]	24.4	[22.1 - 26.9]	28.6	[25.7 - 31.7]	1.21
**'LcRL29.fna'**	*Lactobacillus suntoryeus* LMG 22464	30.2	[26.8 - 33.8]	24.4	[22.1 - 26.9]	27.8	[24.9 - 30.9]	0.65
**'LcRL21.fna'**	*Lactobacillus suntoryeus* LMG 22464	27.6	[24.2 - 31.2]	24.4	[22.0 - 26.8]	25.8	[22.9 - 28.9]	0.7
**'LcRL30.fna'**	*Lactobacillus suntoryeus* LMG 22464	29.7	[26.4 - 33.3]	24.3	[22.0 - 26.7]	27.4	[24.5 - 30.5]	0.45
**'LcRL33.fna'**	*Lactobacillus amylovorus* DSM 20531	31.5	[28.1 - 35.0]	24.3	[22.0 - 26.8]	28.7	[25.8 - 31.8]	0.84
**'LcRL17.fna'**	*Lactobacillus suntoryeus* LMG 22464	28.7	[25.3 - 32.3]	24.3	[22.0 - 26.7]	26.6	[23.7 - 29.7]	1.88
**'LcRL16.fna'**	*Lactobacillus amylovorus* DSM 20531	29.7	[26.3 - 33.3]	24.3	[22.0 - 26.8]	27.4	[24.5 - 30.5]	1.16
**'LcRL19.fna'**	*Lactobacillus amylovorus* DSM 20531	31.9	[28.5 - 35.5]	24.3	[22.0 - 26.8]	29.0	[26.1 - 32.1]	0.09
**'LcRL28.fna'**	*Lactobacillus amylovorus* DSM 20531	32.8	[29.4 - 36.4]	24.3	[22.0 - 26.7]	29.7	[26.8 - 32.8]	0.47
**'LcRL24.fna'**	*Lactobacillus suntoryeus* LMG 22464	28.8	[25.4 - 32.4]	24.3	[22.0 - 26.8]	26.7	[23.8 - 29.8]	0.24
**'LcRL25.fna'**	*Lactobacillus suntoryeus* LMG 22464	29.2	[25.8 - 32.8]	24.3	[21.9 - 26.7]	27.0	[24.1 - 30.1]	0.42
**'LcRL15.fna'**	*Lactobacillus suntoryeus* LMG 22464	29.6	[26.2 - 33.2]	24.3	[22.0 - 26.7]	27.3	[24.4 - 30.4]	0.7
**'LcRL19.fna'**	*Lactobacillus suntoryeus* LMG 22464	29.6	[26.2 - 33.2]	24.3	[22.0 - 26.8]	27.3	[24.4 - 30.4]	1.16
**'LcRL13.fna'**	*Lactobacillus amylovorus* DSM 20531	32.9	[29.5 - 36.4]	24.3	[22.0 - 26.8]	29.8	[26.8 - 32.9]	0.62
**'LcRL29.fna'**	*Lactobacillus amylovorus* DSM 20531	32.4	[29.0 - 36.0]	24.3	[22.0 - 26.8]	29.4	[26.5 - 32.5]	0.6
**'LcRL20.fna'**	*Lactobacillus suntoryeus* LMG 22464	28.4	[25.0 - 32.0]	24.3	[22.0 - 26.8]	26.4	[23.5 - 29.5]	0.04
**'LcRL24.fna'**	*Lactobacillus amylovorus* DSM 20531	31.3	[28.0 - 34.9]	24.3	[22.0 - 26.7]	28.6	[25.7 - 31.7]	1.01
**'LcRL14.fna'**	*Lactobacillus amylovorus* DSM 20531	29.6	[26.2 - 33.2]	24.3	[22.0 - 26.8]	27.3	[24.4 - 30.4]	0.95
**'LcRL23.fna'**	*Lactobacillus suntoryeus* LMG 22464	29.2	[25.8 - 32.8]	24.3	[21.9 - 26.7]	27.0	[24.1 - 30.1]	0.35
**'LcRL26.fna'**	*Lactobacillus amylovorus* DSM 20531	32.2	[28.8 - 35.8]	24.3	[22.0 - 26.8]	29.3	[26.3 - 32.4]	0.33
**'LcRL30.fna'**	*Lactobacillus amylovorus* DSM 20531	31.8	[28.4 - 35.4]	24.2	[21.9 - 26.6]	28.9	[26.0 - 32.0]	0.8
**'LcRL11.fna'**	*Lactobacillus kitasatonis* JCM 1039	32.1	[28.8 - 35.7]	24.2	[21.9 - 26.7]	29.2	[26.3 - 32.3]	0.54
**'LcRL32.fna'**	*Lactobacillus amylovorus* DSM 20531	31.7	[28.3 - 35.2]	24.2	[21.9 - 26.7]	28.8	[25.9 - 31.9]	0.47
**'LcRL28.fna'**	*Lactobacillus sobrius* DSM 16698	31.6	[28.2 - 35.1]	24.2	[21.9 - 26.7]	28.8	[25.8 - 31.9]	0.53

Query	Subject	d_0	C.I. d_0	d_4	C.I. d_4	d_6	C.I. d_6	Diff. G+C Percent
**'LcRL13.fna'**	*Lactobacillus sobrius* DSM 16698	31.7	[28.3 - 35.3]	24.2	[21.9 - 26.7]	28.8	[25.9 - 32.0]	0.67
**'LcRL21.fna'**	*Lactobacillus amylovorus* DSM 20531	30.0	[26.6 - 33.6]	24.2	[21.9 - 26.7]	27.6	[24.7 - 30.7]	0.55
**'LcRL23.fna'**	*Lactobacillus amylovorus* DSM 20531	31.6	[28.2 - 35.2]	24.2	[21.9 - 26.6]	28.8	[25.8 - 31.9]	0.9
**'LcRL11.fna'**	*Lactobacillus sobrius* DSM 16698	31.6	[28.2 - 35.1]	24.2	[21.8 - 26.6]	28.7	[25.8 - 31.8]	0.86
**'LcRL27.fna'**	*Lactobacillus amylovorus* DSM 20531	29.7	[26.3 - 33.3]	24.2	[21.9 - 26.7]	27.4	[24.4 - 30.5]	0.47
**'LcRL25.fna'**	*Lactobacillus amylovorus* DSM 20531	31.6	[28.3 - 35.2]	24.2	[21.9 - 26.6]	28.8	[25.9 - 31.9]	0.83
**'LcRL31.fna'**	*Lactobacillus amylovorus* DSM 20531	31.5	[28.1 - 35.1]	24.2	[21.9 - 26.7]	28.7	[25.8 - 31.8]	0.51
**'LcRL16.fna'**	*Lactobacillus kitasatonis* JCM 1039	29.2	[25.8 - 32.8]	24.1	[21.8 - 26.6]	27.0	[24.1 - 30.1]	0.89
**'LcRL25.fna'**	*Lactobacillus kitasatonis* JCM 1039	31.0	[27.7 - 34.6]	24.1	[21.8 - 26.5]	28.3	[25.4 - 31.5]	0.56
**'LcRL15.fna'**	*Lactobacillus amylovorus* DSM 20531	31.8	[28.5 - 35.4]	24.1	[21.8 - 26.6]	28.9	[26.0 - 32.0]	0.55
**'LcRL14.fna'**	*Lactobacillus kitasatonis* JCM 1039	29.4	[26.0 - 33.0]	24.1	[21.8 - 26.6]	27.1	[24.2 - 30.2]	0.68
**'LcRL32.fna'**	*Lactobacillus kitasatonis* JCM 1039	31.2	[27.8 - 34.8]	24.1	[21.8 - 26.6]	28.5	[25.6 - 31.6]	0.2
**'LcRL23.fna'**	*Lactobacillus kitasatonis* JCM 1039	31.1	[27.8 - 34.7]	24.1	[21.8 - 26.6]	28.4	[25.5 - 31.5]	0.63
**'LcRL28.fna'**	*Lactobacillus kitasatonis* JCM 1039	32.1	[28.7 - 35.6]	24.1	[21.8 - 26.6]	29.1	[26.2 - 32.2]	0.2
**'LcRL19.fna'**	*Lactobacillus helveticus* DSM 20075	26.9	[23.5 - 30.5]	24.1	[21.7 - 26.5]	25.2	[22.3 - 28.3]	0.93
**'LcRL26.fna'**	*Lactobacillus helveticus* DSM 20075	27.0	[23.6 - 30.6]	24.1	[21.8 - 26.6]	25.3	[22.4 - 28.4]	0.69
**'LcRL19.fna'**	*Lactobacillus sobrius* DSM 16698	31.0	[27.6 - 34.6]	24.1	[21.8 - 26.5]	28.3	[25.4 - 31.4]	0.14
**'LcRL21.fna'**	*Lactobacillus helveticus* DSM 20075	25.2	[21.9 - 28.9]	24.1	[21.8 - 26.6]	23.9	[21.1 - 27.0]	0.47
**'LcRL28.fna'**	*Lactobacillus helveticus* DSM 20075	27.3	[23.9 - 30.9]	24.1	[21.8 - 26.6]	25.5	[22.6 - 28.6]	0.55
**'LcRL29.fna'**	*Lactobacillus kitasatonis* JCM 1039	31.6	[28.2 - 35.2]	24.1	[21.8 - 26.6]	28.8	[25.9 - 31.9]	0.33
**'LcRL16.fna'**	*Lactobacillus sobrius* DSM 16698	29.2	[25.8 - 32.8]	24.1	[21.8 - 26.5]	26.9	[24.0 - 30.0]	1.22
**'LcRL33.fna'**	*Lactobacillus kitasatonis* JCM 1039	30.7	[27.3 - 34.3]	24.1	[21.8 - 26.6]	28.1	[25.2 - 31.2]	0.57
**'LcRL20.fna'**	*Lactobacillus helveticus* DSM 20075	26.1	[22.8 - 29.7]	24.1	[21.8 - 26.5]	24.6	[21.7 - 27.7]	0.19
**'LcRL31.fna'**	*Lactobacillus kitasatonis* JCM 1039	31.0	[27.6 - 34.6]	24.1	[21.8 - 26.6]	28.3	[25.4 - 31.4]	0.25
**'LcRL26.fna'**	*Lactobacillus kitasatonis* JCM 1039	31.5	[28.1 - 35.1]	24.1	[21.8 - 26.6]	28.7	[25.8 - 31.8]	0.06
**'LcRL20.fna'**	*Lactobacillus sobrius* DSM 16698	30.0	[26.7 - 33.6]	24.1	[21.8 - 26.5]	27.6	[24.7 - 30.7]	1.27
**'LcRL14.fna'**	*Lactobacillus sobrius* DSM 16698	29.3	[25.9 - 32.9]	24.1	[21.7 - 26.5]	27.0	[24.1 - 30.1]	1.01

Query	Subject	d_0	C.I. d_0	d_4	C.I. d_4	d_6	C.I. d_6	Diff. G+C Percent
**'LcRL17.fna'**	*Lactobacillus amylovorus* DSM 20531	30.8	[27.4 - 34.4]	24.1	[21.8 - 26.6]	28.2	[25.3 - 31.3]	0.63
**'LcRL13.fna'**	*Lactobacillus kitasatonis* JCM 1039	32.0	[28.6 - 35.5]	24.1	[21.8 - 26.6]	29.0	[26.1 - 32.1]	0.35
**'LcRL27.fna'**	*Lactobacillus helveticus* DSM 20075	25.1	[21.8 - 28.8]	24.1	[21.8 - 26.6]	23.8	[21.0 - 26.9]	0.55
**'LcRL24.fna'**	*Lactobacillus kitasatonis* JCM 1039	30.5	[27.1 - 34.1]	24.1	[21.8 - 26.6]	27.9	[25.0 - 31.1]	0.74
**'LcRL13.fna'**	*Lactobacillus helveticus* DSM 20075	27.4	[24.0 - 31.0]	24.1	[21.8 - 26.6]	25.6	[22.7 - 28.7]	0.4
**'LcRL11.fna'**	*Lactobacillus helveticus* DSM 20075	27.6	[24.2 - 31.2]	24.1	[21.8 - 26.6]	25.7	[22.8 - 28.8]	0.21
**'LcRL33.fna'**	*Lactobacillus sobrius* DSM 16698	30.5	[27.1 - 34.1]	24.1	[21.7 - 26.5]	27.9	[25.0 - 31.0]	0.89
**'LcRL14.fna'**	*Lactobacillus helveticus* DSM 20075	25.3	[22.0 - 29.0]	24.1	[21.8 - 26.6]	24.0	[21.1 - 27.1]	0.07
**'LcRL19.fna'**	*Lactobacillus kitasatonis* JCM 1039	31.2	[27.8 - 34.8]	24.1	[21.8 - 26.6]	28.5	[25.6 - 31.6]	0.18
**'LcRL21.fna'**	*Lactobacillus kitasatonis* JCM 1039	29.4	[26.1 - 33.0]	24.1	[21.8 - 26.6]	27.1	[24.2 - 30.2]	0.28
**'LcRL27.fna'**	*Lactobacillus sobrius* DSM 16698	29.1	[25.7 - 32.7]	24.1	[21.8 - 26.6]	26.9	[24.0 - 30.0]	0.52
**'LcRL27.fna'**	*Lactobacillus kitasatonis* JCM 1039	29.3	[25.9 - 32.9]	24.1	[21.8 - 26.6]	27.0	[24.1 - 30.1]	0.2
**'LcRL17.fna'**	*Lactobacillus helveticus* DSM 20075	26.1	[22.7 - 29.7]	24.0	[21.7 - 26.5]	24.6	[21.7 - 27.7]	1.65
**'LcRL17.fna'**	*Lactobacillus sobrius* DSM 16698	30.1	[26.8 - 33.8]	24.0	[21.7 - 26.4]	27.7	[24.7 - 30.8]	0.57
**'LcRL31.fna'**	*Lactobacillus helveticus* DSM 20075	26.5	[23.2 - 30.2]	24.0	[21.7 - 26.5]	24.9	[22.0 - 28.0]	0.5
**'LcRL20.fna'**	*Lactobacillus kitasatonis* JCM 1039	30.8	[27.4 - 34.4]	24.0	[21.7 - 26.5]	28.1	[25.2 - 31.3]	0.94
**'LcRL30.fna'**	*Lactobacillus sobrius* DSM 16698	31.1	[27.7 - 34.7]	24.0	[21.7 - 26.5]	28.4	[25.5 - 31.5]	0.85
**'LcRL32.fna'**	*Lactobacillus helveticus* DSM 20075	26.7	[23.4 - 30.4]	24.0	[21.7 - 26.5]	25.1	[22.2 - 28.2]	0.55
**'LcRL17.fna'**	*Lactobacillus kitasatonis* JCM 1039	30.6	[27.2 - 34.2]	24.0	[21.7 - 26.5]	28.0	[25.1 - 31.1]	0.9
**'LcRL25.fna'**	*Lactobacillus sobrius* DSM 16698	30.9	[27.5 - 34.5]	24.0	[21.7 - 26.5]	28.2	[25.3 - 31.3]	0.89
**'LcRL21.fna'**	*Lactobacillus sobrius* DSM 16698	29.2	[25.8 - 32.8]	24.0	[21.7 - 26.5]	27.0	[24.1 - 30.1]	0.6
**'LcRL24.fna'**	*Lactobacillus sobrius* DSM 16698	30.4	[27.0 - 34.0]	24.0	[21.7 - 26.5]	27.9	[25.0 - 31.0]	1.06
**'LcRL30.fna'**	*Lactobacillus helveticus* DSM 20075	26.8	[23.4 - 30.4]	24.0	[21.7 - 26.5]	25.1	[22.2 - 28.2]	0.22
**'LcRL29.fna'**	*Lactobacillus helveticus* DSM 20075	27.3	[23.9 - 30.9]	24.0	[21.7 - 26.5]	25.5	[22.6 - 28.6]	0.42
**'LcRL29.fna'**	*Lactobacillus sobrius* DSM 16698	31.4	[28.0 - 35.0]	24.0	[21.7 - 26.5]	28.6	[25.7 - 31.7]	0.66
**'LcRL26.fna'**	*Lactobacillus sobrius* DSM 16698	31.3	[27.9 - 34.9]	24.0	[21.7 - 26.5]	28.5	[25.6 - 31.6]	0.38
**'LcRL16.fna'**	*Lactobacillus helveticus* DSM 20075	25.7	[22.3 - 29.3]	24.0	[21.7 - 26.5]	24.2	[21.4 - 27.3]	0.15

Query	Subject	d_0	C.I. d_0	d_4	C.I. d_4	d_6	C.I. d_6	Diff. G+C Percent
**'LcRL23.fna'**	*Lactobacillus sobrius* DSM 16698	31.2	[27.8 - 34.8]	24.0	[21.7 - 26.5]	28.4	[25.5 - 31.5]	0.96
**'LcRL32.fna'**	*Lactobacillus sobrius* DSM 16698	31.0	[27.6 - 34.6]	24.0	[21.7 - 26.4]	28.3	[25.4 - 31.4]	0.53
**'LcRL31.fna'**	*Lactobacillus sobrius* DSM 16698	30.9	[27.5 - 34.5]	24.0	[21.7 - 26.5]	28.2	[25.3 - 31.4]	0.57
**'LcRL15.fna'**	*Lactobacillus kitasatonis* JCM 1039	31.5	[28.1 - 35.1]	24.0	[21.7 - 26.5]	28.7	[25.8 - 31.8]	0.28
**'LcRL24.fna'**	*Lactobacillus helveticus* DSM 20075	26.4	[23.0 - 30.0]	24.0	[21.7 - 26.5]	24.8	[21.9 - 27.9]	0.01
**'LcRL23.fna'**	*Lactobacillus helveticus* DSM 20075	26.8	[23.5 - 30.5]	24.0	[21.7 - 26.4]	25.1	[22.2 - 28.2]	0.12
**'LcRL25.fna'**	*Lactobacillus helveticus* DSM 20075	26.9	[23.6 - 30.5]	24.0	[21.7 - 26.4]	25.2	[22.3 - 28.3]	0.19
**'LcRL33.fna'**	*Lactobacillus helveticus* DSM 20075	26.5	[23.2 - 30.1]	24.0	[21.7 - 26.5]	24.9	[22.0 - 28.0]	0.18
**'LcRL15.fna'**	*Lactobacillus helveticus* DSM 20075	26.7	[23.4 - 30.4]	24.0	[21.7 - 26.5]	25.1	[22.2 - 28.2]	0.46
**'LcRL30.fna'**	*Lactobacillus kitasatonis* JCM 1039	31.7	[28.3 - 35.3]	24.0	[21.7 - 26.5]	28.8	[25.9 - 31.9]	0.53
**'LcRL15.fna'**	*Lactobacillus sobrius* DSM 16698	31.1	[27.8 - 34.7]	23.9	[21.6 - 26.4]	28.4	[25.5 - 31.5]	0.61
**'LcRL11.fna'**	*Lactobacillus ultunensis* DSM 16047	28.3	[25.0 - 32.0]	23.5	[21.2 - 25.9]	26.2	[23.3 - 29.3]	1.03
**'LcRL26.fna'**	*Lactobacillus kefiranofaciens* ATCC 43761	28.0	[24.6 - 31.6]	23.5	[21.2 - 26.0]	25.9	[23.1 - 29.0]	0.23
**'LcRL16.fna'**	*Lactobacillus kefiranofaciens* ATCC 43761	26.3	[23.0 - 30.0]	23.5	[21.2 - 25.9]	24.7	[21.8 - 27.8]	0.6
**'LcRL33.fna'**	*Lactobacillus kefiranofaciens* ATCC 43761	27.5	[24.1 - 31.1]	23.5	[21.2 - 26.0]	25.5	[22.7 - 28.6]	0.28
**'LcRL27.fna'**	*Lactobacillus kefiranofaciens* ATCC 43761	26.2	[22.9 - 29.9]	23.5	[21.2 - 26.0]	24.6	[21.7 - 27.7]	0.09
**'LcRL11.fna'**	*Lactobacillus kefiranofaciens* ATCC 43761	28.2	[24.8 - 31.8]	23.5	[21.2 - 26.0]	26.1	[23.2 - 29.2]	0.25
**'LcRL29.fna'**	*Lactobacillus ultunensis* DSM 16047	28.6	[25.3 - 32.3]	23.5	[21.2 - 26.0]	26.4	[23.5 - 29.5]	1.24
**'LcRL29.fna'**	*Lactobacillus kefiranofaciens* ATCC 43761	28.5	[25.1 - 32.1]	23.5	[21.2 - 25.9]	26.3	[23.4 - 29.4]	0.04
**'LcRL28.fna'**	*Lactobacillus kefiranofaciens* ATCC 43761	28.2	[24.8 - 31.8]	23.5	[21.2 - 26.0]	26.1	[23.2 - 29.2]	0.09
**'LcRL21.fna'**	*Lactobacillus kefiranofaciens* ATCC 43761	26.3	[22.9 - 29.9]	23.5	[21.2 - 25.9]	24.6	[21.7 - 27.7]	0.01
**'LcRL13.fna'**	*Lactobacillus kefiranofaciens* ATCC 43761	28.2	[24.9 - 31.8]	23.5	[21.2 - 26.0]	26.1	[23.2 - 29.2]	0.06
**'LcRL19.fna'**	*Lactobacillus kefiranofaciens* ATCC 43761	27.8	[24.5 - 31.5]	23.5	[21.2 - 25.9]	25.8	[22.9 - 28.9]	0.47
**'LcRL13.fna'**	*Lactobacillus ultunensis* DSM 16047	28.4	[25.0 - 32.0]	23.5	[21.2 - 25.9]	26.2	[23.3 - 29.3]	1.23

Query	Subject	d_0	C.I. d_0	d_4	C.I. d_4	d_6	C.I. d_6	Diff. G+C Percent
**'LcRL31.fna'**	*Lactobacillus kefiranofaciens* ATCC 43761	27.6	[24.3 - 31.3]	23.4	[21.1 - 25.9]	25.6	[22.8 - 28.7]	0.05
**'LcRL28.fna'**	*Lactobacillus ultunensis* DSM 16047	28.3	[25.0 - 32.0]	23.4	[21.1 - 25.9]	26.2	[23.3 - 29.3]	1.37
**'LcRL32.fna'**	*Lactobacillus kefiranofaciens* ATCC 43761	27.7	[24.4 - 31.4]	23.4	[21.1 - 25.9]	25.7	[22.8 - 28.8]	0.09
**'LcRL26.fna'**	*Lactobacillus kefiranofaciens* subsp. *kefirgranum* JCM 8572	28.2	[24.9 - 31.9]	23.4	[21.1 - 25.9]	26.1	[23.2 - 29.2]	0.03
**'LcRL24.fna'**	*Lactobacillus kefiranofaciens* ATCC 43761	27.2	[23.9 - 30.8]	23.4	[21.1 - 25.9]	25.3	[22.4 - 28.4]	0.45
**'LcRL20.fna'**	*Lactobacillus kefiranofaciens* ATCC 43761	27.1	[23.7 - 30.7]	23.4	[21.1 - 25.9]	25.2	[22.3 - 28.3]	0.65
**'LcRL33.fna'**	*Lactobacillus kefiranofaciens* subsp. *kefirgranum* JCM 8572	27.7	[24.3 - 31.3]	23.4	[21.1 - 25.8]	25.7	[22.8 - 28.8]	0.48
**'LcRL28.fna'**	*Lactobacillus kefiranofaciens* subsp. *kefirgranum* JCM 8572	28.5	[25.1 - 32.1]	23.4	[21.1 - 25.8]	26.3	[23.4 - 29.4]	0.12
**'LcRL29.fna'**	*Lactobacillus kefiranofaciens* subsp. *kefirgranum* JCM 8572	28.7	[25.3 - 32.3]	23.4	[21.1 - 25.8]	26.4	[23.5 - 29.5]	0.25
**'LcRL30.fna'**	*Lactobacillus kefiranofaciens* ATCC 43761	27.8	[24.4 - 31.4]	23.4	[21.1 - 25.8]	25.7	[22.9 - 28.8]	0.24
**'LcRL11.fna'**	*Lactobacillus kefiranofaciens* subsp. *kefirgranum* JCM 8572	28.5	[25.1 - 32.1]	23.4	[21.1 - 25.9]	26.3	[23.4 - 29.4]	0.46
**'LcRL13.fna'**	*Lactobacillus kefiranofaciens* subsp. *kefirgranum* JCM 8572	28.7	[25.3 - 32.3]	23.4	[21.1 - 25.9]	26.4	[23.5 - 29.5]	0.26
**'LcRL16.fna'**	*Lactobacillus kefiranofaciens* subsp. *kefirgranum* JCM 8572	26.6	[23.3 - 30.3]	23.4	[21.1 - 25.8]	24.9	[22.0 - 28.0]	0.81
**'LcRL33.fna'**	*Lactobacillus ultunensis* DSM 16047	27.9	[24.5 - 31.5]	23.4	[21.1 - 25.8]	25.8	[22.9 - 28.9]	1.01
**'LcRL27.fna'**	*Lactobacillus kefiranofaciens* subsp. *kefirgranum* JCM 8572	26.4	[23.0 - 30.0]	23.4	[21.1 - 25.8]	24.7	[21.8 - 27.8]	0.11
**'LcRL14.fna'**	*Lactobacillus kefiranofaciens* ATCC 43761	26.3	[23.0 - 30.0]	23.4	[21.1 - 25.9]	24.6	[21.8 - 27.7]	0.39
**'LcRL20.fna'**	*Lactobacillus acidophilus* NBRC 13951	27.5	[24.2 - 31.2]	23.3	[21.0 - 25.7]	25.5	[22.6 - 28.6]	1.97
**'LcRL31.fna'**	*Lactobacillus kefiranofaciens* subsp. *kefirgranum* JCM 8572	27.8	[24.5 - 31.5]	23.3	[21.0 - 25.8]	25.8	[22.9 - 28.9]	0.16
**'LcRL24.fna'**	*Lactobacillus ultunensis* DSM 16047	27.9	[24.6 - 31.6]	23.3	[21.0 - 25.7]	25.8	[23.0 - 28.9]	0.84
**'LcRL32.fna'**	*Lactobacillus ultunensis* DSM 16047	28.5	[25.1 - 32.1]	23.3	[21.0 - 25.8]	26.2	[23.3 - 29.3]	1.37
**'LcRL26.fna'**	*Lactobacillus acidophilus* NBRC 13951	28.4	[25.0 - 32.0]	23.3	[21.0 - 25.7]	26.2	[23.3 - 29.3]	2.85
**'LcRL16.fna'**	*Lactobacillus ultunensis* DSM 16047	26.8	[23.5 - 30.5]	23.3	[21.0 - 25.8]	25.0	[22.1 - 28.1]	0.68
**'LcRL26.fna'**	*Lactobacillus ultunensis* DSM 16047	28.7	[25.3 - 32.3]	23.3	[21.1 - 25.8]	26.4	[23.5 - 29.5]	1.52

Query	Subject	d_0	C.I. d_0	d_4	C.I. d_4	d_6	C.I. d_6	Diff. G+C Percent
**'LcRL13.fna'**	*Lactobacillus acidophilus* NBRC 13951	28.8	[25.4 - 32.4]	23.3	[21.0 - 25.7]	26.5	[23.6 - 29.6]	2.56
**'LcRL17.fna'**	*Lactobacillus kefiranofaciens* ATCC 43761	27.1	[23.7 - 30.7]	23.3	[21.0 - 25.8]	25.2	[22.3 - 28.3]	1.19
**'LcRL24.fna'**	*Lactobacillus kefiranofaciens* subsp. *kefirgranum* JCM 8572	27.5	[24.1 - 31.1]	23.3	[21.0 - 25.8]	25.5	[22.6 - 28.6]	0.65
**'LcRL14.fna'**	*Lactobacillus ultunensis* DSM 16047	27.0	[23.6 - 30.6]	23.3	[21.0 - 25.8]	25.1	[22.2 - 28.2]	0.89
**'LcRL19.fna'**	*Lactobacillus ultunensis* DSM 16047	28.4	[25.1 - 32.1]	23.3	[21.0 - 25.8]	26.2	[23.3 - 29.3]	1.76
**'LcRL21.fna'**	*Lactobacillus ultunensis* DSM 16047	26.9	[23.6 - 30.6]	23.3	[21.0 - 25.7]	25.1	[22.2 - 28.2]	1.29
**'LcRL27.fna'**	*Lactobacillus ultunensis* DSM 16047	26.8	[23.5 - 30.5]	23.3	[21.0 - 25.8]	25.0	[22.2 - 28.1]	1.38
**'LcRL30.fna'**	*Lactobacillus kefiranofaciens* subsp. *kefirgranum* JCM 8572	28.0	[24.7 - 31.7]	23.3	[21.0 - 25.7]	25.9	[23.0 - 29.0]	0.45
**'LcRL20.fna'**	*Lactobacillus kefiranofaciens* subsp. *kefirgranum* JCM 8572	27.3	[23.9 - 30.9]	23.3	[21.0 - 25.8]	25.3	[22.5 - 28.4]	0.86
**'LcRL21.fna'**	*Lactobacillus kefiranofaciens* subsp. *kefirgranum* JCM 8572	26.5	[23.1 - 30.1]	23.3	[21.0 - 25.7]	24.7	[21.9 - 27.8]	0.2
**'LcRL28.fna'**	*Lactobacillus acidophilus* NBRC 13951	28.8	[25.5 - 32.5]	23.3	[21.0 - 25.7]	26.5	[23.6 - 29.6]	2.71
**'LcRL23.fna'**	*Lactobacillus kefiranofaciens* ATCC 43761	27.5	[24.2 - 31.2]	23.3	[21.1 - 25.8]	25.5	[22.7 - 28.6]	0.34
**'LcRL15.fna'**	*Lactobacillus kefiranofaciens* ATCC 43761	27.8	[24.4 - 31.4]	23.3	[21.0 - 25.8]	25.7	[22.9 - 28.8]	0.01
**'LcRL32.fna'**	*Lactobacillus kefiranofaciens* subsp. *kefirgranum* JCM 8572	28.0	[24.6 - 31.6]	23.3	[21.0 - 25.8]	25.9	[23.0 - 29.0]	0.12
**'LcRL31.fna'**	*Lactobacillus ultunensis* DSM 16047	28.1	[24.8 - 31.7]	23.3	[21.0 - 25.7]	26.0	[23.1 - 29.1]	1.33
**'LcRL11.fna'**	*Lactobacillus acidophilus* NBRC 13951	28.9	[25.5 - 32.5]	23.3	[21.0 - 25.7]	26.5	[23.7 - 29.7]	2.37
**'LcRL14.fna'**	*Lactobacillus kefiranofaciens* subsp. *kefirgranum* JCM 8572	26.6	[23.3 - 30.3]	23.3	[21.0 - 25.8]	24.8	[22.0 - 27.9]	0.6
**'LcRL19.fna'**	*Lactobacillus kefiranofaciens* subsp. *kefirgranum* JCM 8572	28.1	[24.7 - 31.7]	23.3	[21.1 - 25.8]	25.9	[23.1 - 29.1]	0.27
**'LcRL25.fna'**	*Lactobacillus kefiranofaciens* ATCC 43761	27.7	[24.3 - 31.3]	23.3	[21.0 - 25.8]	25.7	[22.8 - 28.8]	0.27
**'LcRL20.fna'**	*Lactobacillus ultunensis* DSM 16047	27.5	[24.1 - 31.1]	23.3	[21.0 - 25.8]	25.5	[22.6 - 28.6]	0.63
**'LcRL14.fna'**	*Lactobacillus acidophilus* NBRC 13951	26.6	[23.2 - 30.2]	23.2	[20.9 - 25.7]	24.8	[21.9 - 27.9]	2.23
**'LcRL25.fna'**	*Lactobacillus kefiranofaciens* subsp. *kefirgranum* JCM 8572	28.0	[24.7 - 31.7]	23.2	[20.9 - 25.7]	25.9	[23.0 - 29.0]	0.48
**'LcRL21.fna'**	*Lactobacillus acidophilus* NBRC 13951	26.9	[23.5 - 30.5]	23.2	[20.9 - 25.7]	25.0	[22.2 - 28.1]	2.63

Query	Subject	d_0	C.I. d_0	d_4	C.I. d_4	d_6	C.I. d_6	Diff. G+C Percent
**'LcRL19.fna'**	*Lactobacillus acidophilus* NBRC 13951	28.4	[25.1 - 32.0]	23.2	[20.9 - 25.7]	26.2	[23.3 - 29.3]	3.09
**'LcRL30.fna'**	*Lactobacillus ultunensis* DSM 16047	28.4	[25.1 - 32.1]	23.2	[20.9 - 25.7]	26.2	[23.3 - 29.3]	1.04
**'LcRL25.fna'**	*Lactobacillus acidophilus* NBRC 13951	28.3	[25.0 - 31.9]	23.2	[20.9 - 25.6]	26.1	[23.2 - 29.2]	2.35
**'LcRL15.fna'**	*Lactobacillus acidophilus* NBRC 13951	28.1	[24.7 - 31.7]	23.2	[20.9 - 25.7]	25.9	[23.1 - 29.0]	2.63
**'LcRL23.fna'**	*Lactobacillus ultunensis* DSM 16047	28.5	[25.1 - 32.1]	23.2	[20.9 - 25.7]	26.2	[23.4 - 29.4]	0.94
**'LcRL17.fna'**	*Lactobacillus kefiranofaciens* subsp. *kefirgranum* JCM 8572	27.4	[24.1 - 31.1]	23.2	[20.9 - 25.7]	25.4	[22.6 - 28.5]	0.98
**'LcRL31.fna'**	*Lactobacillus acidophilus* NBRC 13951	28.2	[24.8 - 31.8]	23.2	[20.9 - 25.6]	26.0	[23.1 - 29.1]	2.66
**'LcRL24.fna'**	*Lactobacillus acidophilus* NBRC 13951	27.9	[24.5 - 31.5]	23.2	[21.0 - 25.7]	25.8	[22.9 - 28.9]	2.17
**'LcRL33.fna'**	*Lactobacillus acidophilus* NBRC 13951	28.0	[24.6 - 31.6]	23.2	[20.9 - 25.7]	25.8	[23.0 - 28.9]	2.34
**'LcRL25.fna'**	*Lactobacillus ultunensis* DSM 16047	28.2	[24.8 - 31.8]	23.2	[21.0 - 25.7]	26.0	[23.1 - 29.1]	1.01
**'LcRL29.fna'**	*Lactobacillus acidophilus* NBRC 13951	28.8	[25.4 - 32.4]	23.2	[20.9 - 25.7]	26.5	[23.6 - 29.6]	2.58
**'LcRL27.fna'**	*Lactobacillus acidophilus* NBRC 13951	26.7	[23.3 - 30.3]	23.2	[20.9 - 25.7]	24.9	[22.0 - 28.0]	2.71
**'LcRL32.fna'**	*Lactobacillus acidophilus* NBRC 13951	28.3	[24.9 - 31.9]	23.2	[20.9 - 25.6]	26.1	[23.2 - 29.2]	2.71
**'LcRL23.fna'**	*Lactobacillus kefiranofaciens* subsp. *kefirgranum* JCM 8572	27.9	[24.5 - 31.5]	23.2	[20.9 - 25.7]	25.8	[22.9 - 28.9]	0.55
**'LcRL17.fna'**	*Lactobacillus ultunensis* DSM 16047	27.5	[24.2 - 31.2]	23.2	[20.9 - 25.7]	25.5	[22.7 - 28.6]	2.47
**'LcRL23.fna'**	*Lactobacillus acidophilus* NBRC 13951	28.5	[25.2 - 32.1]	23.2	[20.9 - 25.6]	26.2	[23.4 - 29.4]	2.28
**'LcRL30.fna'**	*Lactobacillus acidophilus* NBRC 13951	28.1	[24.8 - 31.8]	23.2	[20.9 - 25.7]	26.0	[23.1 - 29.1]	2.38
**'LcRL16.fna'**	*Lactobacillus acidophilus* NBRC 13951	26.5	[23.2 - 30.1]	23.2	[20.9 - 25.7]	24.7	[21.9 - 27.8]	2.02
**'LcRL17.fna'**	*Lactobacillus acidophilus* NBRC 13951	27.3	[23.9 - 30.9]	23.2	[20.9 - 25.7]	25.3	[22.5 - 28.4]	3.81
**'LcRL15.fna'**	*Lactobacillus kefiranofaciens* subsp. *kefirgranum* JCM 8572	28.1	[24.8 - 31.7]	23.2	[20.9 - 25.7]	26.0	[23.1 - 29.1]	0.2
**'LcRL15.fna'**	*Lactobacillus ultunensis* DSM 16047	28.4	[25.0 - 32.0]	23.2	[20.9 - 25.7]	26.2	[23.3 - 29.3]	1.29

Table 4: Strains in your dataset

Joint dataset of automatically determined closest type strains (if this mode was chosen), manually selected type strains (if selected accordingly) and the provided user strains, if provided (marked in yellow).

Strain	Authority	Other deposits	Synonyms	Base pairs	Percent G+C	No. proteins	Goldstamp	Bioproject accession	Biosample accession	Assembly accession	IMG OID
Lactobacillus suntoryeus LMG 22464	Cachat and Priest 2005	NCIMB 14005; SA	*Lactobacillus suntoryeus*	1760 061	36.5	1825	Gp0131240	PRJNA224116	SAMN02797793	GCF_001437535	
Lactobacillus kefiranofaciens ATCC 43761	Fujisawa et al. 1988 emend. Vancanneyt et al. 2004	LMG 19149; CCUG 32248; DSM 5016; JCM 6985; CIP 103307; strain WT-2B	*Lactobacillus kefiranofaciens*; *Lactobacillus kefiranofaciens* subsp. *kefiranofaciens*	2281 817	37.2	2367	Gp0099413	PRJNA257853	SAMN02983011	GCA_900103655	2597490363
Lactobacillus helveticus DSM 20075	(Orla-Jensen 1919) Bergey et al. 1925	LMG 13555; LMG 6413; NRRL B-4526; CCUG 30139; ATCC 15009; JCM 1120; IFO 15019; NBRC 15019; CIP 103146	*Lactobacillus helveticus*; *Thermobacterium helveticum*	1804 595	36.8	2078	Gp0003635	PRJNA34619	SAMN00139430	GCA_000160855	645951865
Lactobacillus ultunensis DSM 16047	Roos et al. 2005	LMG 22117; CCUG 48460; JCM 16177; Kx146C1	*Lactobacillus ultunensis*	2159 701	35.9	2210	Gp0003665	PRJNA31505	SAMN00001484	GCA_000159415	643886047

Strain	Authority	Other deposits	Synonyms	Base pairs	Percent G+C	No. proteins	Goldstamp	Bioproject accession	Biosample accession	Assembly accession	IMG OID
Lactobacillus acidophilus NBRC 13951	(Moro 1900) Hansen and Mocquot 1970	LMG 13550; LMG 9433; BCRC 10695; NRRL B-4495; CCUG 5917; DSM 20079; ATCC 4356; NCTC 12980; JCM 1132; IFO 13951; VKM B-1660; CIP 76.13	*Bacillus acidophilus*; *Lactobacillus acidophilus*	1955 274	34.6	1873	Gp0075770	PRJDB1353	SAMD00046914	GCA_001591845	
Lactobacillus kitasatonis JCM 1039	Mukai et al. 2003	KCTC 3155; DSM 16761	*Lactobacillus kitasatonis*	1902 496	37.5	2050	Gp0026726	PRJDB640	SAMD00016339	GCA_000615285	2565956592
Lactobacillus gallinarum JCM 2011	Fujisawa et al. 1992	LMG 9435; CCUG 30724; DSM 10532; ATCC 33199; CIP 103611; VPI 1294	*Lactobacillus gallinarum*	1918 774	36.5	2076	Gp0091989	PRJDB621	SAMD00003603	GCA_000614735	
Lactobacillus kefiranofaciens subsp. *kefirgranum* JCM 8572	(Takizawa et al. 1994) Vancanneyt et al. 2004	LMG 15132; CCUG 39467; CCUG 49353; DSM 10550; ATCC 51647; CIP 104241; GCL 1701	*Lactobacillus kefiranofaciens* subsp. *kefirgranum*; *Lactobacillus kefirgranum*	2065 566	37.4	2918	Gp0093297	PRJDB772	SAMD00000473	GCA_001311335	
Lactobacillus crispatus JCM 1185	(Brygoo and Aladame 1953) Moore and Holdeman 1970 emend. Cato et al. 1983	LMG 9479; CCUG 30722; DSM 20584; ATCC 33820; CIP 102990; CIPP II; VPI 3199	*Eubacterium crispatum*; *Lactobacillus crispatus*	2033 995	36.6	3305	Gp0093095	PRJDB800	SAMD00000420	GCA_001311685	

Strain	Authority	Other deposits	Synonyms	Base pairs	Percent G+C	No. proteins	Goldstamp	Bioproject accession	Biosample accession	Assembly accession	IMG OID
Lactobacillus amylovorus DSM 20531	Nakamura 1981	LMG 9496; NRRL B-4540; CCUG 27201; ATCC 33620; JCM 1126; CIP 102989; NCAIM B.01458	*Lactobacillus amylovorus*	2016 257	37.8	2045	Gp0130192	PRJNA224116	SAMN02369422	GCF_001433985	
Lactobacillus sobrius DSM 16698	Konstantinov et al. 2006	1; NCCB 100067; OTU171-001	*Lactobacillus sobrius*	1992 054	37.8	1950	Gp0131222	PRJNA224116	SAMN02797775	GCF_001437365	
LcRL11.fna				2184 826	37.0	2409					
LcRL13.fna				2245 186	37.2	2560					
LcRL14.fna				2607 418	36.8	2971					
LcRL15.fna				2327 044	37.2	2634					
LcRL16.fna				2597 400	36.6	2918					
LcRL17.fna				2462 754	38.4	2961					
LcRL19.fna				2310 723	37.7	2668					
LcRL20.fna				2439 235	36.6	2736					
LcRL21.fna				2569 645	37.2	2953					
LcRL23.fna				2327 357	36.9	2558					
LcRL24.fna				2392 313	36.8	2631					
LcRL25.fna				2350 093	36.9	2594					

Strain	Authority	Other deposits	Synonyms	Base pairs	Percent G+C	No. proteins	Goldstamp	Bioproject accession	Biosample accession	Assembly accession	IMG OID
LcRL26.fna				2274 027	37.4	2638					
LcRL27.fna				2602 691	37.3	3030					
LcRL28.fna				2240 036	37.3	2550					
LcRL29.fna				2248 310	37.2	2559					
LcRL30.fna				2322 358	37.0	2654					
LcRL31.fna				2396 657	37.3	2826					
LcRL32.fna				2373 341	37.3	2770					
LcRL33.fna				2401 588	36.9	2771					

Methods, Results and References

The genome sequence data were uploaded to the Type (Strain) Genome Server (TYGS), a free bioinformatics platform available under https://tygs.dsmz.de, for a whole genome-based taxonomic analysis [1]. The results were provided by the TYGS on 2020-05-25. In brief, the TYGS analysis was subdivided into the following steps:

Determination of closely related type strains

Determination of closest type strain genomes was done in two complementary ways: First, all user genomes were compared against all type strain genomes available in the TYGS database via the MASH algorithm, a fast approximation of intergenomic relatedness [2], and, the ten type strains with the smallest MASH distances chosen per user genome. Second, an additional set of ten closely related type strains was determined via the 16S rDNA gene sequences. These were extracted from the user genomes using RNAmmer [3] and each sequence was subsequently BLASTed [4] against the 16S rDNA gene sequence of each of the currently 11767 type strains available in the TYGS database. This was used as a proxy to find the best 50 matching type strains (according to the bitscore) for each user genome and to subsequently calculate precise distances using the Genome BLAST Distance Phylogeny approach (GBDP) under the algorithm 'coverage' and distance formula d_5 [5]. These distances were finally used to determine the 10 closest type strain genomes for each of the user genomes.

Pairwise comparison of genome sequences

All pairwise comparisons among the set of genomes were conducted using GBDP and accurate intergenomic distances inferred under the algorithm 'trimming' and distance formula d_5 [5]. 100 distance replicates were calculated each. Digital DDH values and confidence intervals were calculated using the recommended settings of the GGDC 2.1 [5].

Phylogenetic inference

The resulting intergenomic distances were used to infer a balanced minimum evolution tree with branch support via FASTME 2.1.4 including SPR postprocessing [6]. Branch support was inferred from 100 pseudo-bootstrap replicates each. The trees were rooted at the midpoint [7] and visualized with PhyD3 [8].

Type-based species and subspecies clustering

The type-based species clustering using a 70% dDDH radius around each of the 11 type strains was done as previously described [1]. The resulting groups are shown in Table 1 and 4. Subspecies clustering was done using a 79% dDDH threshold as previously introduced [9].

Results

Type-based species and subspecies clustering

The resulting species and subspecies clusters are listed in Table 4, whereas the taxonomic identification of the query strains is found in Table 1. Briefly, the clustering yielded 8 species clusters and the provided query strains were assigned to 1 of these. Moreover, user strains were located in 1 of 10 subspecies clusters.

Figure caption genome tree

Figure 1. Tree inferred with FastME 2.1.6.1 [6] from GBDP distances calculated from genome sequences. The branch lengths are scaled in terms of GBDP distance formula d_5. The numbers above branches are GBDP pseudo-bootstrap support values > 60 % from 100 replications, with an average branch support of 38.1 %. The tree was rooted at the midpoint [7].

Figure caption SSU tree

Figure 2. Tree inferred with FastME 2.1.6.1 [6] from GBDP distances calculated from 16S rDNA gene sequences. The branch lengths are scaled in terms of GBDP distance formula d_5. The numbers above branches are GBDP pseudo-bootstrap support values > 60 % from 100 replications, with an average branch support of 37.8 %. The tree was rooted at the midpoint [7].

References

[1] Meier-Kolthoff JP, Göker M. TYGS is an automated high-throughput platform for state-of-the-art genome-based taxonomy. Nat. Commun. 2019;10: 2182. DOI: 10.1038/s41467-019-10210-3

[2] Ondov BD, Treangen TJ, Melsted P, et al. Mash: Fast genome and metagenome distance estimation using MinHash. Genome Biol 2016;17: 1–14. DOI: 10.1186/s13059-016-0997-x

[3] Lagesen K, Hallin P. RNAmmer: consistent and rapid annotation of ribosomal RNA genes. Nucleic Acids Res. Oxford Univ Press; 2007;35: 3100–3108. DOI: 10.1093/nar/gkm160

[4] Camacho C, Coulouris G, Avagyan V, Ma N, Papadopoulos J, Bealer K, et al. BLAST+: architecture and applications. BMC Bioinformatics. 2009;10: 421. DOI: 10.1186/1471-2105-10-421

[5] Meier-Kolthoff JP, Auch AF, Klenk H-P, Göker M. Genome sequence-based species delimitation with confidence intervals and improved distance functions. BMC Bioinformatics. 2013;14: 60. DOI: 10.1186/1471-2105-14-60

[6] Lefort V, Desper R, Gascuel O. FastME 2.0: A comprehensive, accurate, and fast distance-based phylogeny inference program. Mol Biol Evol. 2015;32: 2798–2800. DOI: 10.1093/molbev/msv150

[7] Farris JS. Estimating phylogenetic trees from distance matrices. Am Nat. 1972;106: 645–667.

[8] Kreft L, Botzki A, Coppens F, Vandepoele K, Van Bel M. PhyD3: A phylogenetic tree viewer with extended phyloXML support for functional genomics data visualization. Bioinformatics. 2017;33: 2946–2947. DOI: 10.1093/bioinformatics/btx324

[9] Meier-Kolthoff JP, Hahnke RL, Petersen J, Scheuner C, Michael V, Fiebig A, et al. Complete genome sequence of DSM 30083^{T}, the type strain (U5/41^{T}) of *Escherichia coli*, and a proposal for delineating subspecies in microbial taxonomy. Stand Genomic Sci. 2014;9: 2. DOI: 10.1186/1944-3277-9-2

TYPE (STRAIN) GENOME SERVER

JOB SUMMARY

PRINT DATE: 2020-05-25 04:11:00 +0200

JOB ID: b8084950-9088-4cd4-b817-01981c5ea7fe

RESULT PAGE: https://tygs.dsmz.de/user_results/show?guid=b8084950-9088-4cd4-b817-01981c5ea7fe

Table 1: Phylogenies

Publication-ready versions of both the genome-scale GBDP tree and the 16S rRNA gene sequence tree can be customized and exported either in SVG (vector graphic) or PNG format from within the phylogeny viewers in your TYGS result page. For publications the **SVG format is recommended** because it is lossless, always keeps its high resolution and can also be easily converted to other popular formats such as PDF or EPS. Please follow the link provided above!

Table 2: Identification

The below list contains the result of the TYGS species identification routine.

Explanation of remarks that might occur in the below table:

remark [R1]: The TYGS type strain database is automatically updated on an almost daily basis. However, if a particular type strain genome is not available in the TYGS database, this can have several reasons which are detailed in the FAQ. You can request an extended 16S rRNA gene analysis via the 16S tree viewer found in your result page to detect **not yet genome-sequenced** type strains relevant for your study.

remark [R2]: > 70% dDDH value (formula d_4) and (almost) minimal dDDH values for gene-content formulae d_0 and d_6 indicate a potentially unreliable identification result and should thus be checked via the 16S rRNA gene sequence similarity. Such strong deviations can, in principle, be caused by sequence contamination.

remark [R3]: G+C content difference of > 1 % indicates a potentially unreliable identification result because within species G+C content varies no more than 1 %, if computed from genome sequences (PMID: 24505073).

Strain	Conclusion	Identification result	Remark
**'LcSJ3CUS'**	belongs to known species	*Lactobacillus crispatus*	
**'LcV4'**	belongs to known species	*Lactobacillus crispatus*	
**'LcVMC1'**	belongs to known species	*Lactobacillus crispatus*	
**'LcVMC2'**	belongs to known species	*Lactobacillus crispatus*	
**'LcVMC3'**	belongs to known species	*Lactobacillus crispatus*	
**'LcVMC4'**	belongs to known species	*Lactobacillus crispatus*	
**'LcVMC5'**	belongs to known species	*Lactobacillus crispatus*	
**'LcVMC6'**	belongs to known species	*Lactobacillus crispatus*	
**'LcVMC7'**	belongs to known species	*Lactobacillus crispatus*	
**'LcVMC8'**	belongs to known species	*Lactobacillus crispatus*	

Table 3: Pairwise comparisons of user genomes vs. type-strain genomes

The following table contains the pairwise dDDH values between your user genomes and the selected type-strain genomes. The dDDH values are provided along with their confidence intervals (C.I.) for the three different GBDP formulas:

- formula d_0 (a.k.a. GGDC formula 1): length of all HSPs divided by total genome length
- formula d_4 (a.k.a. GGDC formula 2): sum of all identities found in HSPs divided by overall HSP length
- formula d_6 (a.k.a. GGDC formula 3): sum of all identities found in HSPs divided by total genome length

Note: Formula d_4 is independent of genome length and is thus robust against the use of incomplete draft genomes. For other reasons for preferring formula d_4, see the FAQ.

Query	Subject	d_0	C.I. d_0	d_4	C.I. d_4	d_6	C.I. d_6	Diff. G+C Percent
**'LcSJ3CUS.fna'**	**'LcV4.fna'**	91.5	[88.5 - 93.8]	97.8	[96.9 - 98.5]	94.7	[92.7 - 96.2]	0.27
**'LcSJ3CUS.fna'**	**'LcVMC1.fna'**	95.5	[93.3 - 97.0]	97.4	[96.4 - 98.2]	97.3	[96.0 - 98.2]	0.05
**'LcV4.fna'**	**'LcVMC1.fna'**	87.3	[83.7 - 90.1]	96.9	[95.7 - 97.8]	91.5	[88.9 - 93.5]	0.22
**'LcVMC1.fna'**	**'LcVMC5.fna'**	91.7	[88.7 - 93.9]	96.8	[95.7 - 97.7]	94.7	[92.7 - 96.2]	0.1
**'LcSJ3CUS.fna'**	**'LcVMC8.fna'**	84.6	[80.9 - 87.8]	96.2	[94.9 - 97.2]	89.4	[86.5 - 91.6]	0.08
**'LcSJ3CUS.fna'**	**'LcVMC5.fna'**	91.8	[88.8 - 94.0]	96.2	[94.8 - 97.2]	94.7	[92.7 - 96.2]	0.16
**'LcSJ3CUS.fna'**	**'LcVMC2.fna'**	90.5	[87.4 - 93.0]	96.1	[94.7 - 97.1]	93.8	[91.6 - 95.4]	0.13
**'LcV4.fna'**	**'LcVMC2.fna'**	87.6	[84.1 - 90.5]	95.8	[94.3 - 96.9]	91.6	[89.1 - 93.6]	0.14
**'LcSJ3CUS.fna'**	**'LcVMC6.fna'**	87.1	[83.5 - 89.9]	95.5	[94.0 - 96.6]	91.1	[88.5 - 93.2]	0.04
**'LcV4.fna'**	**'LcVMC8.fna'**	80.5	[76.6 - 83.9]	95.4	[93.9 - 96.6]	85.9	[82.8 - 88.5]	0.19
**'LcVMC1.fna'**	**'LcVMC8.fna'**	82.9	[79.1 - 86.2]	95.3	[93.8 - 96.5]	87.8	[84.8 - 90.3]	0.03
**'LcVMC1.fna'**	**'LcVMC6.fna'**	86.4	[82.8 - 89.4]	95.1	[93.6 - 96.3]	90.6	[87.9 - 92.7]	0.01
**'LcV4.fna'**	**'LcVMC6.fna'**	82.2	[78.4 - 85.5]	94.9	[93.3 - 96.1]	87.2	[84.2 - 89.7]	0.23
**'LcVMC6.fna'**	**'LcVMC8.fna'**	94.2	[91.7 - 96.0]	94.6	[92.9 - 95.9]	96.1	[94.5 - 97.3]	0.04
**'LcV4.fna'**	**'LcVMC5.fna'**	87.5	[84.0 - 90.4]	94.6	[92.9 - 95.9]	91.3	[88.8 - 93.4]	0.12
**'LcVMC1.fna'**	**'LcVMC2.fna'**	91.6	[88.6 - 93.9]	94.5	[92.8 - 95.8]	94.3	[92.2 - 95.9]	0.08
**'LcVMC5.fna'**	**'LcVMC6.fna'**	87.1	[83.6 - 90.0]	94.4	[92.7 - 95.7]	91.0	[88.4 - 93.1]	0.12
**'LcVMC2.fna'**	**'LcVMC6.fna'**	86.8	[83.2 - 89.7]	94.1	[92.3 - 95.5]	90.7	[88.1 - 92.8]	0.09
**'LcVMC4.fna'**	**'LcVMC7.fna'**	90.1	[86.8 - 92.6]	94.0	[92.2 - 95.4]	93.1	[90.8 - 94.9]	0.15
**'LcVMC5.fna'**	**'LcVMC8.fna'**	88.4	[85.0 - 91.2]	93.6	[91.7 - 95.0]	91.9	[89.4 - 93.8]	0.08
**'LcVMC2.fna'**	**'LcVMC5.fna'**	93.4	[90.7 - 95.3]	93.5	[91.6 - 95.0]	95.4	[93.6 - 96.8]	0.02
**'LcVMC2.fna'**	**'LcVMC8.fna'**	88.4	[85.0 - 91.1]	92.7	[90.7 - 94.2]	91.7	[89.2 - 93.7]	0.05

Query	Subject	d_0	C.I. d_0	d_4	C.I. d_4	d_6	C.I. d_6	Diff. G+C Percent
**'LcSJ3CUS.fna'**	**'LcVMC7.fna'**	86.7	[83.1 - 89.6]	90.5	[88.3 - 92.4]	90.1	[87.3 - 92.3]	0.12
**'LcV4.fna'**	**'LcVMC7.fna'**	79.1	[75.1 - 82.5]	90.4	[88.1 - 92.2]	83.9	[80.6 - 86.7]	0.39
**'LcSJ3CUS.fna'**	**'LcVMC4.fna'**	84.5	[80.7 - 87.6]	90.3	[88.0 - 92.2]	88.3	[85.4 - 90.7]	0.03
**'LcVMC6.fna'**	**'LcVMC7.fna'**	76.7	[72.8 - 80.3]	90.0	[87.7 - 92.0]	81.8	[78.5 - 84.7]	0.16
**'LcVMC4.fna'**	**'LcVMC6.fna'**	80.5	[76.6 - 83.9]	89.9	[87.6 - 91.8]	85.0	[81.8 - 87.7]	0.01
**'LcVMC1.fna'**	**'LcVMC4.fna'**	81.8	[77.9 - 85.1]	89.9	[87.6 - 91.8]	86.0	[82.9 - 88.7]	0.02
**'LcVMC1.fna'**	**'LcVMC7.fna'**	82.5	[78.7 - 85.8]	89.7	[87.4 - 91.7]	86.7	[83.6 - 89.2]	0.17
**'LcVMC5.fna'**	**'LcVMC7.fna'**	80.3	[76.4 - 83.7]	89.5	[87.1 - 91.5]	84.8	[81.6 - 87.5]	0.28
**'LcVMC7.fna'**	**'LcVMC8.fna'**	73.2	[69.2 - 76.8]	88.5	[86.1 - 90.6]	78.4	[75.0 - 81.5]	0.2
**'LcVMC2.fna'**	**'LcVMC7.fna'**	77.7	[73.8 - 81.3]	87.9	[85.4 - 90.0]	82.3	[79.0 - 85.2]	0.25
**'LcV4.fna'**	**'LcVMC4.fna'**	85.3	[81.6 - 88.4]	86.9	[84.3 - 89.1]	88.4	[85.5 - 90.8]	0.24
**'LcVMC4.fna'**	**'LcVMC8.fna'**	77.1	[73.1 - 80.6]	85.6	[82.9 - 87.9]	81.4	[78.0 - 84.3]	0.05
**'LcVMC4.fna'**	**'LcVMC5.fna'**	85.8	[82.1 - 88.8]	85.3	[82.6 - 87.7]	88.6	[85.7 - 91.0]	0.12
**'LcVMC2.fna'**	**'LcVMC4.fna'**	84.2	[80.4 - 87.3]	85.3	[82.6 - 87.7]	87.3	[84.2 - 89.8]	0.1
**'LcVMC1.fna'**	**'LcVMC3.fna'**	69.2	[65.3 - 72.9]	83.6	[80.8 - 86.1]	74.1	[70.6 - 77.3]	0.24
**'LcVMC3.fna'**	**'LcVMC7.fna'**	70.2	[66.3 - 73.9]	83.5	[80.7 - 86.0]	74.9	[71.5 - 78.1]	0.07
**'LcVMC3.fna'**	**'LcVMC4.fna'**	67.0	[63.2 - 70.7]	83.2	[80.4 - 85.7]	71.9	[68.5 - 75.2]	0.22
**'LcV4.fna'**	**'LcVMC3.fna'**	64.6	[60.8 - 68.2]	83.2	[80.4 - 85.7]	69.6	[66.2 - 72.8]	0.46
**'LcVMC3.fna'**	**'LcVMC5.fna'**	66.1	[62.2 - 69.7]	82.9	[80.0 - 85.4]	71.0	[67.5 - 74.2]	0.35
**'LcSJ3CUS.fna'**	**'LcVMC3.fna'**	71.7	[67.8 - 75.4]	81.9	[79.1 - 84.5]	76.0	[72.6 - 79.2]	0.19
**'LcVMC3.fna'**	**'LcVMC8.fna'**	61.8	[58.1 - 65.4]	81.7	[78.8 - 84.2]	66.7	[63.3 - 70.0]	0.27
**'LcVMC3.fna'**	**'LcVMC6.fna'**	62.9	[59.2 - 66.5]	81.6	[78.7 - 84.1]	67.8	[64.4 - 71.0]	0.23
**'LcVMC2.fna'**	**'LcVMC3.fna'**	64.7	[60.9 - 68.3]	81.2	[78.3 - 83.8]	69.4	[66.0 - 72.7]	0.32
**'LcVMC1.fna'**	*Lactobacillus crispatus* JCM 1185	71.9	[67.9 - 75.5]	77.8	[74.8 - 80.5]	75.5	[72.0 - 78.6]	0.22
**'LcV4.fna'**	*Lactobacillus crispatus* JCM 1185	66.2	[62.4 - 69.9]	77.8	[74.8 - 80.5]	70.3	[66.9 - 73.5]	0.44
**'LcSJ3CUS.fna'**	*Lactobacillus crispatus* JCM 1185	73.5	[69.5 - 77.1]	77.3	[74.3 - 80.0]	76.8	[73.4 - 80.0]	0.17
**'LcVMC5.fna'**	*Lactobacillus crispatus* JCM 1185	68.0	[64.1 - 71.6]	77.2	[74.2 - 80.0]	71.9	[68.4 - 75.1]	0.32

Query	Subject	d_0	C.I. d_0	d_4	C.I. d_4	d_6	C.I. d_6	Diff. G+C Percent
**'LcVMC2.fna'**	*Lactobacillus crispatus* JCM 1185	66.9	[63.1 - 70.6]	76.9	[73.9 - 79.6]	70.8	[67.4 - 74.1]	0.3
**'LcVMC6.fna'**	*Lactobacillus crispatus* JCM 1185	63.8	[60.0 - 67.4]	76.8	[73.8 - 79.5]	67.9	[64.5 - 71.1]	0.21
**'LcVMC8.fna'**	*Lactobacillus crispatus* JCM 1185	63.9	[60.1 - 67.5]	76.8	[73.8 - 79.5]	68.0	[64.6 - 71.2]	0.25
**'LcVMC4.fna'**	*Lactobacillus crispatus* JCM 1185	68.8	[64.9 - 72.4]	75.6	[72.6 - 78.4]	72.3	[68.8 - 75.5]	0.2
**'LcVMC7.fna'**	*Lactobacillus crispatus* JCM 1185	70.6	[66.6 - 74.2]	75.5	[72.5 - 78.3]	73.9	[70.4 - 77.1]	0.05
**'LcVMC3.fna'**	*Lactobacillus crispatus* JCM 1185	73.1	[69.1 - 76.7]	75.4	[72.4 - 78.2]	76.1	[72.7 - 79.3]	0.02
**'LcSJ3CUS.fna'**	*Lactobacillus gallinarum* JCM 2011	33.0	[29.6 - 36.6]	24.5	[22.2 - 27.0]	29.9	[27.0 - 33.0]	0.28
**'LcVMC4.fna'**	*Lactobacillus amylovorus* DSM 20531	31.0	[27.6 - 34.6]	24.3	[22.0 - 26.7]	28.3	[25.4 - 31.4]	0.99
**'LcVMC4.fna'**	*Lactobacillus gallinarum* JCM 2011	31.1	[27.7 - 34.7]	24.3	[22.0 - 26.8]	28.4	[25.5 - 31.5]	0.31
**'LcVMC3.fna'**	*Lactobacillus amylovorus* DSM 20531	32.9	[29.5 - 36.5]	24.3	[22.0 - 26.8]	29.8	[26.8 - 32.9]	1.21
**'LcSJ3CUS.fna'**	*Lactobacillus suntoryeus* LMG 22464	31.2	[27.8 - 34.8]	24.3	[21.9 - 26.7]	28.5	[25.6 - 31.6]	0.23
**'LcVMC1.fna'**	*Lactobacillus gallinarum* JCM 2011	31.3	[27.9 - 34.9]	24.3	[22.0 - 26.8]	28.6	[25.7 - 31.7]	0.33
**'LcVMC7.fna'**	*Lactobacillus gallinarum* JCM 2011	31.6	[28.2 - 35.2]	24.3	[22.0 - 26.8]	28.8	[25.9 - 31.9]	0.16
**'LcVMC8.fna'**	*Lactobacillus gallinarum* JCM 2011	30.0	[26.7 - 33.6]	24.3	[22.0 - 26.8]	27.7	[24.7 - 30.8]	0.36
**'LcVMC5.fna'**	*Lactobacillus gallinarum* JCM 2011	31.0	[27.7 - 34.6]	24.2	[21.9 - 26.7]	28.4	[25.5 - 31.5]	0.44
**'LcVMC8.fna'**	*Lactobacillus suntoryeus* LMG 22464	28.8	[25.4 - 32.4]	24.2	[21.9 - 26.6]	26.7	[23.8 - 29.8]	0.31
**'LcVMC2.fna'**	*Lactobacillus gallinarum* JCM 2011	30.5	[27.1 - 34.1]	24.2	[21.9 - 26.6]	28.0	[25.1 - 31.1]	0.41
**'LcV4.fna'**	*Lactobacillus gallinarum* JCM 2011	31.7	[28.3 - 35.3]	24.2	[21.9 - 26.7]	28.9	[26.0 - 32.0]	0.55
**'LcVMC4.fna'**	*Lactobacillus kitasatonis* JCM 1039	31.6	[28.2 - 35.2]	24.2	[21.9 - 26.7]	28.8	[25.8 - 31.9]	0.72
**'LcVMC3.fna'**	*Lactobacillus kitasatonis* JCM 1039	33.3	[29.9 - 36.8]	24.1	[21.8 - 26.6]	30.0	[27.0 - 33.1]	0.94
**'LcSJ3CUS.fna'**	*Lactobacillus amylovorus* DSM 20531	34.0	[30.6 - 37.5]	24.1	[21.8 - 26.6]	30.5	[27.5 - 33.6]	1.02
**'LcVMC5.fna'**	*Lactobacillus suntoryeus* LMG 22464	29.7	[26.3 - 33.3]	24.1	[21.8 - 26.6]	27.3	[24.4 - 30.4]	0.39
**'LcVMC3.fna'**	*Lactobacillus gallinarum* JCM 2011	33.2	[29.9 - 36.8]	24.1	[21.8 - 26.5]	29.9	[27.0 - 33.0]	0.09
**'LcVMC1.fna'**	*Lactobacillus kitasatonis* JCM 1039	32.5	[29.1 - 36.1]	24.1	[21.8 - 26.6]	29.4	[26.5 - 32.5]	0.7
**'LcVMC6.fna'**	*Lactobacillus gallinarum* JCM 2011	31.3	[27.9 - 34.9]	24.1	[21.8 - 26.6]	28.5	[25.6 - 31.6]	0.32
**'LcVMC7.fna'**	*Lactobacillus amylovorus* DSM 20531	30.7	[27.3 - 34.3]	24.1	[21.8 - 26.6]	28.1	[25.2 - 31.2]	1.14
**'LcSJ3CUS.fna'**	*Lactobacillus sobrius* DSM 16698	32.9	[29.6 - 36.5]	24.0	[21.7 - 26.4]	29.7	[26.8 - 32.8]	1.07

Query	Subject	d_0	C.I. d_0	d_4	C.I. d_4	d_6	C.I. d_6	Diff. G+C Percent
**'LcSJ3CUS.fna'**	*Lactobacillus kitasatonis* JCM 1039	33.2	[29.8 - 36.7]	24.0	[21.7 - 26.4]	29.9	[26.9 - 33.0]	0.75
**'LcVMC8.fna'**	*Lactobacillus amylovorus* DSM 20531	30.8	[27.5 - 34.4]	24.0	[21.7 - 26.5]	28.2	[25.3 - 31.3]	0.94
**'LcVMC3.fna'**	*Lactobacillus helveticus* DSM 20075	27.9	[24.5 - 31.5]	24.0	[21.7 - 26.4]	25.9	[23.0 - 29.0]	0.19
**'LcVMC2.fna'**	*Lactobacillus kitasatonis* JCM 1039	31.9	[28.5 - 35.5]	24.0	[21.7 - 26.4]	29.0	[26.0 - 32.1]	0.62
**'LcVMC4.fna'**	*Lactobacillus suntoryeus* LMG 22464	29.4	[26.0 - 33.0]	24.0	[21.7 - 26.5]	27.1	[24.2 - 30.2]	0.26
**'LcVMC1.fna'**	*Lactobacillus amylovorus* DSM 20531	32.2	[28.8 - 35.8]	24.0	[21.7 - 26.5]	29.2	[26.3 - 32.3]	0.97
**'LcVMC4.fna'**	*Lactobacillus helveticus* DSM 20075	26.7	[23.4 - 30.3]	24.0	[21.7 - 26.5]	25.0	[22.2 - 28.1]	0.03
**'LcVMC6.fna'**	*Lactobacillus suntoryeus* LMG 22464	29.1	[25.7 - 32.7]	24.0	[21.7 - 26.5]	26.9	[24.0 - 30.0]	0.27
**'LcVMC5.fna'**	*Lactobacillus helveticus* DSM 20075	27.0	[23.6 - 30.6]	24.0	[21.7 - 26.5]	25.2	[22.4 - 28.4]	0.16
**'LcV4.fna'**	*Lactobacillus suntoryeus* LMG 22464	30.3	[26.9 - 33.9]	24.0	[21.7 - 26.5]	27.8	[24.9 - 30.9]	0.5
**'LcVMC7.fna'**	*Lactobacillus kitasatonis* JCM 1039	32.6	[29.2 - 36.2]	24.0	[21.7 - 26.5]	29.5	[26.6 - 32.6]	0.87
**'LcVMC6.fna'**	*Lactobacillus amylovorus* DSM 20531	31.1	[27.7 - 34.7]	24.0	[21.7 - 26.4]	28.3	[25.4 - 31.4]	0.98
**'LcVMC8.fna'**	*Lactobacillus helveticus* DSM 20075	26.3	[22.9 - 29.9]	24.0	[21.7 - 26.5]	24.7	[21.9 - 27.8]	0.08
**'LcSJ3CUS.fna'**	*Lactobacillus helveticus* DSM 20075	28.2	[24.8 - 31.8]	24.0	[21.7 - 26.5]	26.2	[23.3 - 29.3]	0.0
**'LcVMC6.fna'**	*Lactobacillus kitasatonis* JCM 1039	31.2	[27.8 - 34.7]	23.9	[21.6 - 26.4]	28.4	[25.5 - 31.5]	0.71
**'LcVMC3.fna'**	*Lactobacillus sobrius* DSM 16698	32.7	[29.3 - 36.2]	23.9	[21.6 - 26.4]	29.5	[26.6 - 32.6]	1.26
**'LcVMC4.fna'**	*Lactobacillus sobrius* DSM 16698	30.7	[27.3 - 34.3]	23.9	[21.6 - 26.4]	28.1	[25.1 - 31.2]	1.04
**'LcV4.fna'**	*Lactobacillus amylovorus* DSM 20531	33.4	[30.1 - 37.0]	23.9	[21.5 - 26.3]	30.0	[27.1 - 33.1]	0.75
**'LcV4.fna'**	*Lactobacillus kitasatonis* JCM 1039	32.5	[29.1 - 36.0]	23.9	[21.6 - 26.3]	29.3	[26.4 - 32.4]	0.48
**'LcVMC2.fna'**	*Lactobacillus suntoryeus* LMG 22464	29.4	[26.0 - 33.0]	23.9	[21.6 - 26.4]	27.1	[24.2 - 30.2]	0.36
**'LcVMC2.fna'**	*Lactobacillus amylovorus* DSM 20531	31.4	[28.0 - 34.9]	23.9	[21.6 - 26.4]	28.5	[25.6 - 31.6]	0.89
**'LcVMC7.fna'**	*Lactobacillus suntoryeus* LMG 22464	30.3	[27.0 - 33.9]	23.9	[21.6 - 26.4]	27.8	[24.9 - 30.9]	0.11
**'LcVMC3.fna'**	*Lactobacillus suntoryeus* LMG 22464	30.7	[27.4 - 34.3]	23.9	[21.6 - 26.4]	28.1	[25.2 - 31.2]	0.04
**'LcVMC1.fna'**	*Lactobacillus suntoryeus* LMG 22464	30.4	[27.0 - 34.0]	23.9	[21.6 - 26.4]	27.8	[24.9 - 30.9]	0.28
**'LcVMC8.fna'**	*Lactobacillus kitasatonis* JCM 1039	30.5	[27.1 - 34.1]	23.9	[21.6 - 26.3]	27.9	[25.0 - 31.0]	0.67
**'LcVMC5.fna'**	*Lactobacillus amylovorus* DSM 20531	31.8	[28.5 - 35.4]	23.9	[21.6 - 26.4]	28.9	[26.0 - 32.0]	0.86
**'LcVMC7.fna'**	*Lactobacillus helveticus* DSM 20075	27.1	[23.8 - 30.8]	23.9	[21.6 - 26.4]	25.3	[22.5 - 28.5]	0.12

Query	Subject	d_0	C.I. d_0	d_4	C.I. d_4	d_6	C.I. d_6	Diff. G+C Percent
**'LcVMC1.fna'**	*Lactobacillus sobrius* DSM 16698	32.2	[28.8 - 35.8]	23.8	[21.5 - 26.2]	29.1	[26.2 - 32.2]	1.02
**'LcVMC5.fna'**	*Lactobacillus kitasatonis* JCM 1039	31.7	[28.3 - 35.3]	23.8	[21.5 - 26.3]	28.7	[25.8 - 31.8]	0.59
**'LcVMC1.fna'**	*Lactobacillus helveticus* DSM 20075	27.4	[24.0 - 31.0]	23.8	[21.5 - 26.3]	25.5	[22.6 - 28.6]	0.05
**'LcVMC7.fna'**	*Lactobacillus sobrius* DSM 16698	30.6	[27.3 - 34.2]	23.8	[21.5 - 26.2]	28.0	[25.1 - 31.1]	1.19
**'LcVMC6.fna'**	*Lactobacillus helveticus* DSM 20075	26.5	[23.1 - 30.1]	23.8	[21.5 - 26.3]	24.8	[22.0 - 27.9]	0.04
**'LcVMC8.fna'**	*Lactobacillus sobrius* DSM 16698	30.6	[27.2 - 34.2]	23.8	[21.5 - 26.3]	28.0	[25.0 - 31.1]	0.99
**'LcVMC2.fna'**	*Lactobacillus helveticus* DSM 20075	26.9	[23.5 - 30.5]	23.8	[21.5 - 26.3]	25.1	[22.3 - 28.2]	0.13
**'LcV4.fna'**	*Lactobacillus sobrius* DSM 16698	32.1	[28.7 - 35.6]	23.7	[21.4 - 26.2]	29.0	[26.1 - 32.1]	0.8
**'LcVMC5.fna'**	*Lactobacillus sobrius* DSM 16698	31.6	[28.3 - 35.2]	23.7	[21.4 - 26.1]	28.7	[25.8 - 31.8]	0.92
**'LcV4.fna'**	*Lactobacillus helveticus* DSM 20075	27.5	[24.1 - 31.1]	23.7	[21.4 - 26.2]	25.6	[22.7 - 28.7]	0.27
**'LcVMC6.fna'**	*Lactobacillus sobrius* DSM 16698	31.2	[27.8 - 34.8]	23.7	[21.4 - 26.2]	28.4	[25.4 - 31.5]	1.03
**'LcVMC2.fna'**	*Lactobacillus sobrius* DSM 16698	31.0	[27.6 - 34.6]	23.7	[21.4 - 26.2]	28.2	[25.3 - 31.3]	0.94
**'LcV4.fna'**	*Lactobacillus acidophilus* NBRC 13951	29.3	[25.9 - 32.9]	23.3	[21.0 - 25.8]	26.8	[23.9 - 29.9]	2.43
**'LcVMC1.fna'**	*Lactobacillus acidophilus* NBRC 13951	29.3	[25.9 - 32.9]	23.3	[21.0 - 25.7]	26.8	[23.9 - 29.9]	2.21
**'LcVMC4.fna'**	*Lactobacillus acidophilus* NBRC 13951	28.5	[25.1 - 32.1]	23.3	[21.0 - 25.8]	26.3	[23.4 - 29.4]	2.19
**'LcVMC8.fna'**	*Lactobacillus acidophilus* NBRC 13951	27.6	[24.3 - 31.3]	23.3	[21.0 - 25.8]	25.6	[22.7 - 28.7]	2.24
**'LcSJ3CUS.fna'**	*Lactobacillus kefiranofaciens* ATCC 43761	29.2	[25.8 - 32.8]	23.3	[21.0 - 25.8]	26.8	[23.9 - 29.9]	0.46
**'LcVMC5.fna'**	*Lactobacillus acidophilus* NBRC 13951	28.5	[25.1 - 32.1]	23.3	[21.0 - 25.7]	26.2	[23.3 - 29.3]	2.32
**'LcVMC2.fna'**	*Lactobacillus acidophilus* NBRC 13951	28.7	[25.3 - 32.3]	23.3	[21.0 - 25.7]	26.4	[23.5 - 29.5]	2.29
**'LcVMC8.fna'**	*Lactobacillus kefiranofaciens* ATCC 43761	27.2	[23.8 - 30.8]	23.2	[21.0 - 25.7]	25.2	[22.4 - 28.3]	0.38
**'LcVMC8.fna'**	*Lactobacillus kefiranofaciens* subsp. *kefirgranum* JCM 8572	27.4	[24.0 - 31.0]	23.2	[20.9 - 25.7]	25.4	[22.5 - 28.5]	0.59
**'LcVMC7.fna'**	*Lactobacillus acidophilus* NBRC 13951	29.1	[25.7 - 32.7]	23.2	[21.0 - 25.7]	26.7	[23.8 - 29.8]	2.04
**'LcVMC3.fna'**	*Lactobacillus acidophilus* NBRC 13951	30.9	[27.5 - 34.5]	23.2	[20.9 - 25.6]	28.0	[25.1 - 31.1]	1.97
**'LcSJ3CUS.fna'**	*Lactobacillus kefiranofaciens* subsp. *kefirgranum* JCM 8572	29.6	[26.2 - 33.2]	23.2	[20.9 - 25.7]	27.1	[24.2 - 30.2]	0.67
**'LcSJ3CUS.fna'**	*Lactobacillus acidophilus* NBRC 13951	30.3	[26.9 - 33.9]	23.2	[20.9 - 25.7]	27.6	[24.7 - 30.7]	2.16
**'LcVMC4.fna'**	*Lactobacillus kefiranofaciens* ATCC 43761	27.4	[24.0 - 31.0]	23.2	[20.9 - 25.6]	25.4	[22.5 - 28.5]	0.43

Query	Subject	d_0	C.I. d_0	d_4	C.I. d_4	d_6	C.I. d_6	Diff. G+C Percent
**'LcSJ3CUS.fna'**	*Lactobacillus ultunensis* DSM 16047	29.7	[26.3 - 33.3]	23.2	[20.9 - 25.7]	27.1	[24.2 - 30.2]	0.82
**'LcV4.fna'**	*Lactobacillus ultunensis* DSM 16047	28.9	[25.6 - 32.6]	23.1	[20.8 - 25.5]	26.5	[23.7 - 29.7]	1.1
**'LcVMC7.fna'**	*Lactobacillus kefiranofaciens* subsp. *kefirgranum* JCM 8572	27.7	[24.3 - 31.3]	23.1	[20.8 - 25.6]	25.6	[22.7 - 28.7]	0.78
**'LcVMC5.fna'**	*Lactobacillus kefiranofaciens* subsp. *kefirgranum* JCM 8572	28.4	[25.0 - 32.0]	23.1	[20.8 - 25.5]	26.1	[23.2 - 29.2]	0.51
**'LcVMC7.fna'**	*Lactobacillus kefiranofaciens* ATCC 43761	27.7	[24.4 - 31.3]	23.1	[20.8 - 25.6]	25.6	[22.8 - 28.7]	0.58
**'LcVMC4.fna'**	*Lactobacillus kefiranofaciens* subsp. *kefirgranum* JCM 8572	27.6	[24.2 - 31.2]	23.1	[20.9 - 25.6]	25.5	[22.7 - 28.6]	0.63
**'LcVMC6.fna'**	*Lactobacillus kefiranofaciens* ATCC 43761	27.5	[24.2 - 31.1]	23.1	[20.8 - 25.6]	25.5	[22.6 - 28.6]	0.42
**'LcVMC6.fna'**	*Lactobacillus ultunensis* DSM 16047	28.2	[24.8 - 31.8]	23.1	[20.8 - 25.5]	26.0	[23.1 - 29.1]	0.86
**'LcVMC2.fna'**	*Lactobacillus kefiranofaciens* ATCC 43761	27.2	[23.8 - 30.8]	23.1	[20.8 - 25.5]	25.2	[22.3 - 28.3]	0.33
**'LcVMC5.fna'**	*Lactobacillus ultunensis* DSM 16047	28.7	[25.3 - 32.3]	23.1	[20.8 - 25.6]	26.3	[23.4 - 29.4]	0.98
**'LcVMC1.fna'**	*Lactobacillus ultunensis* DSM 16047	29.2	[25.8 - 32.8]	23.1	[20.8 - 25.6]	26.8	[23.9 - 29.9]	0.88
**'LcVMC6.fna'**	*Lactobacillus acidophilus* NBRC 13951	28.7	[25.3 - 32.3]	23.1	[20.8 - 25.6]	26.3	[23.5 - 29.5]	2.2
**'LcVMC2.fna'**	*Lactobacillus ultunensis* DSM 16047	28.4	[25.0 - 32.0]	23.1	[20.8 - 25.5]	26.1	[23.2 - 29.2]	0.96
**'LcVMC8.fna'**	*Lactobacillus ultunensis* DSM 16047	27.7	[24.3 - 31.3]	23.1	[20.8 - 25.6]	25.6	[22.7 - 28.7]	0.9
**'LcVMC5.fna'**	*Lactobacillus kefiranofaciens* ATCC 43761	28.1	[24.7 - 31.7]	23.1	[20.9 - 25.6]	25.9	[23.0 - 29.0]	0.3
**'LcVMC4.fna'**	*Lactobacillus ultunensis* DSM 16047	28.3	[25.0 - 32.0]	23.1	[20.8 - 25.6]	26.1	[23.2 - 29.2]	0.86
**'LcVMC6.fna'**	*Lactobacillus kefiranofaciens* subsp. *kefirgranum* JCM 8572	27.9	[24.5 - 31.5]	23.0	[20.7 - 25.4]	25.7	[22.8 - 28.8]	0.63
**'LcV4.fna'**	*Lactobacillus kefiranofaciens* subsp. *kefirgranum* JCM 8572	28.6	[25.3 - 32.3]	23.0	[20.7 - 25.5]	26.3	[23.4 - 29.4]	0.39
**'LcVMC2.fna'**	*Lactobacillus kefiranofaciens* subsp. *kefirgranum* JCM 8572	27.5	[24.1 - 31.1]	23.0	[20.7 - 25.5]	25.4	[22.6 - 28.5]	0.53
**'LcV4.fna'**	*Lactobacillus kefiranofaciens* ATCC 43761	28.1	[24.8 - 31.7]	23.0	[20.7 - 25.4]	25.9	[23.0 - 29.0]	0.19
**'LcVMC7.fna'**	*Lactobacillus ultunensis* DSM 16047	28.6	[25.2 - 32.2]	23.0	[20.7 - 25.5]	26.2	[23.3 - 29.3]	0.7
**'LcVMC3.fna'**	*Lactobacillus ultunensis* DSM 16047	29.9	[26.5 - 33.5]	23.0	[20.7 - 25.5]	27.2	[24.3 - 30.3]	0.63
**'LcVMC1.fna'**	*Lactobacillus kefiranofaciens* subsp. *kefirgranum* JCM 8572	28.9	[25.5 - 32.5]	22.9	[20.6 - 25.3]	26.4	[23.5 - 29.5]	0.61

Query	Subject	d_0	C.I. d_0	d_4	C.I. d_4	d_6	C.I. d_6	Diff. G+C Percent
**'LcVMC1.fna'**	*Lactobacillus kefiranofaciens* ATCC 43761	28.1	[24.7 - 31.7]	22.9	[20.6 - 25.4]	25.9	[23.0 - 29.0]	0.41
**'LcVMC3.fna'**	*Lactobacillus kefiranofaciens* subsp. *kefirgranum* JCM 8572	29.8	[26.4 - 33.4]	22.8	[20.5 - 25.2]	27.1	[24.2 - 30.2]	0.86
**'LcVMC3.fna'**	*Lactobacillus kefiranofaciens* ATCC 43761	29.0	[25.6 - 32.6]	22.8	[20.5 - 25.3]	26.5	[23.6 - 29.6]	0.65

Table 4: Strains in your dataset

Joint dataset of automatically determined closest type strains (if this mode was chosen), manually selected type strains (if selected accordingly) and the provided user strains, if provided (marked in yellow).

Strain	Authority	Other deposits	Synonyms	Base pairs	Percent G+C	No. proteins	Goldstamp	Bioproject accession	Biosample accession	Assembly accession	IMG OID
Lactobacillus suntoryeus LMG 22464	Cachat and Priest 2005	NCIMB 14005; SA	*Lactobacillus suntoryeus*	1760 061	36.5	1825	Gp0131240	PRJNA224116	SAMN02797793	GCF_001437535	
Lactobacillus kefiranofaciens ATCC 43761	Fujisawa et al. 1988 emend. Vancanneyt et al. 2004	LMG 19149; CCUG 32248; DSM 5016; JCM 6985; CIP 103307; strain WT-2B	*Lactobacillus kefiranofaciens*; *Lactobacillus kefiranofaciens* subsp. *kefiranofaciens*	2281 817	37.2	2367	Gp0099413	PRJNA257853	SAMN02983011	GCA_900103655	2597490363
Lactobacillus helveticus DSM 20075	(Orla-Jensen 1919) Bergey et al. 1925	LMG 13555; LMG 6413; NRRL B-4526; CCUG 30139; ATCC 15009; JCM 1120; IFO 15019; NBRC 15019; CIP 103146	*Lactobacillus helveticus*; *Thermobacterium helveticum*	1804 595	36.8	2078	Gp0003635	PRJNA34619	SAMN00139430	GCA_000160855	645951865
Lactobacillus ultunensis DSM 16047	Roos et al. 2005	LMG 22117; CCUG 48460; JCM 16177; Kx146C1	*Lactobacillus ultunensis*	2159 701	35.9	2210	Gp0003665	PRJNA31505	SAMN00001484	GCA_000159415	643886047

Strain	Authority	Other deposits	Synonyms	Base pairs	Percent G+C	No. proteins	Goldstamp	Bioproject accession	Biosample accession	Assembly accession	IMG OID
Lactobacillus acidophilus NBRC 13951	(Moro 1900) Hansen and Mocquot 1970	LMG 13550; LMG 9433; BCRC 10695; NRRL B-4495; CCUG 5917; DSM 20079; ATCC 4356; NCTC 12980; JCM 1132; IFO 13951; VKM B-1660; CIP 76.13	*Bacillus acidophilus*; *Lactobacillus acidophilus*	1955 274	34.6	1873	Gp0075770	PRJDB1353	SAMD00046914	GCA_001591845	
Lactobacillus kitasatonis JCM 1039	Mukai et al. 2003	KCTC 3155; DSM 16761	*Lactobacillus kitasatonis*	1902 496	37.5	2050	Gp0026726	PRJDB640	SAMD00016339	GCA_000615285	2565956592
Lactobacillus gallinarum JCM 2011	Fujisawa et al. 1992	LMG 9435; CCUG 30724; DSM 10532; ATCC 33199; CIP 103611; VPI 1294	*Lactobacillus gallinarum*	1918 774	36.5	2076	Gp0091989	PRJDB621	SAMD00003603	GCA_000614735	
Lactobacillus kefiranofaciens subsp. *kefirgranum* JCM 8572	(Takizawa et al. 1994) Vancanneyt et al. 2004	LMG 15132; CCUG 39467; CCUG 49353; DSM 10550; ATCC 51647; CIP 104241; GCL 1701	*Lactobacillus kefiranofaciens* subsp. *kefirgranum*; *Lactobacillus kefirgranum*	2065 566	37.4	2918	Gp0093297	PRJDB772	SAMD00000473	GCA_001311335	
Lactobacillus crispatus JCM 1185	(Brygoo and Aladame 1953) Moore and Holdeman 1970 emend. Cato et al. 1983	LMG 9479; CCUG 30722; DSM 20584; ATCC 33820; CIP 102990; CIPP II; VPI 3199	*Eubacterium crispatum*; *Lactobacillus crispatus*	2033 995	36.6	3305	Gp0093095	PRJDB800	SAMD00000420	GCA_001311685	

Strain	Authority	Other deposits	Synonyms	Base pairs	Percent G+C	No. proteins	Goldstamp	Bioproject accession	Biosample accession	Assembly accession	IMG OID
Lactobacillus amylovorus DSM 20531	Nakamura 1981	LMG 9496; NRRL B-4540; CCUG 27201; ATCC 33620; JCM 1126; CIP 102989; NCAIM B.01458	*Lactobacillus amylovorus*	2016 257	37.8	2045	Gp0130192	PRJNA224116	SAMN02369422	GCF_001433985	
Lactobacillus sobrius DSM 16698	Konstantinov et al. 2006	1; NCCB 100067; OTU171-001	*Lactobacillus sobrius*	1992 054	37.8	1950	Gp0131222	PRJNA224116	SAMN02797775	GCF_001437365	
LcSJ3CUS.fna				2087 874	36.7	2306					
LcV4.fna				2091 889	37.0	2230					
LcVMC1.fna				2074 052	36.8	2194					
LcVMC2.fna				2201 962	36.9	2407					
LcVMC3.fna				2201 463	36.6	2192					
LcVMC4.fna				2314 219	36.8	2521					
LcVMC5.fna				2242 963	36.9	2464					
LcVMC6.fna				2343 949	36.8	2491					
LcVMC7.fna				2101 584	36.6	2215					
LcVMC8.fna				2332 118	36.8	2484					

Methods, Results and References

The genome sequence data were uploaded to the Type (Strain) Genome Server (TYGS), a free bioinformatics platform available under https://tygs.dsmz.de, for a whole genome-based taxonomic analysis [1]. The results were provided by the TYGS on 2020-05-25. In brief, the TYGS analysis was subdivided into the following steps:

Determination of closely related type strains

Determination of closest type strain genomes was done in two complementary ways: First, all user genomes were compared against all type strain genomes available in the TYGS database via the MASH algorithm, a fast approximation of intergenomic relatedness [2], and, the ten type strains with the smallest MASH distances chosen per user genome. Second, an additional set of ten closely related type strains was determined via the 16S rDNA gene sequences. These were extracted from the user genomes using RNAmmer [3] and each sequence was subsequently BLASTed [4] against the 16S rDNA gene sequence of each of the currently 11767 type strains available in the TYGS database. This was used as a proxy to find the best 50 matching type strains (according to the bitscore) for each user genome and to subsequently calculate precise distances using the Genome BLAST Distance Phylogeny approach (GBDP) under the algorithm 'coverage' and distance formula d_5 [5]. These distances were finally used to determine the 10 closest type strain genomes for each of the user genomes.

Pairwise comparison of genome sequences

All pairwise comparisons among the set of genomes were conducted using GBDP and accurate intergenomic distances inferred under the algorithm 'trimming' and distance formula d_5 [5]. 100 distance replicates were calculated each. Digital DDH values and confidence intervals were calculated using the recommended settings of the GGDC 2.1 [5].

Phylogenetic inference

The resulting intergenomic distances were used to infer a balanced minimum evolution tree with branch support via FASTME 2.1.4 including SPR postprocessing [6]. Branch support was inferred from 100 pseudo-bootstrap replicates each. The trees were rooted at the midpoint [7] and visualized with PhyD3 [8].

Type-based species and subspecies clustering

The type-based species clustering using a 70% dDDH radius around each of the 11 type strains was done as previously described [1]. The resulting groups are shown in Table 1 and 4. Subspecies clustering was done using a 79% dDDH threshold as previously introduced [9].

Results

Type-based species and subspecies clustering

The resulting species and subspecies clusters are listed in Table 4, whereas the taxonomic identification of the query strains is found in Table 1. Briefly, the clustering yielded 8 species clusters and the provided query strains were assigned to 1 of these. Moreover, user strains were located in 1 of 10 subspecies clusters.

Figure caption genome tree

Figure 1. Tree inferred with FastME 2.1.6.1 [6] from GBDP distances calculated from genome sequences. The branch lengths are scaled in terms of GBDP distance formula d_5. The numbers above branches are GBDP pseudo-bootstrap support values > 60 % from 100 replications, with an average branch support of 69.7 %. The tree was rooted at the midpoint [7].

Figure caption SSU tree

Figure 2. Tree inferred with FastME 2.1.6.1 [6] from GBDP distances calculated from 16S rDNA gene sequences. The branch lengths are scaled in terms of GBDP distance formula d_5. The numbers above branches are GBDP pseudo-bootstrap support values > 60 % from 100 replications, with an average branch support of 49.3 %. The tree was rooted at the midpoint [7].

References

[1] Meier-Kolthoff JP, Göker M. TYGS is an automated high-throughput platform for state-of-the-art genome-based taxonomy. Nat. Commun. 2019;10: 2182. DOI: 10.1038/s41467-019-10210-3

[2] Ondov BD, Treangen TJ, Melsted P, et al. Mash: Fast genome and metagenome distance estimation using MinHash. Genome Biol 2016;17: 1–14. DOI: 10.1186/s13059-016-0997-x

[3] Lagesen K, Hallin P. RNAmmer: consistent and rapid annotation of ribosomal RNA genes. Nucleic Acids Res. Oxford Univ Press; 2007;35: 3100–3108. DOI: 10.1093/nar/gkm160

[4] Camacho C, Coulouris G, Avagyan V, Ma N, Papadopoulos J, Bealer K, et al. BLAST+: architecture and applications. BMC Bioinformatics. 2009;10: 421. DOI: 10.1186/1471-2105-10-421

[5] Meier-Kolthoff JP, Auch AF, Klenk H-P, Göker M. Genome sequence-based species delimitation with confidence intervals and improved distance functions. BMC Bioinformatics. 2013;14: 60. DOI: 10.1186/1471-2105-14-60

[6] Lefort V, Desper R, Gascuel O. FastME 2.0: A comprehensive, accurate, and fast distance-based phylogeny inference program. Mol Biol Evol. 2015;32: 2798–2800. DOI: 10.1093/molbev/msv150

[7] Farris JS. Estimating phylogenetic trees from distance matrices. Am Nat. 1972;106: 645–667.

[8] Kreft L, Botzki A, Coppens F, Vandepoele K, Van Bel M. PhyD3: A phylogenetic tree viewer with extended phyloXML support for functional genomics data visualization. Bioinformatics. 2017;33: 2946–2947. DOI: 10.1093/bioinformatics/btx324

[9] Meier-Kolthoff JP, Hahnke RL, Petersen J, Scheuner C, Michael V, Fiebig A, et al. Complete genome sequence of DSM 30083^{T}, the type strain (U5/41^{T}) of *Escherichia coli*, and a proposal for delineating subspecies in microbial taxonomy. Stand Genomic Sci. 2014;9: 2. DOI: 10.1186/1944-3277-9-2
