# Supplementary figures and images for: Characterization of the first vaginal Lactobacillus crispatus genomes isolated in Brazil
Source: PeerJ. 2021 Mar 10;9:e11079. doi: 10.7717/peerj.11079 (PMC7955673; doi:10.7717/peerj.11079)

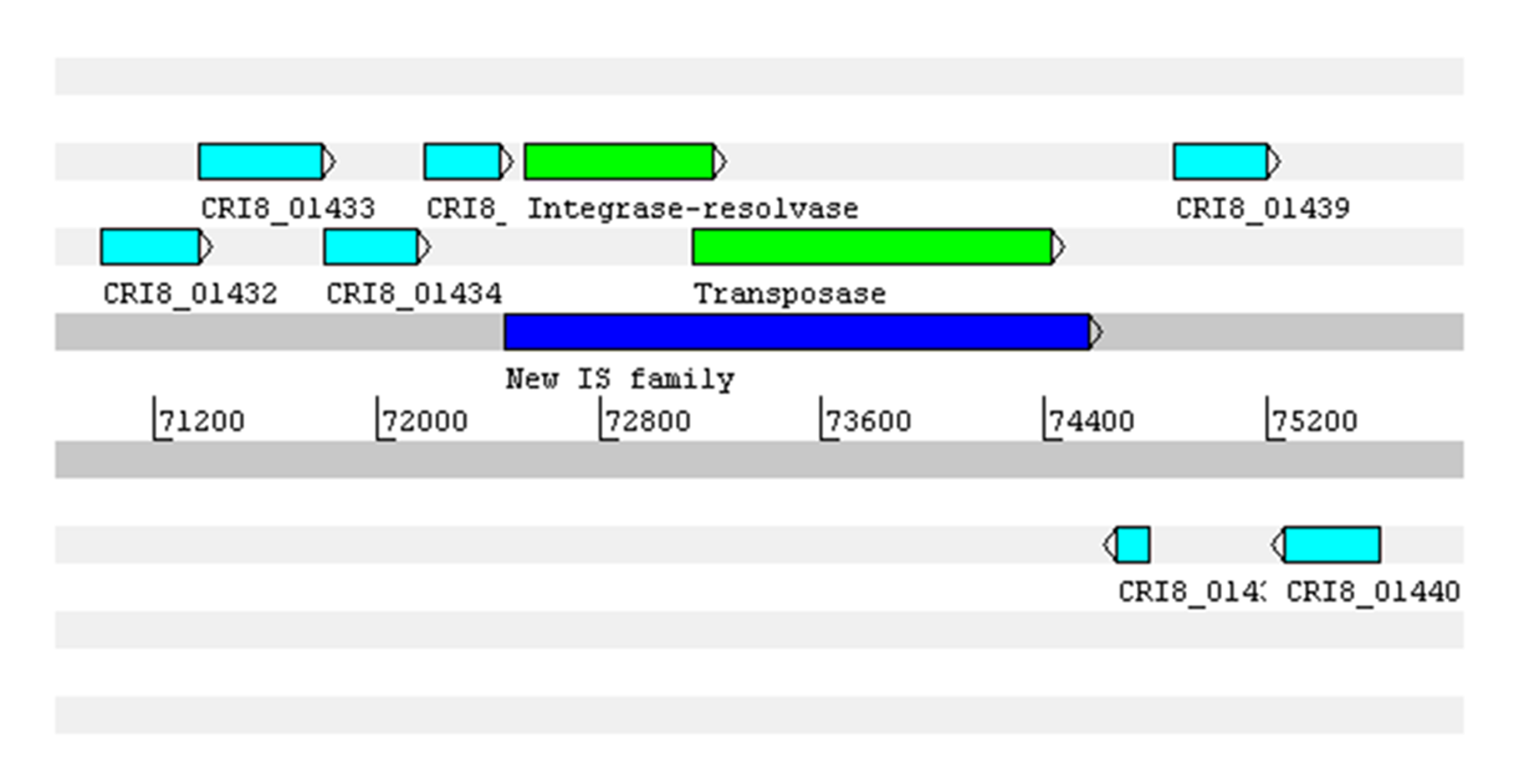

Supplement: Supplemental Information 4 — Shows the complete sequence in strain CRI8. [file peerj-09-11079-s004.png]
